# PROTOCOL: Strengthening women's empowerment and gender equality in fragile contexts towards peaceful and inclusive societies: A systematic review and meta‐analysis

**DOI:** 10.1002/cl2.1180

**Published:** 2021-07-28

**Authors:** Etienne Lwamba, Will Ridlehoover, Meital Kupfer, Shannon Shisler, Ada Sonnenfeld, Laurenz Langer, John Eyers, Sean Grant, Bidisha Barooah

**Affiliations:** ^1^ International Initiative for Impact Evaluation (3ie) London United Kingdom

## Abstract

This review builds on 3ie's (international initiative for impact evaluation) evidence gap map (EGM) of the impact evaluation and systematic review (SR) evidence base of interventions aiming to promote peaceful and inclusive societies in fragile contexts. The EGM identified a cluster of studies evaluating gender equality‐focused behaviour change communication programmes and raised interest in investigating the evidence base for understanding the role of women more broadly as agents of change in developing peaceful and inclusive societies. Building on the cluster of evidence identified in the EGM, our review will increase generalisability of findings from single studies and focus on interventions across a broad range of geographical locations, settings and populations, types of implementations and outcomes. We will also address (when possible) the identified gaps in literature regarding metaanalysis in conflict‐affected contexts. As such, we propose the following objectives: (1) The primary objective of this review is to identify, assess and synthesise evidence on the effect of gender specific and gender transformative interventions within the context of the four pillars of United Nations Security Council Resolution (UNSCR) 1325 on women's empowerment and gender equality in Fragile and Conflict Affected States/Situations (FCAS). The SR will facilitate the use of evidence in informing policy and practice decisions within the field of transition aid, particularly as it relates to gender focused programming. (2) Our second objective is to assess how these interventions contribute to inclusive and sustainable peace in conflict affected situations. We will compare the effectiveness of these different types of interventions through the lenses of their ecological level, types of impact on women's empowerment, local context of gender inequality and conflict. To achieve these objectives we aim to answer the following questions: (1) What are the impacts of gender transformative and specific interventions on women's empowerment and gender equality in FCAS? (2) What are the effects of these interventions on sustainable peace? (3) To what extent do effects vary by population group, ecological level and types of interventions? (4) What are contextual barriers to and facilitators of intervention effectiveness?

**Abbreviations**
3ie: International Initiative for Impact Evaluation
AAWAZ: Voice and Accountability programme
AFD: French Agency for Development
AG: Advisory Group
AU: African Union
BMZ: German Ministry for Economic Cooperation and Development
CDD: community driven development
CEDAW: Convention on the Elimination of Discrimination Against Women
DDR: disarmament, demobilisation, reintegration
DFID: Department for International Development
EGM: evidence gap map
EU: European Union
FCAS: Fragile and Conflict Affected States/Situations
FCDO: Foreign Commonwealth and Development Office
FTS: full text screening
GCRF: Global Challenge Research Fund
GLS: grey literature search
GPI: Global Peace Index
IDP: Internally Displaced People
IPV: Intimate Partner Violence
JLOS: justice and rule of law sector
MDGs: millenium development goals
MoFA: Ministry of Foreign Affairs
NAP: National Action Plan
OECD: Organisation of Economic Co‐operation and Development
RSS: regional stabilisation strategy
SDGs: sustainable development goals
SEA: sexual abuse and exploitation
SGBV: sexual and gender‐based violence
SR: systematic review
SRHR: Sexual and Reproductive Health Rights
TAS: title and abstract screening
UN: United Nations
UNSC(R): United Nations Security Council (Resolution)
WPS(A): Women Peace and Security (Agenda)

## 1 BACKGROUND

### 1.1 The problem, condition or issue

Despite gains made over the last decades towards equity, inclusion, and empowerment, significant gaps remain for women with respect to accessing resources, earning livelihoods, achieving legislative and political representation, and participating in important decision making processes (Cornwall, 2016; United Nations, 2010). A growing body of literature indicates that these challenges are exacerbated in contexts of conflict and fragility (Bouta et al., 2004; Buvinic, Gupta, et al., 2013; Caprioli, 2000).

This is particularly alarming when presented alongside the reality that the prevalence of fragility and conflict‐affected states is on the rise. Furthermore, in 2020, the prevalence of fragility and conflict‐affected states is on the rise. The World Bank reports that over the last 10 years, the number of fragile and conflict‐affected situations or states (FCAS) has increased from 36 to 39 countries or states, with 17 classified as a “situation of medium‐ to high‐intensity conflict” (World Bank, n.d.) The Institute for Economics and Peace reports a similar deterioration of peacefulness, finding a 0.34 percentage point drop last year in their Global Peace Index (GPI), which measures societal safety and security, ongoing domestic and international conflict, and degree of militarisation (Institute for Economics and Peace, 2020). According to the Organisation for Economic Co‐operation and Development (OECD), a staggering 23% of the world's population is living in a fragile context, including 76.5% of all those living in extreme poverty (OECD, 2020).

### 1.2 Definition of key terms

#### 1.2.1 Fragile contexts

Defining fragility is a complex and sometimes contentious challenge due to the conceptual ambiguities that characterise it (Faust et al., 2013). We adopt the same definition for situations of fragility used in the International Initiative for Impact Evaluation's (3ie) *Building peaceful societies: An evidence gap map* (Sonnenfeld et al., 2020). Situations of fragility can be understood as “…the combination of exposure to risk and insufficient coping capacity of the state, system and/or communities to manage, absorb or mitigate those risks. Fragility can lead to negative outcomes, including violence, the breakdown of institutions, displacement, humanitarian crises or other emergencies” (OECD, 2016a). The focus on situations of fragility recognises that exposure to risks and vulnerabilities is not constant, neither over time or within a state. For example, within an entrenched state that is not considered fragile, there may be communities that have the characteristics of fragility. Note that we operationalise a metrics based definition of fragility for our inclusion criteria which can be found in Methodology of Selection of Population.

#### 1.2.2 Gender equality and women's empowerment

Our SR will use Naila Kabeer's definition of women's empowerment: “a process by which women who have been denied the ability to make strategic life choices acquire such an ability” (Kabeer, 1999). This ability to exercise choice relies on three interrelated dimensions:

- Resources: material, human and social resources which serve to enhance the ability to exercise choice;
- Agency: ability to define one's goals and act upon them and operationalised decision‐making; and
- Achievement: ways of being and doing which can be realised by different individuals.

The different dimensions represent the fact that *empowerment* encompasses different categories of daily life and as such, empowerment‐focused interventions for women and girls may take the form of many different programmes, as further specified in the *interventions* section of this protocol. Overall, empowerment contributes to the overall equality between men and women, improving one's ability to make choices and live a safe and fulfilling life (Cornwall, 2000).

#### 1.2.3 Peacebuilding

The former UN Secretary‐General Boutros Boutros‐Ghali provided one of the first definitions of the concept of *peacebuilding* as “an action to identify and support structures which will tend to strengthen and solidify peace in order to avoid relapse into conflict” (Barnett et al., 2007). Similar to the *Building peaceful societies* evidence gap map (EGM), our systematic review (SR) adopts the definition of peacebuilding developed by the United Nations (UN) Secretary General's Policy Committee in 2007, which defines peacebuilding as “a range of measures aimed at reducing the risk of lapsing or relapsing into [violent] conflict, by strengthening national capacities for conflict management and laying the foundations for sustainable peace. It is a complex, long‐term process aimed at creating the necessary conditions for positive and sustainable peace by addressing the deep‐rooted structural causes of violent conflict in a comprehensive manner” (United Nations Peacebuilding Support Office, 2010).

There are three types of peacebuilding processes:

- Track I describes activities that bring parties to a conflict into direct negotiation to achieve an agreement or a resolution through official discussions between high level governmental and military leaders (includes ceasefires, peace talks, treaties, etc.);
- Track II describes activities of unofficial dialogue and problem‐solving aimed at building relationships between civil society leaders and influential individuals that can impact Track I; and
- Track III describes activities of people‐to‐people interactions at the grassroots level to encourage interaction and understanding between communities (includes meetings, media exposure, political and legal advocacy, etc.; Dudouet, 2017).

Sustainable peace relies on numerous stakeholders, at all levels and beyond the lifetime of an active conflict or crisis. Our SR will analyse the role of these stakeholders: private sector actors, the general public, civil society organisations, civil servants and service providers, and individual households.

#### 1.2.4 Human security

A broad approach to fostering peaceful societies is one that examines and understands the dangers of fragility to not only be about physical security, but human security as well. The concept of human security originates from UNOCHA's Commission on Human Security to develop a world “free from want” and “free from fear” (UNOCHA, 2009). The Berghof Foundation defines human security as “…a comprehensive, people‐centred and prevention‐oriented concept that includes protection from threats in the area of economic, food, health, environmental, personal, community and political security” (Berghof Foundation, 2019). Human security expands the concept of security beyond a state‐centric framework to focus more on the macro‐level of households and individuals. For women in particular, there are vast differences and obstacles in attaining the same level of human security as men.

#### 1.2.5 Peaceful and inclusive societies

As presented in the Building Peaceful Societies EGM, peaceful and inclusive societies cover a wider spectrum than the absence of violence and the resolution of conflict. It is then important to understand the concept of peaceful and inclusive societies as the conjunction of addressed fragility, human security, positive peace but also sustainable and inclusive governance. This target of peaceful societies has been made a priority by a series of international agencies such as the Sustainable Development Goals (Keuleers, 2016) and the UK Foreign and Commonwealth Office who identified four objectives for peaceful states and societies (Department for International Development, 2010):

1. Address the causes and effects of conflict and fragility, and build conflict resolution mechanisms
2. Support inclusive political settlements and processes
3. Develop core state functions
4. Respond to public expectations

Building on the approach initiated in the Building Peaceful Societies EGM, our review will adopt a definition of *peaceful and inclusive societies* based on the association of the following concepts:

- Addressing the causes and drivers of fragility by building sustainable economic foundations and livelihoods
- Addressing the roots causes and drivers of conflicts, strengthening social cohesion, supporting peace processes to build sustainable peace
- Supporting human security through the prevention of violence and protection of human rights and security from any form of violence
- Supporting good governance through the development of responsive and sustainable institutions and governance practices
- Developing inclusivity by addressing the the specific needs of marginalised and vulnerable groups (including women and girls).

### 1.3 The issue: Women's empowerment in FCAS

Women and girls are economically disempowered through restricted access to livelihoods and resources (Sweetman & Rowlands, 2016). The causes of this disenfranchisement extend from a lack of economic or social protection, to social norms and traditions, to legal and political barriers (Chen et al., 2006; Doss, 2013; Strickland, 2004). Examples of the kinds of systemic barriers that discourage women's economic autonomy are early and forced marriage, bureaucratic hurdles to accessing capital for entrepreneurship, sexual and gender‐based violence, and discrimination and harassment (OECD, 2016b; Perrin et al., 2019).

In addition to economic disempowerment, women are also often excluded from having a seat at the policy or decision‐making table. Women's movements are localised and often restricted to the grassroots, and when they have broader national reach, are often co‐opted by NGOs or government bodies (Jong & Kimm, 2017). Women and girls have insurmountable hurdles if they seek to enact change at all levels, from their communities to the national government (Goetz, 2008). Furthermore, at the legislative and political levels, equal rights that may be enjoyed by women and girls in national legislation are often not codified at the local or subnational level due to contravening customary law and social norms (UN Women, 2015).

In FCAS, these kinds of disadvantages and inequalities are greatly amplified (Speake, 2013). Violence and fragility affects women and girls in many ways, some of which differ from the impacts on men and boys. For example, sexual assault and exploitation of women are often used as “tools of war,” and particular threats, such as child marriage and human trafficking of women, are often exacerbated during conflicts (USAID, 2007). There are many drivers of the differences in effects of conflict on women and girls from men and boys, such as the implications of the different social roles assigned to men and women, which vary by context. Understood in this way, the particular effects of conflict on women do not stem from any intrinsic weakness in women, but are rather a consequence of their position in society (Pankhurst, 2000).

In addition to the immediate threats to women, conflicts can have a destabilising effect on gender norms in the long term. Gender roles can change during conflicts by increasing the burden and responsibilities of women while men are away fighting. Such shifts in gender and social norms can manifest as new sources of conflict when women are expected to return to pre‐conflict status (United States Institute for Peace, 2012).

At the root of this issue is the exclusion of women from the processes that foster peaceful and inclusive societies. A study of 31 major peace processes between 1992 and 2011 by UN Women found that only 2.4% of chief mediators, 4% of peace signatories, and 9% of negotiating delegations were women (O'Reilly, 2013). Between 2000 and 2010, less than a third of peace agreements signed contained a gender reference (Hedström & Senarathna, 2015). The exclusion of women's voices and contributions to peace processes not only leave women disenfranchised, underrepresented and vulnerable, but studies have shown that limiting women's participation is associated with greater recidivism and return to conflict (Bigio & Vogelsteing, 2016; Council on Foreign Relations, n.d.; Hedström & Senarathna, 2015; O'Driscoll, 2017).

In summary, there cannot be positive peace, or the creation of social systems and foundations for durable, peaceful, and inclusive societies, without an empowered female population that participates in the restoration and/or construction of relationships (Galtung, 1996).

### 1.4 A solution: Women's empowerment and gender equality in FCAS

Processes that develop peaceful societies are not only opportunities to strengthen, reconstruct or promote the resilience of social cohesion but also to transform the roles and definition of social relations towards inclusivity, participation and peace (Aall & Crocker, 2019). Rebuilding social cohesion and inclusive societies may be buttressed by women's empowerment and by the promotion of gender equality for peace and through peace (Björkdahl & Höglund, 2013). Protecting women and girls’ human rights, safety, physical and mental health and security, promotion of their socio‐economic recovery and increased participation in decision‐making processes and responses related to conflict or fragility are key processes that lead to overall progress towards gender equality and women's empowerment (Buvinic, Gupta, et al., 2013).

Empowering women across all three of the dimensions of gender equality and women's empowerment, according to Kabeer, often requires structural and systematic changes (Kabeer, 1999). Implementing transitions based on peace, nonviolence and inclusion can be driven by repealing old laws and instituting new structures, but must also transform harmful social norms that exclude women from publicly and meaningfully participating in society (McWilliams & Kilmurray, 2015). This includes ensuring that women have equal rights, are treated equally, and have their voices heard and needs met. Promoting the role of women and facilitating exchanges between them help change those mindsets and promote greater gender equality, reduce conflict, and discourage extremism. In that sense, women's empowerment can both be a driver and a consequence of peace: empowerment *through* and *from* peace (Cheldelin & Mutisi, 2016).

### 1.5 UNSCR 1325 as the starting point of change towards inclusive peace

When the United Nations Security Council Resolution (UNSCR) 1325 (United Nations Security Council, 2000) was adopted on 31 October 2000, it represented a major milestone in acknowledging the disproportionate impact of armed conflict on women and girls in continuation of previous work started in the 20th century. The resolution, which has been used extensively as a policy tool to implement gender‐sensitive conflict‐related policies, affirms three major points:

I. The recognition of the inordinate impact of violent conflicts and war on women and girls;
II. The recognition of the crucial role that women should play in conflict prevention, resolutions, building peaceful and inclusive societies; and
III. The necessary adoption of a gender perspective in conflict prevention, resolutions, and building peaceful and inclusive societies.

UNSCR 1325 has widely been interpreted as asserting those objectives by establishing four pillars of women's roles in conflict: participation, prevention, protection and relief and recovery through gender equality and for gender equality.

Although the UNSCR 1325 prioritised women's empowerment for peace and initiated the Women Peace and Security Agenda (WPS), some criticisms have been raised (Senarathna, 2015). First, women are not an homogeneous group that can be represented as an entity in peace processes. Gender is both multidimensional and intersectional and thus a *one‐size‐fits‐all* approach may not be appropriate. Second, the formulation of *women as agents of peace* has been criticised as being *instrumentalist*, focusing on what women can bring to peace and not what peace can bring to women. Finally, women are not only exposed to violence in periods of conflict, but may also be highly exposed to violence in times of peace and both should be considered in the WPS. Despite these critiques, the UNSCR 1325 has been the starting point of 20 years of change towards inclusive peace as detailed in the table below (Desmidt & Davis, 2019). See Appendix H, Table H2 for a more complete chronology of the impact of UNSCR 1325.

UNSCR 1325 has initiated a global movement towards consensus on the importance of the role of women in building peaceful and inclusive societies, but concrete implementation of the recommendations remains scattered. In 2018, out of 52 agreements across a range of issues for peace, only four contained gender‐related provisions (UN Women, 2020). In 2019, only 41% of the UN Member States had adopted a national action plan (NAP) for the UNSCR 1325 (Desmidt & Davis, 2019). Though it is still early to evaluate its impact, the novel coronavirus (COVID‐19) has and will continue to aggravate existing intersectional risks, strain the coping capacities of those most vulnerable, increase marginalisation and it is quite likely that these will be variables to take into account in current or ongoing peace processes. This SR will operationalise UNSCR 1325 by incorporating its four pillars into our framework to understand and classify intervention groups.

## 2 THE INTERVENTION

### 2.1 Conceptual scope of the review

A wide breadth of different interventions are currently being implemented to help empower women in FCAS contexts. As per the figure below, to structure our review of this broad range of interventions and to specify the types of intervention we will include, we have developed a guiding framework to:

1. **Include only those intervention groups that are either** ***gender specific*** **or *gender transformative***, as guided by the World Health Organization's *Gender Responsiveness Assessment Scale* (World Health Organisation, n.d.). Gender Specific interventions are those that consider gender norms, roles and relations for women and men and how they affect access to and control over resources. They intentionally target and benefit a specific group of women or men to achieve certain policy or programme goals or meet certain needs. Gender Transformative interventions are those that address the causes of gender‐based inequities and include ways to transform harmful gender norms, roles and relations to foster progressive changes in power relationships between women and men. Given that our review aims to identify the effectiveness of interventions whose specific and explicit aim is to empower women in FCAS, we will use this device as a filter to exclude studies which may include a disaggregation for sex in their study design, but for which the intervention was more broadly targeted. As a result, we are considering intervention groups of *all* genders and ages, but those that have an explicit focus on gender. This includes any focus on LGBTQIA+ groups to represent sexual and gender diversity.
2. **Include only those intervention groups that operate within the micro‐ (individual) and meso‐ (household and community) spheres of the ecological framework**, that is to say, not those which operate at the macro (or society) level. As is outlined by Bronfenbrenner (1979), we recognise that an individual's immediate setting “…is affected by the larger context in which the settings are embedded”. That said, our decision to focus on the lower levels of the ecological framework is driven by this review's focus on those interventions which work directly with individuals and their communities.
3. **Classify interventions based on the four pillars of UNSCR 1325**. While some interventions work across multiple pillars, we have decided to operationalise this widely used framework to help organise our findings. In doing so, our review will be in line with national, international, and multilateral development priorities and the evidence that we present will be easily navigable and applicable for policy makers and programme managers (Table 1).

**Table 1:** Conceptual scope of the systematic review

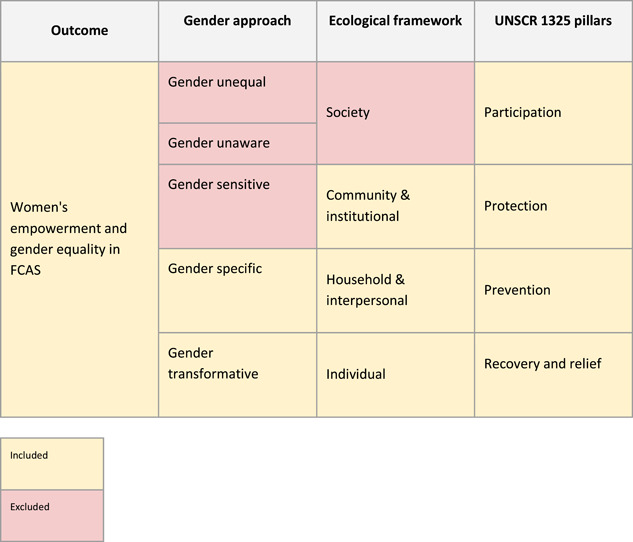

These pillars of the UNSCR 1325 are large beyond the possible scope of this review, and not all interventions within them will be included. Further detail follows in Summary of Included Interventions.

### 2.2 Summary of included interventions

**Figure 1:**
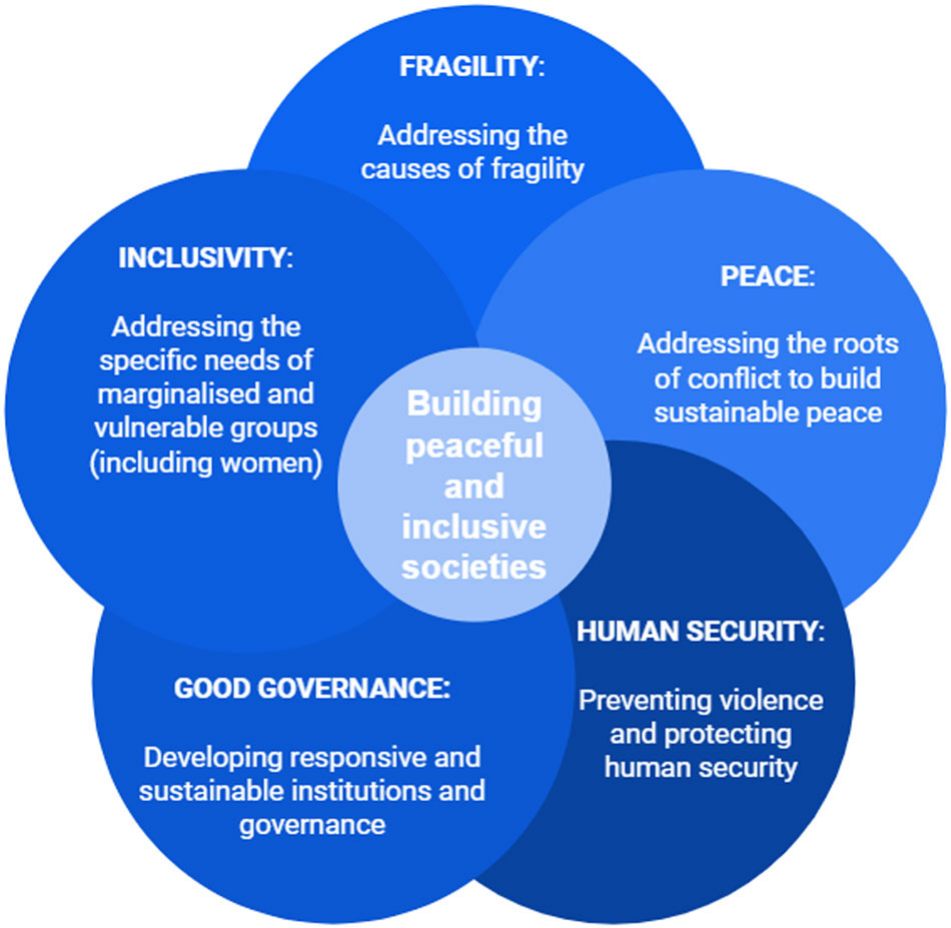
Building peaceful and inclusive societies

The scope of efforts which aim to empower women in FCAS is vast, and likewise, the body of impact evaluations significant. In Figure 1, we operationalise our conceptual scope as a tool to help identify specific categories of interventions to include in this SR. As an extension of that, the following are descriptions of the categories of interventions we intend to include within each of the four UNSCR pillars.

#### 2.2.1 Participation

For the purpose of our review, we define participation interventions as those that create opportunities for, build acceptance of, or strengthen capacities for the equal participation and full involvement of women and girls in political, economic and social institutions and decision‐making processes (Table 2).

**Table 2:** Participation pillar, included studies

Intervention	Description
Civil society, associations and networks	Interventions that aim to strengthen the technical capacity and institutionalisation of CSOs, governments and market driven‐associations working for gender equality or women's empowerment. This also includes the establishment and support of women's economic associations, such as cooperatives and activity groups
Economic rights and entitlements	Interventions that protect or support women's access to land rights and entitlements such as unemployment benefits
Formal education	System, school, and teacher‐level interventions that promote gender equality, girls' access to and the gender sensitivity of formal education, and/or develop human capital amongst girls and women through school‐based education. This can also include education financing and planning interventions like vouchers for girls to attend low‐fee private schools
Non‐formal education	Access to education and educational resources for women and girls that occurs outside of traditional schools including community‐based education, camp‐based education, and so forth
TVET	Education for women and girls that provides knowledge and skills useful for employment, jobs placement in the formal sector
MSMEs	Support for women‐run micro, small and medium enterprises. This may include incubation‐style interventions, loans, technical support, entrepreneurship training, mentorship or start‐up kits
Cash‐based approaches to support women's access to and participation in education and/or the economy	Cash‐based interventions which aim to develop human capital (e.g., enable girls' access to education) or empower women to participate in the economy
Financial inclusion	Interventions that provide access to credit, savings, insurance and other financial products, either on an individual or group‐based approach. This includes self‐help groups, village savings and loan associations (VSLAs), savings groups, and so forth
Community and leisure activities	Interventions that create opportunities or encourage support for women's participation in community and leisure activities, such as sports, art, theatre, and so forth
Civic education and youth leadership	Interventions that build capacity and create opportunities for women and girls to understand and participate in politics and political processes. This also includes support to girls and young women to learn skills for and access mentorship and opportunities to become active members of their communities
Voice and participation in local and subnational governance and development bodies	Interventions which aim to increase the voice and participation of women and girls' in local government through quotas, outreach and encouragement campaigns, and so forth. This also includes efforts to strengthen the responsiveness of local and subnational governance and development bodies to women's needs and priorities. Finally, it includes interventions that provide training or opportunities enabling women and girls to hold public services and government accountable for service provision and protection of their rights

#### 2.2.2 Prevention

For the purpose of our review, we define prevention pillar interventions as those that build capacities and systems to support the gender‐responsiveness and inclusivity of violence prevention and conflict transformation processes. This also includes efforts to hold perpetrators of violence accountable through formal or informal means (Table 3).

**Table 3:** Prevention pillar, included studies

Intervention	Description
Conflict early warning systems	Interventions that facilitate women's involvement in, or the gender‐inclusiveness or gender‐responsiveness of community‐based or online conflict early warning systems
Dialogue groups	Interventions that either facilitate women's involvement in, or set up women‐specific community dialogue groups. These can be forums that draw participants across the community to exchange ideas in face‐to‐face moderated sessions, share personal stories and experiences, express perspectives, clarify viewpoints, and develop solutions to problems. These may include community conflict prevention fora, dialogues to resolve or transform specific conflicts, or dialogues to build social cohesion
Community consultations	This includes interventions that create specific consultations for women at community and subnational levels as part of a formal peace process
Capacity building for conflict transformation	Interventions that build women's capacity to participate in or the gender‐sensitivity of community and subnational conflict transformation processes. These may also be referred to as building skills for mediation, negotiation, conflict resolution or conflict prevention
Peace education	This includes interventions that aim to teach people about the importance of including women in peace processes

#### 2.2.3 Protection

For the purpose of our review, we define the protection pillar interventions as those that create, facilitate access to, or build awareness of and support for legal or social protections for women's and girls' rights. This also includes behavioural, legal and environmental interventions that aim to reduce women and girls' risk of experiencing SGBV (Table 4).

**Table 4:** Protection pillar, included studies

Intervention	Description
Legal rights education	This includes interventions that disseminate information and build understanding of women's rights, among men and women, and capacity‐building on ways to demand their rights
Behaviour change communication around support for women's rights and preventing SGBV	Community‐based and subnational‐level interventions that aim to change behaviours, attitudes and beliefs around gender equality and the role of women in society, as well as efforts to reduce the prevalence of SGBV. This could include the targeting of gender norms within communities through social institutions, such as churches, community groups or cooperatives. It could also include efforts to sensitise families on the negative consequences of SGBV, particularly for children. These interventions may target local community and civil society leaders, local and subnational government, the private sector, and so forth. This would also include media interventions such as radio dramas or participatory theatre aiming to encourage discourse around women's rights and empowerment
Capacity building and technical support to subnational government officials to strengthen service provision for women and gender equality	These interventions build the capacity of subnational and local government officials to provide services for women, meet women's needs, and improve understanding of women's rights and the importance of gender equality. This may target gender equality‐specific officials such as officials within a Department of Women's Affairs, or aim to strengthen the capacity and service provision for women within sector‐specific services such as education officials
Preventative protection measures	These interventions comprise non‐police or security force‐based efforts to reduce incidences of violence, especially SGBV. They include making the physical environment less conducive to such acts and minimising the exposure of vulnerable groups to risky situations. This can include crime prevention through environmental design intervention, installing lighting in public spaces, women‐friendly transport systems and public facilities, removing obstacles so there is a better line of sight and reclaiming spaces for positive community activities. They may also include interventions to reduce vulnerable groups’ risk of exposure to violence (e.g., through providing firewood or alternative fuel sources to women in refugee camps)
Gender‐sensitive policing	Interventions that strengthen the approaches and capacity of police forces to prevent SGBV, improve police support to and treatment of women and girls, and support victims in a respectful way that does no harm. This includes, for example, training and capacity building interventions targeting police forces that strengthen their awareness of the importance of gender equality and women's legal rights, train them on best practices for protecting victims, and interventions that create women‐specific police officers to facilitate access to reporting crimes
Informal judicial system	Interventions supporting women's access to or participation in informal or semi‐formal justice processes, such as alternative dispute resolution mechanisms, village courts

#### 2.2.4 Relief and Recovery

For the purpose of our review, we define relief and recovery interventions as those that build capacities and systems to support the gender‐responsiveness and inclusivity of relief and recovery processes related to conflict, displacement and natural disasters (Table 5).

**Table 5:** Recovery and relief pillar, included studies

Intervention	Description
(Re)integration of forcibly displaced populations	This includes gender specific or transformative interventions that target forcibly displaced populations, with the aim of supporting (re)integration into host or home communities. This may include economic support such as cash‐based approaches (cash transfers, vouchers, etc.); social reintegration such as through the organisation of social activities or sports leagues; and community development inclusion such as strengthening the inclusion of displaced women in community driven development interventions
Disaster risk reduction	Interventions to support women's participation in or the gender responsiveness of community‐based disaster risk reduction activities (such as hazard vulnerability and risk assessments, etc.)
Disarmament, demobilisation and reintegration	These interventions support forcibly displaced populations to (re)integrate into host or home communities. This may include economic support such as cash‐based approaches (cash transfers, vouchers, etc.); social reintegration such as through the organisation of social activities or sports leagues; and community development inclusion such as strengthening the inclusion of displaced women in community driven development interventions

### 2.3 How the intervention might work

Our framework acknowledges both the wider and more specific contexts within which gender specific and transformative interventions are implemented in FCAS. Interventions operate within a wide context of international standards and practices, including the UNSCR 1325, the 2030 SDGs or the WPS, and this global context overlays the unique local context of any given intervention where those general standards are applied. The framework captures the reality that while broader principles often guide intervention design, the characteristics of fragility and conflict depend on the location, motivations, interactions and local specificities of the intervention fields.

**Figure 2:**
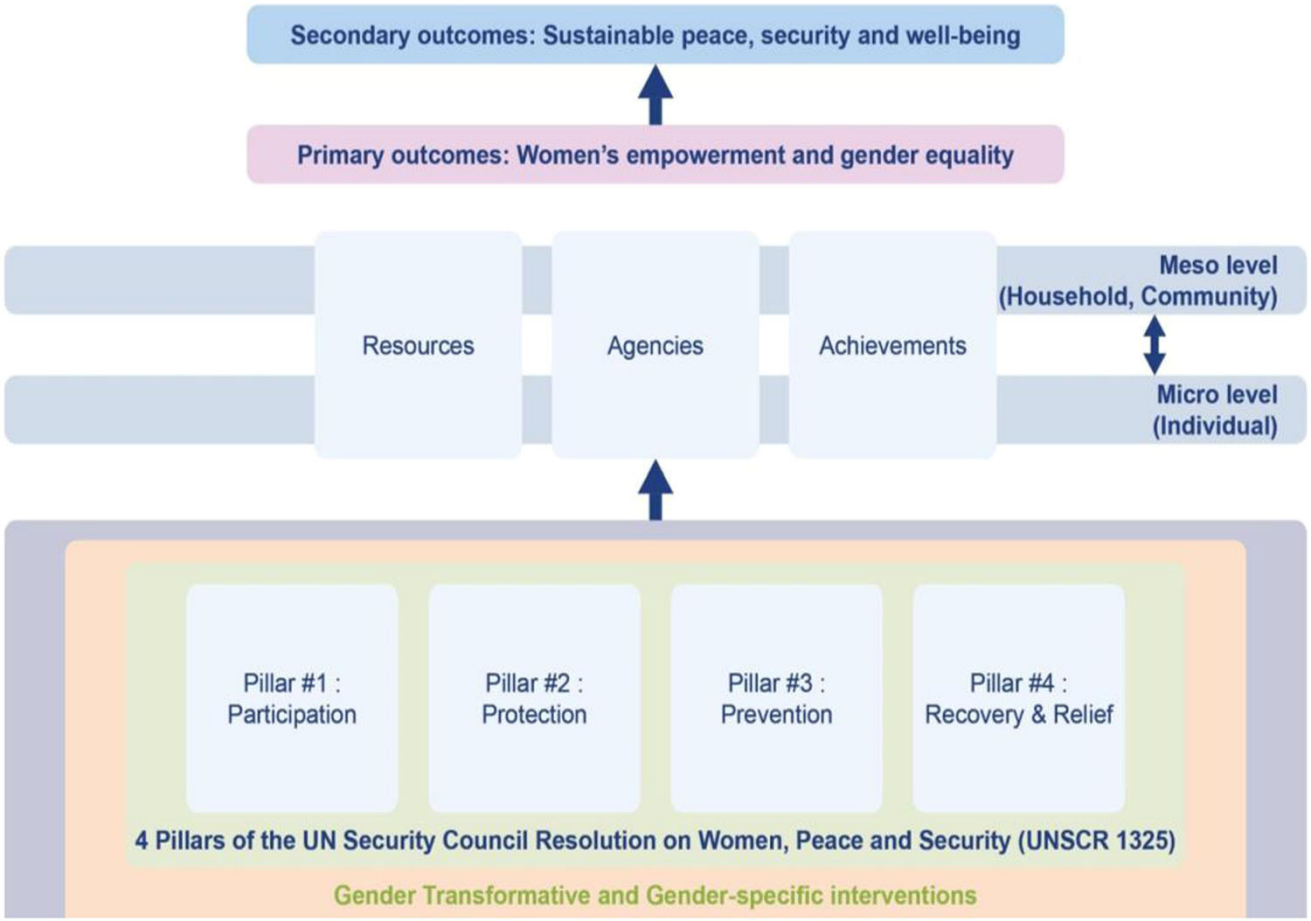
Our systematic review's theory of change

As per the conceptual scope, the below Theory of Change (Figure 2) includes only those interventions operating in the micro and meso levels of the ecological framework and those that are gender specific or transformative‐‐those that work to develop the resources, agency and achievement levers of women's empowerment and gender equality.

Importantly, the UNSCR Pillars are not necessarily mutually exclusive. Interventions can work across multiple pillars, levels and levers with the common objective to impact women's empowerment and gender equality. Lastly, the primary outcome of the review is women's empowerment and gender equality that will then contribute to achieving the secondary outcome of peaceful and inclusive societies.

### 2.4 Why it is important to do this review

#### 2.4.1 Review of existing literature

##### 2.4.1.1 The existing literature

3ie's recent EGM, *Building peaceful societies*, demonstrated that a considerable body of literature exists regarding the impact of interventions that focus on women and girls in FCAS. In particular, there is a wide scope of experimental and quasi‐experimental primary research focusing on peaceful and inclusive societies and women's empowerment. As is outlined above, much of this evidence suggests a link between the integration of gender‐sensitive approaches in FCAS context and lasting achievements in building, ensuring human rights, and promoting inclusive societies. That said only a handful of SRs actually focused on the role of women in peace processes and there is a stronger emphasis on empowerment of women in fragile contexts for sustainable peace.

After completing a comprehensive scoping of existing SRs and impact evaluations (IE) through the 3ie Evidence Portal, we identified seven IEs and two EGMs focusing on the topics of building peaceful societies and fragility. 3ie notably contributed to the production of the two evidence gaps maps focusing on *Building peaceful societies* (Sonnenfeld et al., 2020) as an update and expansion of the 2015 EGM on *Evidence for peacebuilding* (Cameron et al., 2015). The *Evidence for peacebuilding* EGM identified over 100 IE, four SR and two protocols. The *Building peaceful societies* EGM identified about 250 IEs, 34 SRs and four protocols.

Women's empowerment in fragile contexts has been substantially reviewed. A search of 3ie's Development Evidence Portal identified 39 impact evaluations, 16 SRs, and one EGM focusing on topics of women and girls empowerment, including contexts in FCAS.^1^ As identified through the focus of the EGM (Picon et al., 2017), a majority of the reviews with an explicit and specific focus on women in FCAS focus on the prevention of and recovery from intimate partner violence (IPV).

There is also a substantial literature of SRs and IEs integrated within the second pillar of our framework (prevention) with a high prevalence of IEs focusing on the incidence or reaction to IPV with 62 out of 153 identified IEs. SGBV and IPV are also the main focus of five SRs and two protocols (Delkhosh et al., 2017; Signorelli et al., 2018).

The Spangaro‐led review (Spangaro, Adogu, et al., 2013) notably found that although guidelines and training courses have been developed at the policy level, few initiatives have been implemented and evidence is lacking to judge the effectiveness of interventionsdesigned to address or prevent sexual violence in conflict‐ or crisis‐affected settings. IPV has also been the focus of a SR *of structural interventions for IPV in L&MICs* (Bourey et al., 2015) *and in humanitarian crises* (Warren et al., 2015). Some reviews also focused on the recovery and health aspects of IPV (Rivas et al., 2015; Tol et al., 2013).

Our literature scoping also identified a cluster of studies focusing on women's economic empowerment. Reviews of economic empowerment interventions include community‐based programmes such as economic self‐help groups where some reviews identified positive effects on women's political and economic empowerment (Brody et al., 2017). Others focus on the role of economic resources transfer for women's empowerment (Kabeer & Waddington, 2015). A third type of economic empowerment focuses on the impacts of business oriented programmes such as business and vocational training to improve women's labour market outcomes that tends to have more impact when they are combined with cash transfer or life skills training (Balarin et al., 2017).

A number of reviews include a specific focus on livelihoods (Gibbs et al., 2012) at the community level to complement reviews integrating a health lens at the individual level (Kraft et al., 2014). Such characteristics of the literature landscape confirms the relevance of analysing women's empowerment through an ecological framework.

The political dimension of empowerment is also highly reliant on the community level of the ecological framework through the mobilisation of civil society groups to serve empowerment purposes notably in the subcategory of reproductive health for marginalised groups (Handanagic et al., 2016; Moore et al., 2014). Although we have been able to then find numerous references to gender empowerment in SRs, none of them have a specific focus on gender empowerment for and through building peaceful and inclusive societies processes in FCAS.

Additionally, though women's empowerment in fragile contexts is at the core of our review, it is important not to forget the wider spectrum of governance and social protection themes integrated in this review. The 3ie Evidence Portal gathers over 170 impact evaluations, 11 SRs and two evidence gap maps, including one in‐progress, covering issues of governance. Themes such as accountability, transparency, management of resources, public administration are all relevant aspects for the analysis of fragile and conflict‐affected situations as they are both sources of conflict and opportunities for peace. The two EGMs notably focused on the State‐Society relations (Coffey et al., 2017) (18 SRs, two protocols and 365 impact evaluations) with a major focus on public institutions and services and on the effect of rule of law interventions on justice outcomes (Doherty et al., 2020).

##### 2.4.1.2 Our contribution to the existing literature

The above summary of findings from existing reviews reveals that although both fragility and women's empowerment are key areas of focus of systematic research, only a handful of studies focus on women's empowerment in FCAS and/or gendered approaches to address fragility. Even when they do there usually is only particular emphasis on the impact of fragility on women rather than women's roles in shaping peace.

This initial summary also confirms the relevance of the conceptual scope of our review as we saw that a number of reviews focused on some of its aspects, including a specific attention to gender transformative and gender specific IE in the SRs covering women empowerment that matches our approach. Many of the SRs focusing on these aspects actually cover it through the lens of one of the levels of the ecological model with a prevalence of the reviews focusing on the community level. Finally gender and peacebuilding‐focused SRs overlap on one or several of the 1325 pillars (participation, prevention, protection, recovery).

These gaps identified in the systematic literature should not be interpreted as a lack of interest for women's empowerment for peace and inclusivity. This literature review has allowed us to identify a number of impact evaluations already covering relevant aspects for our study. The gaps of literature are then mainly due to the challenging analytical aspects that this topic raises. The high amount of non‐systematic literature covered through the scoping stages of our research is a good illustration of the interest of these topics for researchers. This review will contribute to the SR literature through the development of understanding of the causal impacts of gender sensitive and gender transformative interventions in FCAS.

#### 2.4.2 Relevance to policy and practice

##### 2.4.2.1 Relevance to international and national development goals

An objective of our study will be to feed into some of the international goals and approaches supporting women empowerment and gender equality in fragile contexts. Internationally, the interest in gender equality and its relationship to peaceful societies can be measured in the amount of investments being made in both the SDGs relevant to gender equality and fragility, and national and regional‐level initiatives. An estimated $1 trillion USD would be necessary to meet SDGs #5 on gender equality and #16 on peaceful and inclusion societies (Peace Women, 2016). There is growing recognition that investment in military spending is not the only solution for peace, but that solution can also come through an increased investment and recognition of women's rights and gender equality worldwide. Some international mechanisms have been created, such as the Addis Ababa Action Plan on Transformative Financing for gender equality and women's empowerment (Peace Women, n.d.) and the Global Acceleration Instrument (GAI) for Women, Peace and Security and Humanitarian Action, which represent pooled, transnational sources of funding for gender equality and women's empowerment.

At the national level, donors and countries have taken some measures to target women's empowerment and gender equality in FCAS since 1325 was introduced but more generally in the support of achievement of international development and growth. The German international development agency, GIZ, published its vision for promoting gender equality as one of the key values of its approaches abroad and as a prerequisite and driver of sustainable development (Langenkamp, n.d.). Global Affairs Canada (GAC) updated its international assistance policy in 2020 to include a feminist perspective. The updated policy recognises the importance of supporting gender equality and the empowerment of women and girls as it is the best way to build a more peaceful, more inclusive and more prosperous world (Global Affairs Canada, 2017). The UK's former Department for International Development (DFID), which is now the Foreign, Commonwealth, and Development Office (FCDO), published a Strategic Vision for gender equality in 2018. The document is presented as a call to action, recognising that action is needed if gender equality is to become a lasting reality (Department for International Development, n.d.‐c). The Ministry of Foreign Affairs of the Kingdom of the Netherlands publishes reports every 4 years to focus its impact on UNSCR 1325 and the Women, Peace and Security Agenda (Ministry of Foreign Affairs, n.d.).

##### 2.4.2.2 Relevance for donors and intervention design

Donors and implementing agencies have led interesting and innovative interventions in FCAS to increase the role of women in fostering peaceful societies:

The German Ministry for Economic Cooperation and Development (BMZ) have implemented a project in conjunction with the African Union (AU), entitled “Support to the African Peace and Security Architecture” (APSA), to work with regional organisations and other international stakeholders to improve the participation of women in activities in the peace and security sector. BMZ has also supported the operationalisation of the AU‐supported Network of African Women in Conflict Prevention and Mediation (*FemWise‐Africa Network*), which collaborates with civil society to build the mediation skills of young women and bridge intergenerational gaps. The project seeks to promote the role of women in preventive diplomacy and mediation at all levels, ensure channels for their meaningful participation, initiate their action in line with the SDGs, bridging the gap between different levels of mediation and establishing local and national peace infrastructure for long‐term initiatives.

BMZ also supports compliance training addressing fundamental human rights and zero tolerance for sexual abuse and exploitation (SEA). More recently, BMZ has offered support to the implementation of the regional stabilisation strategy (RSS) for the Lake Chad Basin region, mental health support in Boko Haram‐affected regions and hosted regional workshops on SGBV.

Since 2011, the *Red Nacional de Mujeres* has published an annual report on monitoring the progress of the implementation of UNSCR 1325 (Red nacional de mujeres, n.d.), which has been in conjunction with the country's NAP on 1325. *Ruta Pacífica de las Mujeres*, an organisation that was key in advocating for the inclusion of women's voices in the 2016 Colombian Peace Treaty, includes a list of indicators across all levels of the ecological framework in their strategic plan to monitor progress on women's empowerment in the Colombian context (Ruta pacífica de las mujeres, 2016).

In Pakistan, the FCDO funds the Voice and Accountability programme (AAWAZ), which brings together three critical components focusing on the interconnection of gender, conflict resolution and citizen engagement (Department for International Development, n.d.‐a). This strives to focus on the enhanced political participation of women and in larger public life without fear of sexual or gender‐based violence, while striving to attain social harmony. This is set against the backdrop of promoting civil society, by having active and informed participation of citizens and their organised groups. More broadly, the FCDO also funds the Global Challenge Research Fund (GCRF) Gender, Justice and Security Hub (Department for International Development, n.d.‐b). On a higher level, the Hub functions to deliver ambitious and impactful research facilitating gender justice and inclusive security in FCAS.

The Dutch Ministry of Foreign Affairs funds, through its SDG5 Fund, a variety of modalities, including Women, Peace and Security, the Power of Women fund and grants for protecting women and girls sexual and reproductive health rights (SRHR). Consequently, the overall objective of the strategic WPS‐partnerships is to contribute to an enabling environment for women's participation and empowerment in conflict and post‐conflict environments, so they can meaningfully participate in conflict prevention, resolution, fostering peaceful societies, protection, relief and recovery.

##### 2.4.2.3 Relevance for policy design and decision making

Although it is still an ongoing process, we can already see some positive impacts of the WPS agenda. Data notably shows an increase in bilateral aid in support of gender equality and women's empowerment in FCAS (UN Women, n.d.) from some of the key states of funding of international development programmes. According to UN Women, all top bilateral donors increased their proportion of spending in support of gender equality and women's empowerment in FCAS over the last decade alone. For example, the United States gave approximately $2 billion USD in 2010, yet increased that number still to nearly $3 billion in 2017. However, we see that the proportion allocated by these same countries to bilateral aid dedicated to gender equality and women's empowerment as a principal objective in FCAS is lower than 6% of aid and has decreased over the last decade.

The adoption of National Action Plans (NAP) by individual countries to adopt the WPS Agenda as outlined in UNSCR 1325 has some encouraging results in Central and West Africa and Latin America but is still an ongoing process in Southeast Asia and in the MENA region and we actually see a relatively low rate of adoption of NAP in the medium to high level of violent conflict regions (UN Women, n.d.). Some examples of NAP are briefly presented below (London School of Economics, 2019). See Appendix I for some examples of National Plan for Action on UNSCR 1325.

Since the first NAP was pioneered in 2005 by Denmark, over 80 countries have piloted their own NAPs focusing on the WPS agenda (Peace Women, 2014). However, despite there being nearly 84 NAPs (as of December 2019), very few (33%) contain an allocated implementation budget. Additionally, only 31% have references to disarmament. Lastly, while the majority of NAPs mention the role of grassroots leaders and civil society (75%), most allocate them to an advisory role. There are 11 additional regional action plans (RAPs), such as for regional bodies like the AU and EU. Regional coordination efforts also include the Asia‐Pacific Regional Symposium on National Action Plans on Women, Peace and Security where the Member States, alongside civil society representatives, share their lessons learned and best practices in the implementation of UNSCR 1325.

Twenty years after the introduction of UNSCR 1325, the purpose of NAPs and their impact has yet to be fully explored. The dynamic nature of conflict and the intersections of gender often mean that perspectives are constantly shifted and not always accurately captured in plans or data (Hamilton et al., 2020). Additionally, without implementation plans or budgets, NAPs ultimately end up being solely documents that do not move past rhetoric towards commitment. These trends illustrate the challenges raised to increase the role of women in building peaceful and inclusive societies. Our review will then help inform decisions about how to spend the resources available by providing a comprehensive review of the evidence on the impact of women as agents of change to build peaceful and inclusive societies in FCAS.

## 3 OBJECTIVES

This review builds on 3ie's EGM of the impact evaluation and SR evidence base of interventions aiming to promote peaceful and inclusive societies in fragile contexts (Sonnenfeld et al., 2020). The EGM identified a cluster of studies evaluating gender equality‐focused behaviour change communication programmes and raised interest in investigating the evidence base for understanding the role of women more broadly as agents of change in developing peaceful and inclusive societies.

Building on the cluster of evidence identified in the EGM, our review will increase generalisability of findings from single studies and focus on interventions across a broad range of geographical locations, settings and populations, types of implementations and outcomes. We will also address (when possible) the identified gaps in literature regarding meta‐analysis in conflict‐affected contexts.

As such, we propose the following objectives:

1. The primary objective of this review is to identify, assess and synthesise evidence on the effect of gender specific and gender transformative interventions within the context of the four pillars of UNSCR 1325 on women's empowerment and gender equality in FCAS. The SR will facilitate the use of evidence in informing policy and practice decisions within the field of transition aid, particularly as it relates to gender focused programming.
2. Our second objective is to assess how these interventions contribute to inclusive and sustainable peace in conflict affected situations. We will compare the effectiveness of these different types of interventions through the lenses of their ecological level, types of impact on women's empowerment, local context of gender inequality and conflict.

To achieve these objectives we aim to answer the following questions:

1. What are the impacts of gender transformative and specific interventions on women's empowerment and gender equality in FCAS?
2. What are the effects of these interventions on sustainable peace?
3. To what extent do effects vary by population group, ecological level and types of interventions?
4. What are contextual barriers to and facilitators of intervention effectiveness?

## 4 METHODS

The review will follow the Campbell and Cochrane Collaborations approaches to systematic reviewing (Cochrane Collaboration, n.d.; Hammerstrøm et al., 2010; Shemilt et al., 2013). The review will also draw on the concepts of theory‐based impact evaluation (White, 2009) and theory‐based SRs (Snilstveit, 2012) to provide a mixed‐methods SR and analysis along the causal chain, reviewing evidence on context, process and implementation to identify barriers and facilitators and analyse the effectiveness of interventions aiming to improve women's empowerment and gender equality outcomes.

The review will systematically collect and synthesise quantitative evidence from impact evaluations meeting our inclusion criteria to answer our review questions. If sufficient data is available, outcomes will be synthesised in subgroups of populations, types of interventions, and local contexts. For the review to be more useful for policy‐makers and practitioners, we will also collect qualitative evidence from the included studies to assess factors that determine or hinder the effectiveness of interventions using a combination of qualitative synthesis and meta‐regression analysis.

**Figure 3:**
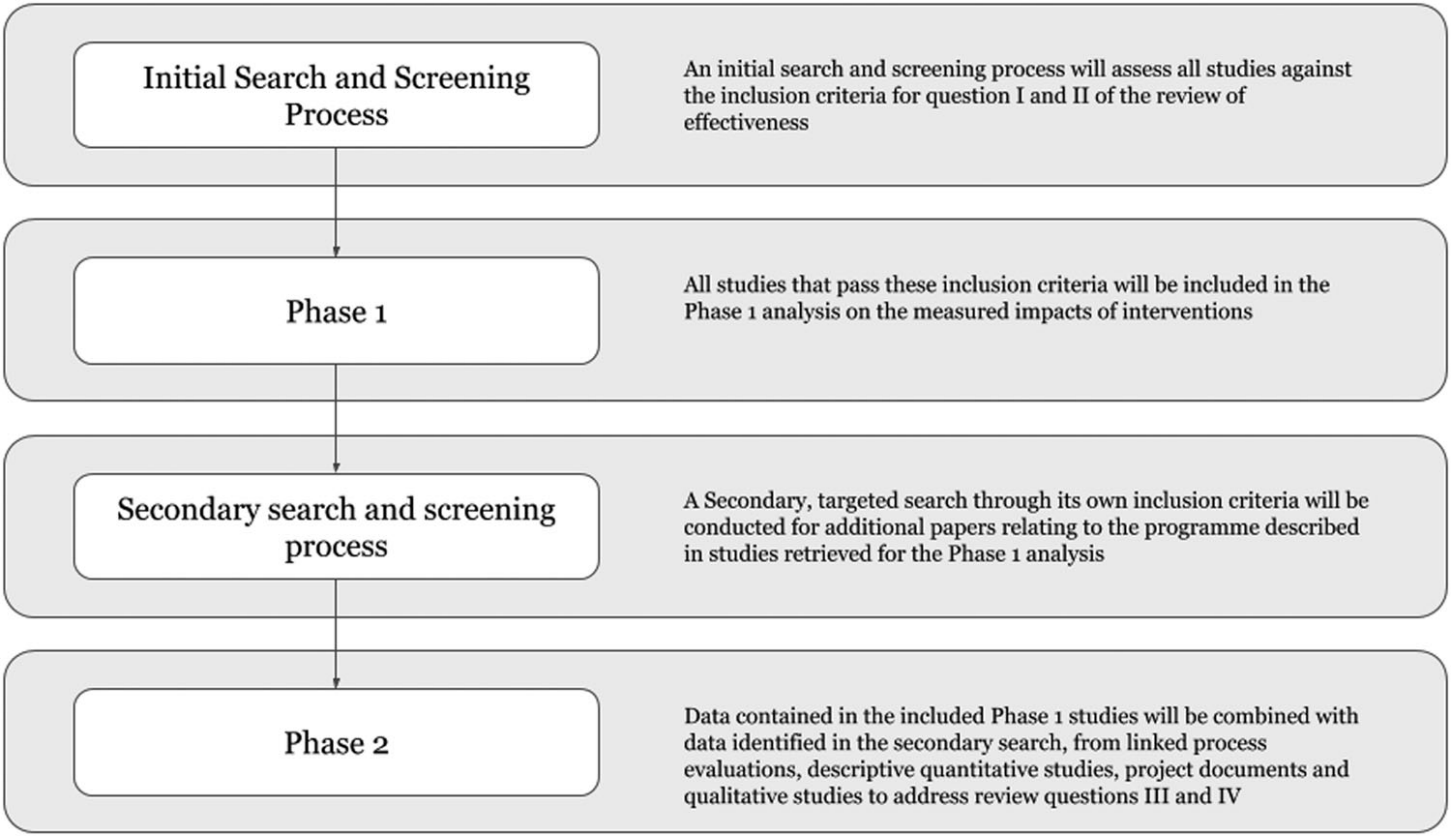
Overview of the review process

The review will include studies in two phases as per the figure below. To address the questions I and II, we will include studies meeting the impact evaluation study design inclusion criteria. To address the questions III and IV, IE studies that pass these criteria will then be used as the basis for a second phase to identify and include qualitative studies, project documents, and process evaluations (Figures 3).

### 4.1 Criteria for considering studies for this review

#### 4.1.1 Types of study design

We will include studies in two stages, in a similar approach to Snilstveit et al. (2015). In the first stage, we will include studies that assessed the effects of interventions using experimental designs or quasi‐experimental designs with non‐random assignment that allow for causal inference (to address primary research objectives 1 and 2). Specifically we will include the following:

- Randomised controlled trials (RCTs), with assignment at individual, household, community or other cluster level, and quasi‐RCTs using prospective methods of assignment such as alternation.
- Nonrandomised studies with selection on unobservables:
  ○ Regression discontinuity designs, where assignment is done on a threshold measured at pre‐test, and the study uses prospective or retrospective approaches of analysis to control for unobservable confounding.
  ○ Studies using design or methods to control for unobservable confounding, such as natural experiments with clearly defined intervention and comparison groups, which exploit natural randomness in implementation assignment by decision makers (e.g., public lottery) or random errors in implementation, and instrumental variables estimation.
- Nonrandomised studies with pre‐intervention and post‐intervention outcomes data in intervention and comparisons groups, where data are individual level panel or pseudo‐panels (repeated cross‐sections), which use the following methods to control for confounding:
  ○ Studies controlling for time‐invariant unobservable confounding, including difference‐in‐differences, or fixed‐ or random‐effects models with an interaction term between time and intervention for pre‐intervention and post‐intervention observations;
  ○ Studies assessing changes in trends in outcomes over a series of time points (interrupted time series, ITS), with or without contemporaneous comparison (controlled ITS), with sufficient observations to establish a trend and control for effects on outcomes due to factors other than the intervention (e.g., seasonality).
- Nonrandomised studies with control for observable confounding, including non‐parametric approaches (e.g., statistical matching, covariate matching, coarsened‐exact matching, propensity score matching) and parametric approaches (e.g., propensity‐weighted multiple regression analysis).

In a second stage, to address questions 3 and 4 on factors related to intervention design, implementation, and context, we will extract descriptive and qualitative data from the included experimental and quasi‐experimental studies. In addition, we will conduct a targeted search for additional papers on the interventions covered by the included impact evaluations to provide additional detail on these factors. In order to be included, these papers must be related to the intervention in the included impact evaluations and also be one or more of the following types of studies (Snilstveit, 2012):

- A *qualitative study* collecting primary data using mixed‐ methods or quantitative methods of data collection and analysis, and reporting some information on all of the following: the research question, procedures for collecting data, procedures for analysing data, and information on sampling and recruitment, including at least two sample characteristics.
- A *descriptive quantitative study* collecting primary data using quantitative methods of data collection and descriptive quantitative analysis and report some information on all of the following: the research question, procedures for collecting data, procedures for analysing data, and information on sampling and recruitment, including at least two sample characteristics.
- A *process evaluation* assessing whether an intervention is being implemented as intended and what is felt to be working more or less well, and why. Process evaluations may include the collection of qualitative and quantitative data from different stakeholders to cover subjective issues, such as perceptions of intervention success or more objective issues, such as how an intervention was operationalised. They might also be used to collect organisational information.
- A *project document* providing information about planned, ongoing or completed interventions. Such documents may describe the background and design of an intervention, or the resources available for a project. As such, these documents do not typically include much analysis of primary evidence, but they provide factual information about interventions. The purpose of including them in our review is to ensure we will have sufficient information about the context and interventions in included studies.

#### 4.1.2 Types of participants

We will include any participants from fragile communities in low‐ and middle‐income countries (L&MICs), including participants from the general population from all ages and genders and those from specific population subgroups, such as displaced populations, refugees, women, youth, and so forth. This wide definition of the population will then allow us to run subgroup analyses in our review such as rural and urban populations, socioeconomic groups or classes and castes, age, sex, and so forth.

##### 4.1.2.1 Challenges for the population definition

The definition of the population for this review raised a series of challenges to be considered:

- **The combination of women's empowerment and fragility criteria**: there is a gap in data available on both women's empowerment *and* fragile context. The team has run a scoping of indicators, indexes, and data available on women's empowerment in FCAS. The only index that covers both topics is the Women Peace and Security index designed by the University of Georgetown in 2017 but this index is not consensual in the research sphere as an indicator we can use on its own (Mundkur & Shepherd, 2018). Although this index is relevant to our SR its time frame is not close enough to our start date of intervention (2000) to be used as the only criteria for inclusion. This gap then requires the team to build its population criteria on the combination of existing indexes on gender *or* on FCAS.
- **The analysis of conflict affected situations at state**‐**level**: as presented in the previous sections, the study focuses on the micro‐ and meso‐ levels of the ecological framework. There are currently no indices that allow us to draw our population selection on community and local levels indicators of fragility. Data on fragility are generally available at the State level with indexes such as the OECD and World Bank FCAS lists, the Fragile State Index (FSI) and the Global Peace Index (GPI). This gap then requires the team to build its population criteria on State‐indicators although the analysis will focus on lower levels. While the core team attempted to extract data from available international datasets on femicide, homicide and other SGBV‐related indicators, the majority of datasets were incomplete, resulting in an inability to add any value to the analysis.
- **The potential size of the population**: the time extension and thematic focus would make almost all L&MICs eligible to be included in our SR. The resources available and timeframe would then not make it do‐able for the team and requires the team to set a series of thresholds on the identified indexes to build its inclusion criteria. This then means that some countries and regions have been prioritised for this study and that some excluded could have brought relevant studies for inclusion.

##### 4.1.2.2 Methodology of selection of the population

We created binary variables for whether a country was considered fragile or not, or gender unequal or not depending on the index, for each year for which data were available, using the thresholds noted above. We then created a series of consolidated lists for whether a country had appeared as fragile or unequal for at least 1 year during the period 2000–2020. We then grouped the countries into five sets, as follows:

- Set 1: There are 51 countries that appear on each of the World Bank, FSI, OECD and GII lists for at least 1 year.
- Set 2: There are 62 countries that appear on the GII and at least one of the fragility indices for at least 1 year (FSI, OECD or World Bank).
- Set 3: There are 23 countries that appear on the WPS list, but do not fall within Sets 1 or 2. This includes 10 countries that appear on the gender unequal GII list but none of the fragility indices, including contexts such as Brazil, El Salvador and India that were highlighted as potential relevant contexts during the AG call. It further includes three countries that appear on the FSI list but not the GII index. Finally, it also includes contexts such as Mexico that were identified as of interest by members of the Advisory Group (AG) due to high levels of violence.
- Set 4: There are 11 countries that have appeared on one of the fragility indices since 2000, but not the GII or WPS.
- Set 5: There is one country that appeared on the GII list for at least 1 year, but never on the WPS or other fragility lists (Argentina).

We developed two options for addressing the risk that including all countries within Set 3 at the title & abstract stage could create an unrealistic scope during full‐text screening:

- Set 3a: restrict the threshold for the WPS index to <0.6 instead of <0.7. This would shrink Set 3 to three countries (Benin, Gabon and India). However, it would exclude the potential contexts highlighted by the AG as of interest.
- Set 3b: include only the 10 countries that show up on both the WPS and the GII (Benin, Botswana, Brazil, El Salvador, Gabon, India, Morocco, Paraguay, Peru and Saudi Arabia). This would effectively treat the WPS as a similar fragility index to the ones included within Set 2. From the above‐noted countries, it would exclude Mexico, but it may be the most realistic approach that is nonetheless drawn on consistent criteria.

##### 4.1.2.3 Inclusion criteria at title and abstract screening (TAS) stage

Based on the challenges identified above and the preferred options for the sets 1,2 and 3b, we will include studies based on the following population criteria at TAS stage:

- L&MICs that are listed on all our indicators: in the World Bank list of FCAS (since 2006), on the OECD list of FCAS (since 2007), on the FSI with a score higher than 90 (since 2009) and that have a score of at least 0.6 on the GII for at least 1 year since 2000
- L&MICs that are listed in one of the fragility lists or had at least 1 year rated higher than 80 on FSI and that have a score of at least 0.6 on the GII for at least 1 year since 2000
- L&MICs that do not appear on the fragility lists but are listed with a score lower than 0.7 on the Women Peace Security Index between 2016 and 2019 and that have a score of at least 0.6 on the GII for at least 1 year since 2000

**Figure 4:**
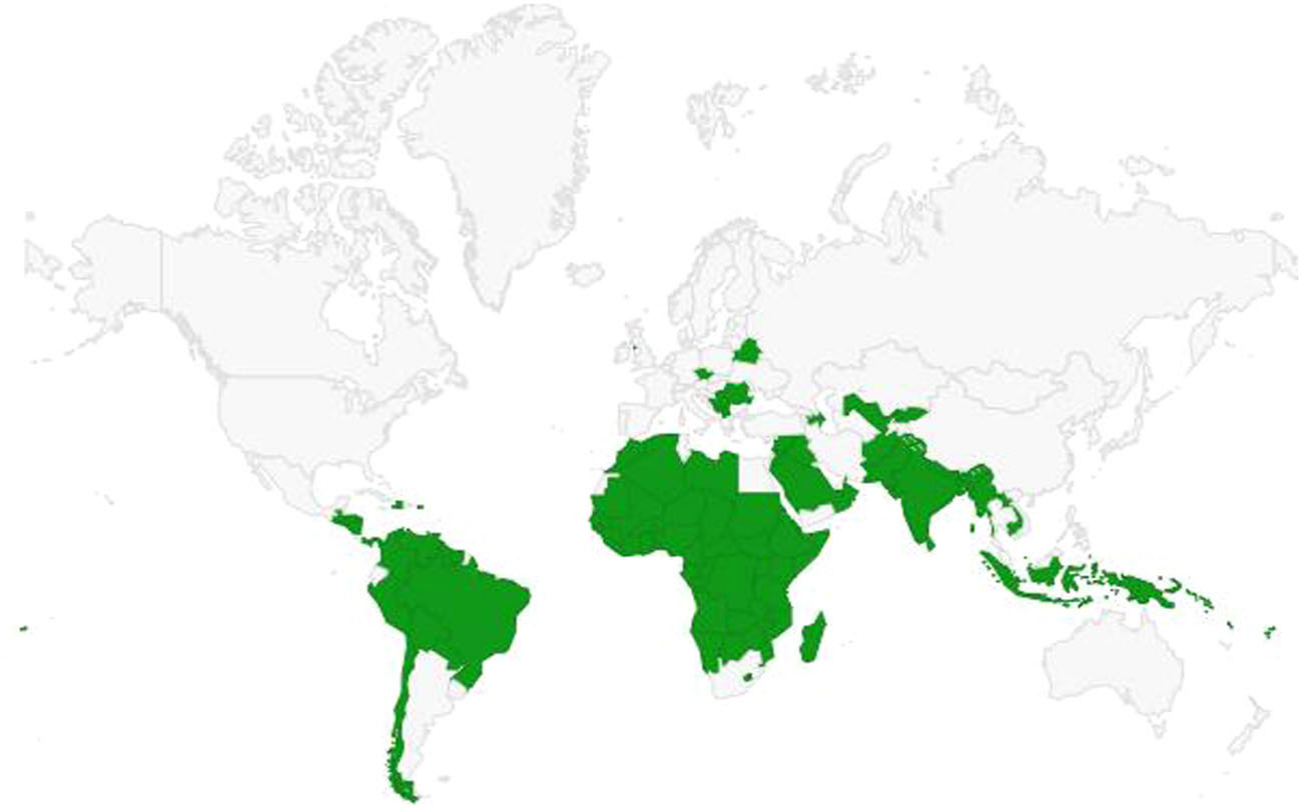
Map of includable countries for the systematic review. Light yellow is set 3b, yellow is set 2 and green is set 1

The above criteria allowed the team to build a list of included countries are shown in Table 6 and Figure 4.

**Table 6:** List of included countries for the systematic review

Set	Countries	
Set 1 There are 51 countries that appear on each of the World Bank, FSI, OECD and GII lists for at least 1 year	Afghanistan	Mali
	Angola	Marshall Islands
	Burkina Faso	Mauritania
	Burundi	Micronesia, Fed. Sts.
	Cambodia	Mozambique
	Cameroon	Myanmar
	Central African Republic	Nepal
	Chad	Niger
	Comoros	Nigeria
	Congo, Dem. Rep.	Papua New Guinea
	Congo, Rep.	São Tomé and Príncipe
	Côte d'Ivoire	Sierra Leone
	Djibouti	Solomon Islands
	Eritrea	Somalia
	Guinea	South Sudan
	Guinea‐Bissau	Sudan
	Haiti	Syrian Arab Republic
	Iraq	Timor‐Leste
	Kiribati	Tonga
	Kosovo	Tuvalu
	Lebanon	Uzbekistan
	Liberia	Vanuatu
	Libya	Venezuela, RB
	Madagascar	West Bank and Gaza
	Malawi	Yemen, Rep.
		Zimbabwe
Set 2 There are 62 countries that appear on the GII and at least one of the fragility indices for at least 1 year (FSI, OECD or World Bank)	Algeria	Korea, Dem. People's Rep.
	American Samoa	Kyrgyz Republic
	Antigua and Barbuda	Lao PDR
	Azerbaijan	Lesotho
	Bahrain	Maldives
	Bangladesh	Montenegro
	Belarus	Namibia
	Bhutan	Nauru
	Bolivia	Nicaragua
	Bosnia and Herzegovina	North Macedonia
	Cabo Verde	Northern Mariana Islands
	Chile	Oman
	Colombia	Pakistan
	Czech Republic	Palau
	Dominica	Panama
	Egypt, Arab Rep.	Puerto Rico
	Equatorial Guinea	Romania
	Eswatini	Rwanda
	Ethiopia	Senegal
	Fiji	Serbia
	Gambia, The	Seychelles
	Ghana	Sri Lanka
	Grenada	St. Kitts and Nevis
	Guatemala	St. Lucia
	Guyana	St. Vincent and the Grenadines
	Honduras	Suriname
	Indonesia	Tanzania
	Iran, Islamic Rep.	Togo
	Isle of Man	Trinidad and Tobago
	Jordan	Uganda
	Kenya	Zambia
Set 3b This list includes countries that are not in the previous list and appear at least once in the WPS index also showed up on the GII for at least 1 year (similar to Set 2)	Benin	India
	Botswana	Morocco
	Brazil	Paraguay
	El Salvador	Peru
	Gabon	Saudi Arabia

##### 4.1.2.4 Inclusion criteria at full text screening (FTS) stage

Given constraints in time and scope, for the full‐text screening level, we have developed a more nuanced operationalisation of fragility which takes into account the fact that fragility is not time constant, and decisions need to be made for each country within the context of the year in which the intervention took place. To do so, we applied the following inclusion criteria at full‐text screening:

- Does the country score over 0.6 on GII at the start date of the intervention or under 0.7 on the WPS for studies starting between 2014 and 2020 (if intervention start date not available use publication date)?
- Does the country score over 80.0 on FSI or is listed on WB or OECD list at the start date of the intervention (if intervention start date not available use publication date)? or, if after 2016, does the country belongs to the fourth or fifth quintile of the WPS index (under 0.700)?

We are aware of some of the limitations of the international indices measuring fragility levels. An important limitation for us has been the absence of an index allowing to measure fragility at the community and local level. To address this limitation we have made the decision that if a study was not meeting the FSI, WB or OECD criterion we would look at whether the local level of implementation of the study was meeting our definition of fragility, human security, building peaceful and inclusive societies and overall relevant characteristics of our definition of developing *peaceful and inclusive societies*. A study meeting the characteristics of our definition at the local or community level could then be included on this criterion.

##### 4.1.2.5 Inclusion of refugee populations

Few populations are as inherently vulnerable, fragile, and exposed to conflict as are refugees, including those that have been resettled as well as those that are living in camps. For that reason, this review has taken the decision to include any study whose explicit population is refugees from a country which would otherwise have been included, and are living in a LMIC that would not be otherwise included. An example of that would be the evaluation of an intervention targeting Syrian refugees in Jordan (an LMIC country that would otherwise be excluded on the FCAS measure). Refugees living in a high income country will not be included in the population in our review.

#### 4.1.3 Types of interventions

The main challenge of the definition of the intervention criteria for our SR is the wide range of interventions that fall under the four pillars of UNSCR 1325. In a similar way as for the population, including all the gender specific or transformative interventions that fall under the four pillars of UNSCR 1325 would be too broad. A set of exclusion grounds has then been agreed:

- We recognise the importance of the Health sector for women empowerment but considering the broad range of literature on this topics and existing research, Health will not be included in the SR
- We will not include studies focusing on the *treatment* of Sexual and Gender‐Based Violence (SGBV) and will only include studies focusing on preventing SGBV
- We recognise the definition of the four pillars of UNSCR 1325 but will adopt a specific list of interventions under some pillars:
  a. Participation: we will broaden the scope of the pillar to include interventions targeting economic, social and political participation
  b. Protection: we will narrow inclusion to efforts to combat SGBV
  c. Recovery and Relief: we will narrow inclusion to reintegration intervention and Disarmament, Demobilisation, Reintegration (DDR)

We use an intervention typology based on the four pillars to specify interventions groupings we will include in our review. We will not repeat the descriptions of all interventions here, but Table 7 summarises included interventions under each pillar.

**Table 7:** List of included interventions for the systematic review

Pillar	Intervention type
Participation	Civil society, associations and networks
	Land rights
	Formal and non‐formal education
	Technical and vocational education and training
	Financial inclusion
	MSMEs and entrepreneurship support
	Cash‐based approaches to support access to and participation in education and/or the economy
	Voice and participation in local and subnational governance and development bodies
	Community and leisure activities
	Civic education and youth leadership
Prevention	Conflict early warning systems
	Dialogue groups
	Community consultations
	Capacity building for conflict resolution
	Peace education
Protection	Legal rights education
	Behaviour change communication around support for women's rights and preventing SGBV
	Preventative protection measures
	Capacity building and technical support to subnational government officials to strengthen service provision for women and gender equality
	Gender‐sensitive policing
	Informal judicial system
Recovery and relief	(Re)integration of forcibly displaced populations
	Disaster risk reduction
	Disarmament, demobilisation and reintegration

The following interventions do not meet the inclusion criteria and will be excluded from the review:

- *SGBV punishment*: we will focus our work on proactive interventions for women's empowerment and gender equality and exclude reactive interventions focusing on the treatment of violence or inequality.
- *Formal peace processes*: this component would mainly focus on the higher levels of the ecological framework and we would only include the community and lower levels for inclusion.
- *Formal judicial system*: this group is excluded on the basis of the ecological framework.
- *Sexual and gender‐based violence (SGBV)—medical and psychosocial treatment and services for victims*: this group is excluded on the basis that Health‐focused intervention will not be included in this study and we will not focus on treatments of SGBV.
- *Family planning/reproductive rights*: this may be included if it is a component of a larger intervention but excluded it the intervention exclusively focuses on it.
- *Capacity building of national‐level actors on the importance of and how to support women's empowerment and gender equality*: this group is excluded on the basis of the ecological framework.
- *Women's health*: health will not be covered in this review.
- *Education in emergencies*: Education will only be covered in the aspect of peace education but not general education.
- *Monetary Policy*: these interventions are rarely gender‐specific and mainly are concentrated at the national/society level.
- *Cash‐based approaches to meet basic needs*: these may be included if it is a component of a larger intervention but excluded if the intervention exclusively focuses on it.
- *Humanitarian aid and emergency response*: this intervention type is too broad and we will exclusively focus on conflict‐related emergencies.

Both our list of inclusion and exclusion are not exhaustive and we therefore expect these lists to change based on the results of our search and screening process.

#### 4.1.4 Types of comparison

We will include studies that compare the effects of an intervention aiming to empower women in fragile and/or conflict‐affected situations against similar situations where individuals, households and/or communities either received a “placebo” intervention or “business as usual” conditions.

#### 4.1.5 Types of outcome measures

The review takes a rights‐based approach that recognises the importance of gender equality as a priority end in and of itself. Given the expected limited evidence for effects of gender equality programmes on outcomes of sustainable peace, the primary objective of the review will be to understand the effects on women's empowerment and gender equality. Secondary objectives include understanding effects on outcomes of sustainable peace, human security and wellbeing.

Given our use of Kabeer's (and Senn's) three pronged definition of women's empowerment, we have developed a taxonomy of outcomes organised by category (Agency, Resources, and Achievement), subcategory, and specific outcome. The subcategories are organised as follows:

- Agency is defined as the ability to act upon one's goals, but also be able to bring meaning, motivation and purpose to an activity. The outcomes have divided into the following subcategories:
  a. Individual Agency
  b. Community Level Agency
  c. Institutions Supporting Agency
- Resources, under Kabeer's umbrella, is meant to be defined broadly as defined to include not only access, but also future claims, to both material and human and social resources.The outcomes have divided into the following subcategories:
  a. Access to Justice and Legal Services
  b. Economic and Livelihoods Related Resources
  c. Access to Employment
- Achievements refer to the capabilities that underlie both agency and resources, as a means of facilitating their procurement. The outcomes have divided into the following subcategories:
  a. Improved Systems
  b. Norms and Behaviour Change

The complete list of outcomes, their definitions and associated UNSCR 1325 pillars is found in Appendix G.

##### 4.1.5.1 Duration of follow‐up

We will not include or exclude studies based on duration of follow‐up. If studies include multiple follow‐ups we will include the outcomes measures most similar to that presented in the other studies included in any single meta‐analysis, and report any additional follow‐ups narratively.

#### 4.1.6 Types of settings

We include interventions started between 2000 and 2020 conducted in FCAS in L&MICs only. We have noted above that fragility varies over time and within states. In order to identify interventions implemented in fragile situations, we will operationalise the following approach. First, to facilitate initial screening, we developed a list of eligible L&MICs that have been classified as fragile according to one of a few state‐level lists For TAS criteria, we include studies where the population falls within at least one of the following categories:

- L&MICs that are listed on all our indicators: in the World Bank list of FCAS (since 2006), on the OECD list of FCAS (since 2007), on the FSI with a score higher than 80 (since 2009) and that have a score of at least 0.6 on the GII for at least 1 year since 2000
- L&MICs that are listed in one of the fragility lists or had at least 1 year rated higher than 80 on FSI and that have a score of at least 0.6 on the GII for at least 1 year since 2000
- L&MICs that do not appear on the fragility lists but are listed with a score lower than 0.6 on the Women Peace Security Index between 2016 and 2019

For FTS criteria, the call for inclusion will be made on a series of criteria depending on the characteristics of the population on the 1st year of implementation against the following indicators:

- L&MIC status of the country of implementation
- GII score
- List of FCAS according to the FSI, World Bank and OECD
- Nuanced definition of fragile contexts applied in previous work
- Specific local context

An additional point of interest is the emphasis on fragile and conflict‐affected *situations*, rather than nation‐states. As a result, countries that do not score within our inclusion criteria may still be included in the final list of studies due to a variety of reasons. As indices are often capturing national‐level data, as a research team we seek to recognise that subnationally, the context may be varying including in situations such as refugee camps in non‐LMICs or rural, unstable regions in non fragile states.

#### 4.1.7 Other

We will include both completed and ongoing studies, such as protocols of ongoing studies that appear to meet all other inclusion criteria or studies listed in registries of ongoing impact evaluations.

We will include studies published in any language, although search terms will be in English only. We will include studies published in 2000 or after, including interventions implemented in 2000 or after, taking the UNSCR 1325 as the starting point of our study.

### 4.2 Search methods for identification of studies

A comprehensive search of the literature for a SR on a topic in international development should cover key bibliographic databases, those specific to international development, those specific to social sciences, and specific to the subject of the review (Kabeer & Waddington, 2015). The search strategy has been developed in collaboration with an information specialist and with reference to guidance in Hammerstrøm et al. (2010).

In order to capture the relevant literature as comprehensively as possible, we have developed both a general set of search terms and series of substrategies and terms grouping around the typologies of the types of interventions included in our review, gender empowerment and gender equality, and building peaceful and inclusive societies. All searches will be limited by the list of countries filters and by the year from 2000 onwards and will integrate a diverse range of literature: articles, impact evaluations, reports, dissertations, conference documents, and so forth.

#### 4.2.1 Electronic searches

We will search the following academic databases:

- Africa‐Wide (Ebsco): https://www.ebsco.com/products/research-databases/africa-wide-information
- CAB Global health: https://www.cabi.org/publishing-products/global-health/
- Communication & Mass Media: https://www.ebsco.com/academic-libraries/subjects/communication-mass-media
- Econlit: https://www.aeaweb.org/econlit/
- Embase: https://www.embase.com/login
- Gender Studies: https://www.ebsco.com/products/research-databases/gender-studies-database
- International Political Science Abstracts: https://www.ebsco.com/products/research-databases/international-political-science-abstracts
- Medline: https://pubmed.ncbi.nlm.nih.gov/
- PsycInfo: https://www.apa.org/pubs/databases/psycinfo
- REpec: http://www.repec.org/
- Web of Science (SSCI): https://clarivate.com/webofsciencegroup/solutions/webofscience-ssci/
- World Bank e‐library: https://elibrary.worldbank.org/

#### 4.2.2 Grey literature searches

We will search the following specialist organisational databases:

1. CARE International: http://www.careevaluations.org/
2. Catholic Relief Services: https://www.crs.org/our-work-overseas/research-publications
3. Centre for Public Impact: https://www.centreforpublicimpact.org/observatory/
4. Chemonics International: https://www.chemonics.com/technical-areas/democracy-and-governance/
5. EGAP (Evidence in Governance and Politics): http://egap.org/biblio
6. IRC: https://www.rescue.org/reports-and-resources
7. LSE ICG: https://www.theigc.org/search/?select-post_type%5B%5D=publication
8. Mercy Corps: https://www.mercycorps.org/research
9. Oxfam International: https://policy-practice.oxfam.org.uk/publications
10. Search for Common Ground: https://www.sfcg.org/ilt/evaluations/
11. FHI360: https://www.fhi360.org/
12. World Vision: http://www.wvi.org/resources

Bilateral and multilateral agencies and general repositories of impact evaluations in international development to be searched include:

1. 3ie Repository of Impact Evaluations: https://developmentevidence.3ieimpact.org/
2. 3ie RIDIE (Registry for International Development Impact Evaluations):: http://ridie.3ieimpact.org/
3. African Development Bank (AfDB): https://www.afdb.org/en/documents/publications/
4. AgEcon: https://ageconsearch.umn.edu/?ln=en
5. AGRIS: http://agris.fao.org/agris-search/index.do
6. Asian Development Bank (ADB): https://www.adb.org/publications
7. BREAD: http://ibread.org/bread/papers
8. Center for Effective Global Action (CEGA): http://cega.berkeley.edu/evidence/
9. CGIAR: Consultative Group on International Agricultural Research: https://cgspace.cgiar.org/handle/10568/83389
10. Design, Monitoring and Evaluation for Peace: www.dmeforpeace.org/learn/resources/
11. DEval: https://www.deval.org/en/home.html
12. DFID Research for Development (R4D): http://r4d.dfid.gov.uk/
13. GEF (Global Environmental Facility) evaluation database: http://www.gefieo.org/evaluations/all?f[0]=field_ieo_grouping%3A312
14. Global Facility for Disaster Reduction and Recovery: https://www.gfdrr.org/en/publications
15. ICNL Research Centre: http://www.icnl.org/research/library/ol/
16. IFPRI: http://www.ifpri.org/publications
17. Independent Development Evaluation, AfDB: http://idev.afdb.org/en/page/evaluations
18. Innovations for Poverty Action (IPA): http://www.poverty-action.org/search-studies
19. Inter‐American Development Bank Publications: https://publications.iadb.org/facet-view?locale-attribute=en%26field=type_view
20. J‐Poverty Action Lab (J‐PAL): https://www.povertyactionlab.org/evaluations
21. Locus (International Development Coalition): https://locus.ngo/resources
22. OECD: http://www.oecd.org/derec/?hf=5%26b=0%26s=score
23. UNEG Database of Evaluation Reports: http://www.uneval.org/evaluation/reports
24. USAID: https://dec.usaid.gov/dec/search/SearchResults.aspx?q=KERvY3VtZW50cy5CaWJ0eXBlX05hbWU6KCgiU3BlY2lhbCBFdmFsdWF0aW9uIikgT1IgKCJGaW5hbCBFdmFsdWF0aW9uIFJlcG9ydCIpKSk%3Cunderline%3E=%3C/underline%3E

Our methodology for the grey literature search will follow a systematic approach:

1. First, a list of the most common variants of the intervention terms should be extrapolated based on any scoping work and existing studies for inclusion identified. Considering the broad aspect of our review we will use a short set of terms related to gender equality and women's empowerment.
2. The strategy differs for sites with more or <300 resources. First, any relevant filters within the specific site should be applied (e.g., choosing only relevant document types, sectors, etc.).
  a. For repositories with more than 300 resources:
    i. Each search term is run alongside the methodology term “impact evaluation” and “impact assessment” for a total of 10 searches per site (women, men, girls, boys and gender).
    ii. Screen results until no potentially relevant results are identified on two consecutive pages of 50 search results (i.e. 100 excludes in a row).
  b. For repositories with fewer than 300 resources:
    i. Only the methodology term “impact evaluation” is searched.
    ii. Screen all results.
3. For all sites, where possible, display settings are set to show 50 results per page, and the first page of results gets printed to PDF. Each set of PDFs gets saved in a folder with the site name and date of search, and the search data get recorded in a master Excel file as follows:
  a. For each website searched, the following information should be recorded:
    i. The date of the search;
    ii. The specific URL of the website searched (e.g., to differentiate between a search of publications vs a search of programmes, which on J‐PAL's website will return different results);
    iii. The specific search terms used in each search (e.g., 1: impact evaluation; 2: “impact evaluation”; 3… etc.);
    iv. The number of hits for each search result (which can be recorded in a single cell with a formula, =[no. results from first search]+[no. results from second search]+…+[no. results from nth search]);
    v. the number of potentially relevant documents identified and uploaded to EPPI, recorded by search as in (iv)
4. For each set of results, the titles and any abstract information shown on the main page are screened for potential inclusion, and if clearly irrelevant based on intervention, methodology or population, are ignored. If the title indicates possible relevance, click through to the full‐text and screen the abstract or executive summary.
5. Any result that appears of potential relevance gets downloaded and saved in the GLS shared folder for that site.
6. The documents collected from the GLS are then added to the project's EPPI environment.
  a. Create a new record in EPPI for each identified study.
  b. Key bibliographic information should be added: Title, all authors, year of publication, URL to the full text, and publisher. Abstract is not required.
  c. Once the bibliographic info is added, click “save,” which will then allow you to upload the full text.
  d. The study should be marked as “include” at TAS and coding marked as completed (with the record open in EPPI, right‐click the heading for TAS, select “Properties” then check the box next to “Coding completed”)
  e. The study should be marked as grey literature search under “origin”

This process will be done by a core member of the team if possible, up to the point of creating records in EPPI, which can be assigned to a consultant. If it is not possible, then it will be assigned to a consultant or two, who are deemed to have a strong understanding of the inclusion criteria. When information is not directly available on the listed website we will make use of Google Search (especially Google Scholar) for the retrieval of missing and/or additional references.

Given the breadth of our SR we are going to trial web scraping tools at the beginning of the search. We will trial the use of a data *miner* to extract grey literature reference that will then be screened through EPPI:

- The data scraping will only be tested on platforms presenting both the Title and Abstract of the listed reports and resources
- The consultant will follow the same approach as for the standard Grey Literature Method and save a PDF copy of the results of the search term
- Using data miner, the consultant will then extract the data of the results pages to an excel template that would allow the upload of all the results on EPPI for TAS

We will pilot this web scraping approach by testing it on similar websites to the standard method and assess the interest in scaling it up.

#### 4.2.3 Searching other resources

##### 4.2.3.1 General approach

- We will screen the bibliographies of included studies and existing reviews for additional eligible studies and will conduct forwards citation‐tracking of included studies in Google Scholar:
- Backwards citation search: Comb through the list of references in the study's full text, and see if you can identify any potentially relevant studies from the titles, looking up any that look even possibly relevant to check their abstract. You will need to be quite inclusive in terms of what could be relevant, since in many cases its very hard to tell from a title what the reference relates to. If you have Chrome, I would suggest installing the Google Scholar add‐on button, which lets you highlight a reference on a webpage, and then by clicking the button, it will automatically search for it in Google Scholar and usually show you something of the abstract. This can make this process go a lot more quickly.
- Forwards citation search: Use Google Scholar to find the included paper, then under the entry, click the hyperlink where it says "Cited by XXX" and screen the subsequent list of papers citing the included study. Where we have multiple included papers for a single study, you'll need to do this for each of them. Again, try to be relatively inclusive in terms of what looks relevant, though it's a bit easier here as you should see at least some of each abstract.

This citation search will apply to the studies shortlisted through the TAS and Grey Literature search and, considering the potential size of our sample not to the retrieved literature and citations selected for analysis through the FTS. Considering the potential size of our sample we will only do one level of citation search and will not do rounds of citation searches until no resources can be found.

We will also publish a blog post presenting our SR and calling for provision of includable studies from the international development and humanitarian community. We will also identify and contact key researchers and organisations working in the relevant fields of our study for suggestion and will engage our advisory group for suggestion of relevant studies. We will also hand‐search journals of particular relevance to the review in an effort to identify papers that have not yet been indexed, covering issues published in the last 12 months. This hand‐search will also be based on journals citing the included studies.

Titles and abstracts will be screened against the inclusion criteria and relevant records will be downloaded into the review management software EPPI reviewer. The initial screening of records will be conducted by several reviewers screening the records from different databases. At this stage we will be over‐inclusive to ensure relevant studies are not omitted because sufficient information is not reported in title or abstract. Two reviewers will then independently review abstracts that have been judged to be potentially relevant at the first stage in more detail to determine which papers should be retrieved and reviewed at full text. Two reviewers will then independently assess full text studies for inclusion, with any disagreements resolved by a third reviewer.

##### 4.2.3.2 Targeted search for addressing review questions 3 and 4

Once we have identified our set of included impact evaluations, we will undertake targeted searching for qualitative studies, process evaluations, and project documents for those interventions evaluated in the included studies. We will conduct citation tracking of included studies to identify relevant sister papers and conduct internet and database searches using the names of programmes from included studies. To identify project documents and process evaluations we will conduct targeted searches of databases of project documents and websites of implementing agencies. We will also try to contact authors and implementing agencies to get access to specific studies.

### 4.3 Data collection and analysis

#### 4.3.1 Description of methods used in primary research

Using the inclusion criteria set out in the previous sections, we anticipate that primary studies included in this review will use experimental or quasi‐experimental study designs and/or analysis methods to examine the extent to which changes in outcomes can be attributed to the intervention under study. To this end, we will include randomised studies as well as non‐randomised studies that are able to suitably account for selection and confounding bias (Waddington et al., 2017).

#### 4.3.2 Criteria for determination of independent findings

Complex data structures are a common occurrence in meta‐analyses of impact evaluations. There are several scenarios through which these complex structures with dependent effect sizes might occur. For instance, there could be several publications that stem from one study, or several studies based on the same data set. Some studies might have multiple treatment arms that are all compared to a single control group. Other studies may report outcome measurements from several time points, or use multiple outcome measures to assess related outcome constructs. All such cases yield a set of statistically dependent effect size estimates (Borenstein et al., 2009).

The research team will assess the extent to which relationships exist across the studies included in the review. We will avoid double counting of identical evidence by linking papers prior to data analysis. Where we have several publications reporting on the exact same effect, we will use effect sizes from the most recent publication. We will utilise information provided in studies to support these assessments, such as samples sizes, programme characteristics and key implementing and/or funding partners.

We will extract effects reported across different outcomes or subgroups within a study, and where information is collected on the same programme for different outcomes at the same or different periods of time, we will extract information on the full range of outcomes over time. Where studies report effects from multiple model specifications, we will use the author's preferred model specification. If this is not stated or is unclear, we will use the specification with the most controls. Where studies report multiple outcome subgroups for the same outcome construct, we may calculate a “synthetic effect size” (Borenstein et al., 2009, ch. 24). Where studies report multiple outcomes or evidence according to subgroups of participants, we will record and report data on relevant subgroups separately. Further information on criteria for determining independent effect sizes is presented below.

We will deal with dependent effect sizes in one of two ways, either through the use of robust variance estimation (RVE; Fisher & Tipton, 2015; Hedges et al., 2010), or through data processing and selection techniques. RVE using a small sample adjustment will be the preferred analytic method when feasible. The RVE approach allows us to use all available data in our effect size estimates, even data that is statistically dependent. However, these analyses must have >4 degrees of freedom in order to make valid inferences. In cases where analyses do not meet this criteria, data processing and selection techniques will be used to deal with dependent effect sizes.

If RVE analyses are not feasible for a meta‐analysis of any given intervention or outcome group, we will utilise several criteria to select one effect estimate per study. Where we have several publications reporting on the same study, we will use effect sizes from the most recent publication. For studies with outcome measures at different time points, we will follow Rue et al. (2013) and synthesise outcomes measured immediately after the intervention (defined as 1–6 months) and at follow‐up (longer than 6 months) separately. If multiple time points exist within these time periods, we will use the most recent measure. We anticipate many of the interventions we include in our review will be ongoing programmes and the follow‐up will, therefore, reflect duration in a programme rather than time since intervention. When such studies report outcome measures at different time points, we will identify the most common follow‐up period and include the follow up measures that match this most closely in the meta‐analysis. When studies include multiple outcome measures to assess related outcome constructs, we will follow Macdonald et al. (2012) and select the outcome that appears to most accurately reflect the construct of interest without reference to the results. If studies include multiple treatment arms with only one control group and the treatments represent separate treatment constructs, we will calculate the effect size for treatment A versus control and treatment B versus control and include in separate meta‐analyses according to the treatment construct. If treatments A and B represent variations of the same treatment construct, we will calculate the weighted mean and *SD* for treatment A and B before calculating the effect size for the merged group versus control group, following the procedures outlined in Borenstein et al. (2009, ch. 25). Where different studies report on the same programme but use different samples (e.g., from different regions) we will include both estimates, treating them as independent samples, provided effect sizes are measured relative to separate control or comparison groups.

#### 4.3.3 Selection of studies

We will begin by importing all search results into EPPI‐Reviewer 4 (Thomas & Brunton, 2010) and removing duplicates through the EPPI‐Reviewer 4 deduplication process. We will double screen at title and abstract for the first 10% of search results, including any studies we know will be included, in order to train the machine learning (ML) algorithm. In this review, we will take advantage of an innovative text‐mining ML capability of EPPI‐Reviewer 4 to reduce the initial screening workload: the inclusion/exclusion classifier (O'Mara‐Eves et al., 2015; Thomas et al., 2011).

We will utilise the inclusion/exclusion classifier in order to organise studies into groups based on their probability of inclusion in the review. We will conduct piloting and verification of the machine learning functioning and based on our experience in previous reviews, we expect to be able to exclude all studies with <20% probability of inclusion automatically from the review. We will screen a random 10% sample of the automatically excluded studies to double check the accuracy of the function, and if all are excludable, we will auto‐exclude the rest. We will then double‐screen at title and abstract all records with likelihood of inclusion at 20% or greater.

Where a study's title and abstract do not include sufficient information to determine relevance, we will include the study for review at full text. We will double screen all studies flagged for full‐text review using two independent reviewers. We will resolve disagreements on inclusion or exclusion by discussion with a core review team member and the input of an additional core reviewer if necessary. We will assess the results of the study‐specific key‐word searches for relevance, that is, whether they cover one of the programmes included to answer our research questions and whether they provide information on the design, implementation processes, context or mechanisms at play.

We also expect to identify multiple papers related to the same study. In this case we will use the Linked studies functionality of EPPI reviewer to identify the main study and other linked study. The main study will be the study used for data extraction and the linked studies will complement the potential missing information of the main studies. To identify the main study, the priority will be given to journal articles, in the case of multiple journal articles or only reports/working papers the most comprehensive will be selected. In the case of equivalent comprehensivity of the paper, the most recent paper will be selected.

#### 4.3.4 Data extraction and management

We will extract the following descriptive, methodological, qualitative and quantitative data from each included study using standardised data extraction forms (provisional forms are provided in Appendix B):

- Descriptive data including authors, publication date and status, as well as other information to characterise the study including country, type of intervention and outcome, population, and context.
- Methodological information on study design, analysis method, and type of comparison (if relevant).
- Quantitative data for outcome measures, including outcome descriptive information, sample size in each intervention group, outcomes means and *SD*s, and test statistics (e.g., *t* test, *F* test, *p* values, 95% confidence intervals).
- Information on intervention design, including how the intervention incorporates participation, inclusion, transparency and accountability characteristics, participant adherence, contextual factors, and programme mechanisms.

We will extract all quantitative, qualitative, descriptive, and methodological data using Excel. Descriptive and qualitative data will be single coded by one reviewer and checked by a second reviewer. Two independent reviewers will double code quantitative data for outcomes analysis and risk of bias assessments, and any disagreement will be resolved through discussion with a third reviewer (who must be a core team member).

Once all effect sizes are calculated and converted to a standardised mean difference (as described in detail below), we will examine the data for outliers. We will define outliers as any effect sizes ±3.29 *SD*s from the mean, following the guidance of Tabachnick and Fidell (2007). Outliers will be windsorised as described by the authors, and as is suggested for outliers in meta‐analysis (Lipsey & Wilson, 2001). We will examine sensitivity to outliers as discussed in the section on sensitivity analysis below.

For the qualitative analysis, we will extract detailed data on population characteristics, intervention design and implementation, and contextual variables (e.g., region, political climate) to address questions 3 and 4.

#### 4.3.5 Assessment of risk of bias in included studies

We will assess the risk of bias in the included studies by drawing on the signalling questions in the 3ie risk of bias tool, which covers both internal validity and statistical conclusion validity of experimental and quasi‐experimental impact evaluation designs (Waddington et al., 2012a). It includes the bias domains and extensions to Cochrane's ROBINS‐I tool and RoB2.0 (Higgins et al., 2016; Sterne et al., 2016). The risk of bias assessment helps us to determine the extent to which the findings in each study are reliable. Two reviewers will undertake the risk of bias assessment independently. If there are disagreements, we will resolve them by discussion and the involvement of a third reviewer (who must be a member of the core team), as necessary. The provisional risk of bias tool can be found in Appendix C. We will do the risk of bias at the paper level.

We will assess risk of bias based on the following criteria, coding each paper as “Yes”, “Probably Yes”, “Probably No”, “No” and “No Information” according to how they address each domain:

- Factors relating to baseline confounding and biases arising from differential selection into and out of the study (e.g., assignment mechanism).
- Factors relating to bias due to missing outcome data (e.g., assessment of attrition).
- Factors relating to biases due to deviations from intended interventions (e.g., performance bias and survey effects) and motivation bias (Hawthorne effects).
- Factors relating to biases in outcomes measurement (e.g., social desirability or courtesy bias, recall bias).
- Factors relating to biases in reporting of analysis.

We will report the results of the assessment for each of the assessed criteria for each study. In addition, we will use the results of the risk of bias assessments to produce an overall rating for each study as either “High risk of bias”, “Some concerns” or “Low risk of bias”, drawing on the decision rules in RoB2.0 (Higgins et al., 2016), rating studies as follows:

- “High risk of bias”: if any of the bias domains were assessed as “No” or “Probably No”.
- “Some concerns”: if one or several domains were assessed as “No Information” and none were “No” or “Probably No”.
- “Low risk of bias”: if all of the bias domains were assessed as “Yes” or “Probably Yes”.

In addition, we will attempt to explore whether there are systematic differences in outcome effects between primary studies with different risk of bias. If meta‐analysis is feasible, we will conduct sensitivity analysis to assess the robustness of the results to the risk of bias in included studies.

#### 4.3.6 Measures of treatment effect

An effect size expresses the magnitude (or strength) and direction of the relationship of interest (Borenstein et al., 2009; Valentine et al., 2015). We will extract data from each individual study to calculate standardised effect sizes for cross‐study comparison wherever possible. For continuous outcomes comparing group means in a treatment and control group, we will calculate the standardised mean difference (SMDs), or Cohen's *d*, its variance and SE using formulae provided in Borenstein et al. (2009). A SMD is a difference in means between the treatment and control groups divided by the pooled *SD* of the outcome measure. Cohen's *d* can be biased in cases where sample sizes are small. Therefore, in all cases we will simply adjust *d* using Hedges’ method, adjusting Cohen's *d* to Hedges’ *g* using the following formula (Ellis, 2010):

$$g≅d\left(1-\frac{3}{4(n_{1}+n_{2})-9}\right)$$

We will choose the appropriate formulae for effect size calculations in reference to, and dependent upon, the data provided in included studies. For example, for studies reporting means (*X*) and pooled *SD* for treatment (T) and control or comparison (C) at follow up only:

$$d=\frac{x_{\mathrm{Tp}+1}-x_{\mathrm{Cp}+1}}{\mathrm{SD}}$$

If the study does not report the pooled *SD*, it is possible to calculate it using the following formula:

$${\mathrm{SD}}_{p+1}=\sqrt{\frac{(n_{\mathrm{Tp}+1}-1){\mathrm{SD}}_{\mathrm{Tp}+1}^{2}+(n_{\mathrm{Cp}+1}-1){\mathrm{SD}}_{\mathrm{Cp}+1}^{2}}{n_{\mathrm{Tp}+1}+n_{\mathrm{Cp}+1}-2}}$$

Where the intervention is expected to change the *SD* of the outcome variable, we will use the *SD* of the control group only.

For studies reporting means ($\underline{X})$ and *SD*s for treatment and control or comparison groups at baseline (p) and follow up (p + 1):

$$d=\frac{{∆\underline{X}}_{p+1}-{∆\underline{X}}_{p}}{{\mathrm{SD}}_{p+1}}$$

For studies reporting mean differences ($∆\underline{X})$ between treatment and control and *SD* at follow up (p + 1):

$$d=\frac{{∆\underline{X}}_{p+1}}{{\mathrm{SD}}_{p+1}}=\frac{{\underline{X}}_{\mathrm{Tp}+1}-{\underline{X}}_{\mathrm{Cp}+1}}{{\mathrm{SD}}_{p+1}}$$

For studies reporting mean differences between treatment and control, *SE* and sample size (*n*):

$$d=\frac{{∆\underline{X}}_{p+1}}{\mathrm{SE}\sqrt{n}}$$

As primary studies have become increasingly complex, it has become commonplace for authors to extract partial effect sizes (e.g., a regression coefficient adjusted for covariates) in the context of meta‐analysis. For studies reporting regression results, we will follow the approach suggested by (Keef & Roberts, 2004) using the regression coefficient and the pooled *SD* of the outcome. Where the pooled *SD* of the outcome is unavailable, we will use regression coefficients and *SE*s or *t* statistics to do the following, where sample size information is available in each group:

$$d=t\sqrt{\frac{1}{n_{T}}+\frac{1}{n_{C}}}$$

where *n* denotes the sample size of treatment group and control. We will use the following where only the total sample size information (*N*) is available, as suggested in (Polanin et al., 2016):

$$d=\frac{2t}{\sqrt{N}}{\mathrm{Var}}_{d}=\frac{4}{N}+\frac{d^{2}}{4N}$$

We will calculate the *t*‐statistic (*t*) by dividing the coefficient by the *SE*. If the authors only report confidence intervals and no *SE*, we will calculate the *SE* from the confidence intervals. If the study does not report the *SE*, but report *t*, we will extract and use this as reported by the authors. In cases in which significance levels are reported rather than *t* or SE (b), then *t* will be imputed as follows:

Prob > 0.1: *t* = 0.5

0.1 ≥ Prob > 0.05: *t* = 1.8

0.05 ≥ Prob > 0.01: *t* = 2.4

0.01 ≥ Prob: *t* = 2.8, where outcomes are reported in proportions of individuals, we will calculate the Cox‐transformed log odds ratio effect size (Sánchez‐Meca et al., 2003):

$$d=\frac{\ln(\mathrm{OR})}{1.65}$$

where OR is the odds ratio calculated from the two‐by‐two frequency table.

Where outcomes are reported based on proportions of events or days, we will use the standardised proportion difference effect size:

$$d=\frac{p_{T}-p_{C}}{\mathrm{SD}(p)}$$

where *p*_*t*_ is the proportion in the treatment group and *p*_*c*_ the proportion in the comparison group, and the denominator is given by:

$$\mathrm{SD}(p)=\sqrt{p(1-p)}$$

where p is the weighted average of *p*_*c*_ and *p*_*t*_:

$$p=\frac{n_{T}p_{T}+n_{C}p_{C}}{n_{T}+n_{C}}$$

An independent reviewer will evaluate a random selection of 10% of effect sizes to ensure that the correct formulae were employed in effect size calculations. In all cases after synthesis, we will convert pooled effect sizes to commonly used metrics such as percentage changes and mean differences in outcome metrics typically used (e.g., weight in kg) whenever feasible.

#### 4.3.7 Unit of analysis issues

Unit of analysis errors can arise when the unit of allocation of a treatment is different to the unit of analysis of effect size estimate, and this is not accounted for in the analysis (e.g., by clustering *SE*s at the level of allocation). We will assess studies for unit of analysis errors (The Campbell Collaboration, 2019), and where they exist, we will correct for them by adjusting the *SE*s according to the following formula (Higgins & Thomas, 2020; Waddington et al., 2012; Hedges, 2009):

$$\mathrm{SE}{\left(d\right)}^{\prime }=\mathrm{SE}(d)⁎\sqrt{1+(m-1)c}$$

where m is the average number of observations per cluster and c is the intra‐cluster correlation coefficient. Where included studies use robust Huber‐White *SE*s to correct for clustering, we will calculate the *SE* of *d* by dividing *d* by the t‐statistic on the coefficient of interest.

#### 4.3.8 Dealing with missing data

In cases of relevant missing or incomplete data in studies identified for inclusion, we will make every effort to contact study authors to obtain the required information. If we are unable to obtain the necessary data, we will report the characteristics of the study but state that it could not be included in the meta‐analysis or reporting of effect sizes due to missing data.

#### 4.3.9 Assessment of heterogeneity

We will assess heterogeneity by calculating the *Q* statistic, *I* ^2^, and *τ* ^2^ to provide an estimate of the amount of variability in the distribution of the true effect sizes (Borenstein et al., 2009). We will complement this with an assessment of heterogeneity of effect sizes graphically using forest plots. Additionally, we will explore heterogeneity using moderator analysis in meta‐regression specifications.

#### 4.3.10 Assessment of reporting biases

In order to reduce the possibility of publication bias, we will search for and include unpublished studies in the review. We will also test for the presence of publication bias through the use of contour‐enhanced funnel graphs (Peters et al., 2008) and statistical tests (Egger et al., 1997). Capitalising on recent shifts towards pre‐registration of studies and their associated pre‐analysis plans, we will also examine whether studies that were pre‐registered (e.g., on platforms such as ClinicalTrials.gov, the Open Science Foundation, the American Economic Association's trial registry, or the Registry for International Development Impact Evaluations (RIDIE)) report on all of the outcomes that were proposed in their pre‐analysis plans. This additional analysis of outcome reporting bias may draw on methodologies used in previous work, such as the COMPare Trials Project (Goldacre et al., 2016).

#### 4.3.11 Data synthesis

We will conduct meta‐analyses of studies that we assess to be sufficiently similar. The inclusion criteria for the review are broad and we anticipate including studies that report on a diverse set of interventions, sectors and outcomes. It is therefore difficult to predict how meta‐analysis will be used in the review prospectively. However, minimum criteria will be to only combine studies using meta‐analysis when we identify two or more effect sizes using a similar outcome construct and where the comparison group state is judged to be similar across the two, similar to the approach taken by (Wilson et al., 2011). We provisionally suggest that we combine studies in the same analysis when they evaluate the same intervention type, or the same outcome type. Moderator analyses can take into account multiple interventions as moderator variables, allowing us to also examine the impact of different intervention types by outcome. Where there are too few studies, or included studies are considered too heterogeneous in terms of interventions or outcomes, we will present a discussion of individual effect sizes along the causal chain. As heterogeneity exists in theory due to the variety of interventions and contexts included, we will use inverse‐variance weighted, random effects meta‐analytic models (Higgins & Thomas, 2020).

We will use the metafor package (Viechtbauer, 2010) and/or the robumeta package (Fisher & Tipton, 2015) in R software to conduct the meta‐analyses (R Core Team, 2020).

We will conduct separate analyses for the major outcome categories: primary outcome (women's empowerment, gender equality), secondary outcome (well being, security, peace) and other important outcomes such as changes in knowledge (of rights), attitudes and behaviours, tolerance of violence, self‐confidence, self‐efficacy. Based on an analysis of the interventions that we find, we will attempt to further elaborate on the pathway of change that was outlined above to the extent possible. We will also use subgroup analysis to explore heterogeneity by different treatment subgroups (described in more detail in the section on subgroup analysis and investigation of heterogeneity).

We will also collect qualitative information from studies about the interventions. This information may subsequently be coded quantitatively to be used in moderator analysis. It may also be used to classify intervention mechanisms in synthesis or in the further development of intervention causal chains. These characteristics may include: intervention objectives (to change processes, behaviours or both); whether interventions are strategic (complex, adaptable strategy to realise change) or tactical (tool‐based); the source of intervention (local, NGO, government or researcher‐led); the scale of the intervention (pilot experiment versus adoption of formal policy/law); extent to which members of both targeted groups are engaged (equally or primarily one group); and initial power differences between the groups targeted.

#### 4.3.12 Subgroup analysis and investigation of heterogeneity

Whenever feasible, we will conduct moderator analyses to investigate sources of heterogeneity. Following the PROGRESS‐PLUS approach (Oliver et al., 2017), we will assess moderators falling into three broad categories of extrinsic, methodological and substantive characteristics to address inequity aspects within the gender equality context. Examples of these categories include:

- Extrinsic characteristics: funder of the study (e.g., NGO vs private sector vs government investments), publication type, publication date.
- Methodological characteristics: study design, risk of bias, study quality characteristics, evaluation period, length of follow‐up.
- Substantive characteristics: participant characteristics (gender, age, socio‐economic status, education, land ownership), context (geographical setting, market access), intervention type, intervention features, type of implementing agency.

We will use random effects meta‐regression to investigate the association between moderator variables and heterogeneity of treatment effects (Borenstein et al., 2009) and subgroup analyses to investigate heterogeneity by treatment subgroups (e.g., men and women, poor and non‐poor, and so on). If the latter strategies are not possible (that is, if we do not have sufficient number of studies or data), we will discuss and explore the factors which may be driving heterogeneity of results narratively by conducting cross‐case comparisons (Miles & Huberman, 1994).

The qualitative analysis will also include the following subgroups: women's age, education, marital status, health (currently pregnant or not), ethnically marginalised group, race and caste. For groups, heterogeneity by mixed‐sex groups and women‐only groups. If possible, sex of service/intervention frontline provider.

#### 4.3.13 Sensitivity analysis

We will conduct sensitivity analysis to assess whether the results of the meta‐analysis are sensitive to the removal of any single study. We will do this by removing studies from the meta‐analysis one‐by one and assessing changes in results. We will also assess sensitivity of results to inclusion of high risk of bias studies by removing these studies from the meta‐analysis and comparing results to the main meta‐analysis results. Finally, we will assess sensitivity to outliers by comparing results with and without outliers included, as well as results when outliers are windsorised.

#### 4.3.14 Treatment of qualitative research

As a mixed‐methods SR, we include a distinct review component to synthesise qualitative evidence on the review questions 3 and 4. While the identification of qualitative evidence is limited to studies linked to the included impact evaluations, the process of data extraction, critical appraisal, and evidence synthesis is independent and follows methods and guidelines tailored to the conduct of qualitative evidence synthesis (Noyes et al., 2020). This approach to mixed‐methods synthesis of development interventions builds on Snilstveit et al. (2015).

##### 4.3.14.1 Assessment of quality in descriptive quantitative studies, qualitative studies and process evaluations

We will assess the quality of included qualitative studies, process evaluations, and descriptive quantitative studies using a mixed‐methods appraisal tool developed by Langer et al. (2017) and applied in Snilstveit et al. (2019). This tool builds on the Critical Appraisal Skills Programme checklist (CASP, 2006) and Pluye et al.' (2011) mixed‐methods appraisal tool and is provided in Appendix B. Our appraisal tool will make judgements on the adequacy of reporting, data collection, presentation, analysis and conclusions drawn. The appraisal will assess the quality of the included qualitative studies and descriptive quantitative studies using six appraisal domains:

1. The defensibility of the applied research design to answer the research question under investigation.
2. The defensibility of the selected research sample and the process of selecting research participants.
3. The rigour of the technical research conduct, including the transparency of reporting.
4. The rigour of the applied analysis and credibility of study's claims given the nature of the presented data.
5. The consideration of the study's context (for qualitative studies only).
6. The reflexivity of the reported research (for qualitative studies only).

We will filter out studies of particularly low quality at this stage, using a fatal flaw approach following Dixon‐Woods et al. (2005). Studies that do not meet either criterion of appraisal domains 1–4 above will be excluded from the synthesis. That is, they will be included in the review and we will report on the studies’ descriptive data, for example applied intervention. However, no research findings will be extracted from these studies to feed into the review's synthesis. Each appraisal domain will be assessed from a scale of critical trustworthiness, to low, medium and high trustworthiness. An overall critical appraisal judgement per study will be allocated using a numerical threshold of the appraised quality domains (Appendix B).

We will not undertake a critical appraisal of included project documents. They typically provide information about planned, ongoing or completed programmes, providing information about the design or resources available for a project for instance. As such these documents do not typically include much analysis of primary evidence, but they provide factual information about interventions. The purpose of including them in our review is to ensure we have sufficient information about the context and interventions included in our review. We will therefore focus the appraisal on assessing the relevance of the documents against the interventions assessed in our review. Before extracting any data, we will ensure that the name of the intervention, the implementing agency, context and timeline of the intervention described in the project document corresponds to the intervention assessed in the impact evaluation included in our review. Finally, collecting data from a range of sources, especially if used for triangulation, can enhance confidence in the trustworthiness of the information included. If several sources are available, we will extract data from all sources for purposes of triangulation.

##### 4.3.14.2 Methods for qualitative evidence synthesis (applicable to review questions 3 and 4)

To address questions 3 and 4, we will complement any statistical meta‐regressions with a qualitative evidence synthesis (Noyes et al., 2020). After having completed the detailed coding of all of the included studies as described above, we will assess the coding of data on factors related context, intervention design and implementation, and population characteristics for the most relevant qualitative synthesis approach. The identified approach to qualitative evidence synthesis will align and be informed by the review's overall theory of change as outlined in Figure 2. We expect thematic synthesis (Thomas & Harden, 2008) and qualitative comparative analysis (Rihoux, 2006; Thomas et al., 2014) to present two relevant approaches to the qualitative synthesis in this review.^2^ Each is described in more detail below and the final decision for either approach depends on the nature and size of the available qualitative evidence‐base linked to the identified impact evaluations.

##### 4.3.14.3 Thematic synthesis

This qualitative evidence synthesis approach will be applied in case we have sufficient in‐depth qualitative studies and empirical primary data reported across the identified evidence‐base and linked to groups of interventions and outcomes along the review's theory of change. Its objective will be to identify analytical themes on intervention mechanisms and contexts that mitigate or reinforce intervention effects to complement the review's statistical moderator analysis and/or meta‐regression. Following Thomas and Harden (2008) in the thematic synthesis, we will use inductive coding techniques to first identify common descriptive themes based on the reported findings of the primary studies. We will use EPPI‐Reviewer's coding software to illustrate the link between the inductive codes in the primary studies and the identified descriptive themes. In a second step, following the identification of descriptive themes, these will then be configured into higher level analytical themes, which present the results of the thematic synthesis. Analytical themes will be configured around mechanisms and contexts in relation to research question 3 and 4 of this review. Again, this configuration from descriptive to analytical themes will be conducted in EPPI‐Reviewer and we will produce an overview table of both types of themes and their linkages for transparency in this final synthesis step. The process of configuring descriptive and analytical themes from the inductive coding will apply the same consistency checks as the general data extraction process outlined above.

##### 4.3.14.4 Qualitative comparative analysis

This qualitative evidence synthesis approach will be applied in case we have a paucity of detailed qualitative studies and a larger body of evidence drawn from process evaluations and project reports. QCA investigates the configuration of different conditions and their association with intervention effectiveness (Thomas et al., 2014). However, unlike thematic synthesis (and statistical subgroup analysis), QCA allows us to present and investigate overlapping pathways to causality. That is, QCA does not assess the association of selected conditions (e.g., participatory design) with intervention effectiveness but rather the different configurations of such conditions (e.g., participatory design and social norms) and their association with intervention effectiveness. To do so, QCA applies Boolean logic to establish necessary or sufficient conditions for intervention effectiveness. Conditions in our review will refer to factors associated with intervention context, design and implementation, and population characteristics.

Our approach to QCA will follow the six step method for QCA proposed by Thomas et al. (2014) based on Rihoux (2006). The authors propose the following six steps, but suggest that step five—“consideration of the “logical remainders” cases”—might not be necessary when using QCA in the context of SRs:

1. Building the data table
2. Constructing a “truth table”
3. Resolving contradictory configurations
4. Boolean minimisation
5. Consideration of the “logical remainders” cases
6. Interpretation

We will use the studies included in the SR as our first source to collect relevant conditions to construct the initial data table. In this process of identifying relevant conditions, we will first consult the studies reporting the most and least effective interventions and extract the factors reported in these interventions. Our second source of relevant conditions will be stakeholder engagement through the reviews’ AG.^3^ The process of extracting relevant conditions for QCA will apply the same consistency checks as the general data extraction process outlined above.

We assume to conduct a fuzzy set QCA in which interventions might be partially attributed to conditions. The data table will map the relevant conditions and outcomes for each individual intervention. Following analysis of the data table, we will develop the truth table which displays the conditions, configurations, and the number of studies with membership in each configuration set (Thomas et al., 2014). The truth table itself will then undergo careful analysis to investigate the logical coherence of the identified configuration. Once contradictory configurations are resolved, we will use fs/QCA software's Boolean minimisation algorithm to identify the most consistent and simple configurations of conditions. At this stage in the analysis, we will be able to present a range of configurations, that is, a combination of conditions, which we will interpret for relevance and further consideration in relation to the results of the statistical meta‐analysis. In line with the overall objective of the qualitative evidence synthesis, the results of the QCA are intended as an explanatory supplement to the meta‐analysis findings in response to review questions 3 and 4.

#### 4.3.15 Confidence in cumulative bodies of evidence

To summarise our confidence in the cumulative body of evidence, we will use the Grading of Recommendations Assessment, Development and Evaluation (GRADE) approach for confidence in quantitative bodies of evidence on intervention effects (Guyatt et al., 2008), and we will explore use of the GRADE‐CERQual (Confidence in the Evidence from Reviews of Qualitative research) approach for confidence in qualitative bodies of evidence on barriers, facilitators, and moderators of intervention effectiveness (Lewin et al., 2018).

##### 4.3.15.1 GRADE approach for quantitative bodies of evidence

We will use the GRADE approach to rate our confidence in the body of evidence for each intervention contrasts on all primary and secondary outcomes. We specifically will rate our confidence that the true effect of an intervention (versus a comparator) on a given outcome lies on one side of the line of no effect, or “difference from the null” (Hultcrantz et al., 2017). Accordingly, our ratings will refer to our confidence in the existence (or not) of intervention effects and the direction of effects; they will not refer to our confidence in the magnitude of effects. There will be four possible levels of confidence:

- High confidence: It is highly likely that the intervention does (not) have an effect on the outcome of interest.
- Moderate confidence: It is likely that the intervention does (not) have an effect on the outcome of interest.
- Low confidence: It is possible that the intervention does (not) have an effect on the outcome of interest.
- Very low confidence: It is not clear whether the intervention does (not) have an effect on the outcome of interest.

We will follow the traditional GRADE approach, in which evidence from RCTs starts at high confidence, while evidence from all other study designs starts as low confidence. We will assess the body of evidence according to five factors that can lead to a downgrading confidence in the evidence by one level (serious concern) or two levels (very serious): limitations in individual studies (risks of bias), inconsistency of results, indirectness of evidence, imprecision, and publication bias. We will then assess the body of evidence according to three factors that can lead to an upgrading confidence in the evidence: large magnitude of an effect, a dose‐response gradient, and the effect of plausible residual confounding. We will follow recent guidance on how to consider these factors when adopting a complexity perspective in a SR on intervention effects (Montgomery et al., 2019), such as using sources of intervention complexity to explain substantial heterogeneity, and using information on intervention implementation to inform assessments of a dose‐response gradient.

We will present and explain the results of our GRADE ratings in summary of findings tables (Santesso et al., 2016). In addition, we will describe our GRADE ratings narratively using language approved by the GRADE Working Group (Santesso et al., 2020):

- High confidence: The intervention reduces/increases the outcome.
- Moderate confidence: The intervention probably reduces/increases the outcome.
- Low confidence: The intervention may reduce/increase the outcome.
- Very Low confidence: We are very uncertain about the effect of the intervention on the outcome.

##### 4.3.15.2 Confidence in the body of evidence included in the qualitative review component

We will explore the relevance and feasibility of conducting a confidence in the body of evidence assessment for the qualitative review component. The decision to conduct this assessment will depend on the size and nature of the included qualitative evidence‐base as well as the contribution of the qualitative review findings to the stated research questions. If relevant and feasible, we aim to apply the GRADE‐CERQual (Lewin et al., 2018) to conduct the confidence of the evidence assessment. As with GRADE, there are four possible levels of confidence:

- High confidence: It is highly likely that the review finding is a reasonable representation of the phenomenon of interest.
- Moderate confidence: It is likely that the review finding is a reasonable representation of the phenomenon of interest.
- Low confidence: It is possible that the review finding is a reasonable representation of the phenomenon of interest.
- Very low confidence: It is not clear whether the review finding is a reasonable representation of the phenomenon of interest.

The GRADE‐CERQual approach involves an assessment of five factors:

1. Methodological limitations: The extent to which there are concerns about the design or conduct of the primary studies that contributed evidence to an individual review finding
2. Coherence: An assessment of how clear and cogent the fit is between the data from the primary studies and a review finding that synthesises that data.
3. Adequacy of data: An overall determination of the degree of richness and quantity of data supporting a review finding
4. Relevance: The extent to which the body of evidence from the primary studies supporting a review finding is applicable to the context (perspective or population, phenomenon of interest, setting) specified in the review question
5. Dissemination bias: A systematic distortion of the phenomenon of interest due to selective dissemination of studies or individual study findings.

We will present and explain our CERQual ratings in succinct, transparent, and informative Summary of Qualitative Findings tables.

## DECLARATIONS OF INTEREST

There are no reported conflicts of interest on this review. Several of the review authors are involved with the International Development Coordination Group of the Campbell Collaboration. However, the IDCG editor for this review is not involved in the review. The review will be also independently assured by the IDCG's independent co‐chair.

## PRELIMINARY TIMEFRAME

The planned time frame for this review is as follows:

- Draft and Final Protocols: December 2020 to January 2021.
- Draft and Final Report : June to July 2021.

## PLANS FOR UPDATING THIS REVIEW

The search of this study has been completed in February 2021 based on a search strategy developed in December 2020. The core team will explore opportunities for funding an update of this review upon submission of the final report in 2021.

**ADDITIONAL TABLES**

**Additional tables 1:** Table example: insert title here

Bold text, 5% grey background	Table text
Text	No vertical lines, only horizontal
Text	The table may be used with grey background/bold text on top instead of to the left. See Additional Table 2 below
Text	
Text	

**Additional tables 2:** Table example: insert title here

Study	0utcome	Results
Barth 1994	Adoption	1. Initial placement in a kinship home decreases the odds of adoption by 50% (OR = 0.50)
Belanger 2001	Adaptive behaviours psychiatric disorders	1. The interaction of type of placement, home index, and temperament match did not account for more of the variance in VABS and DSMD scores than did type of placement alone
Benedict, Zur 1996	Institutional abuse	1. Placement in foster care increases the likelihood of association with maltreatment by 4.4 times

## Footnotes

1 The team used the following keywords for the literature search: “Gender”, “Women empowerment”, “Adolescent girls’ empowerment”, “gender‐based violence”, “intimate partner violence”.

2 This does not rule out other synthesis approaches such as framework synthesis (Brunton et al., 2020).

3 We will also consider conducting a thematic synthesis to identify relevant factors to feed into the QCA.

## APPENDIX A QUALITATIVE ANALYSIS TOOL

**Table:**

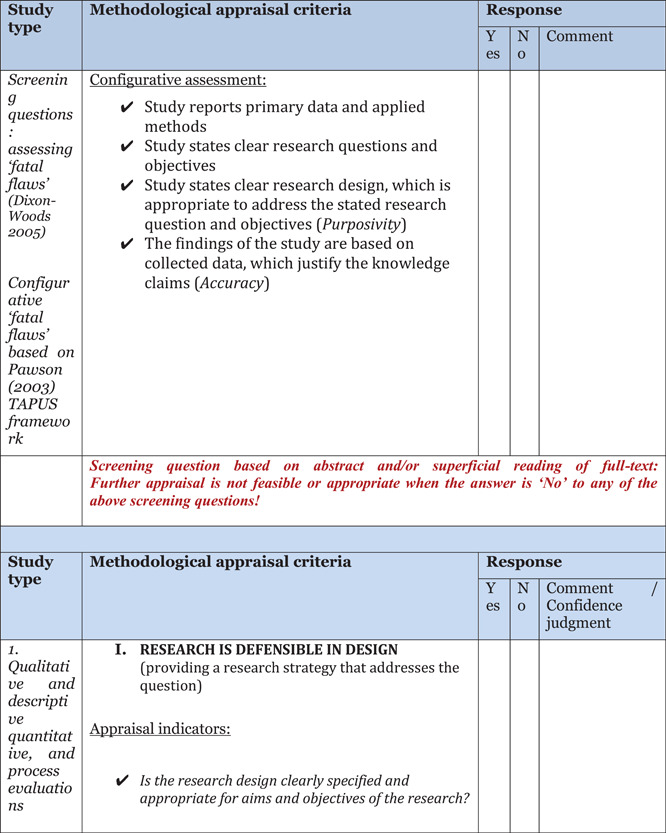

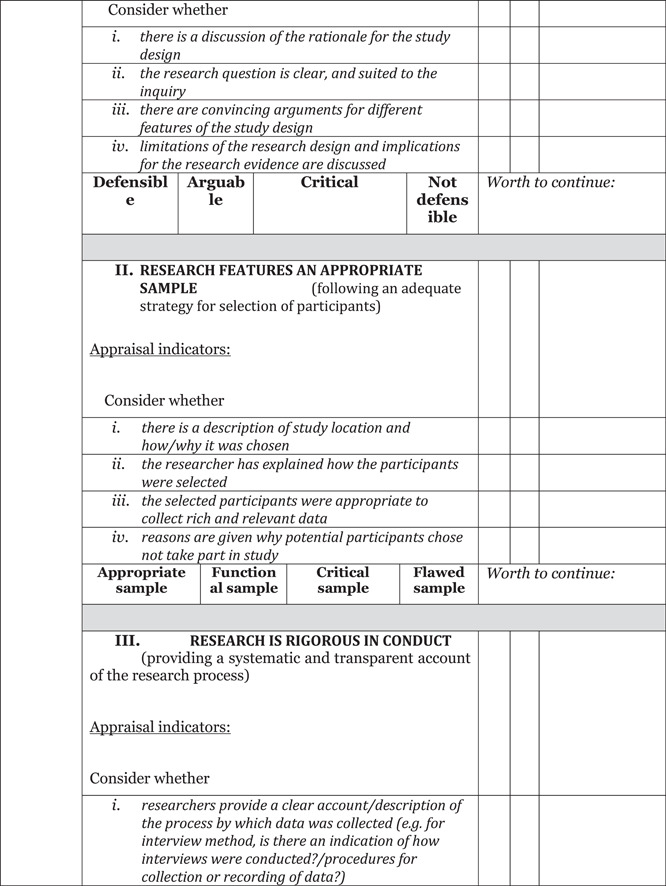

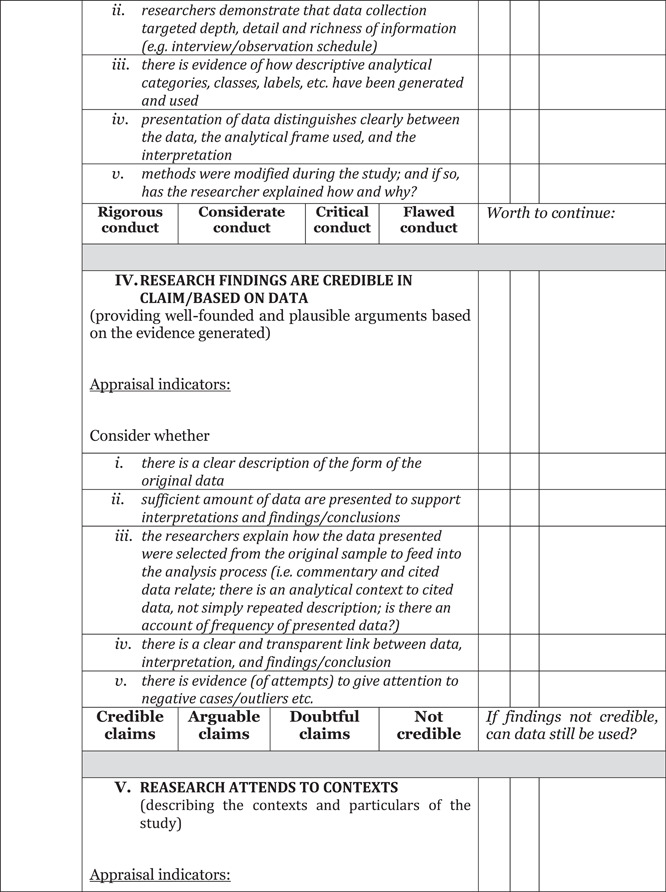

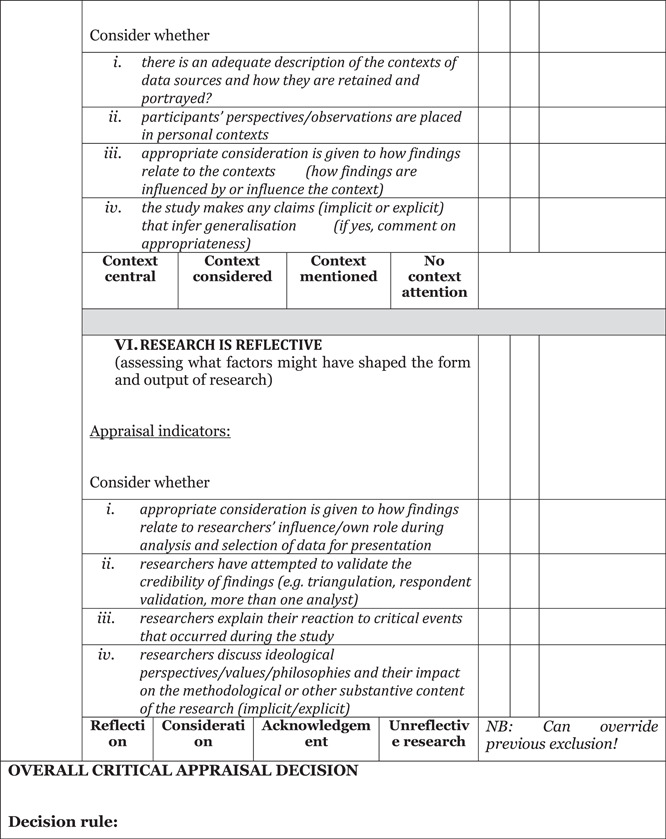

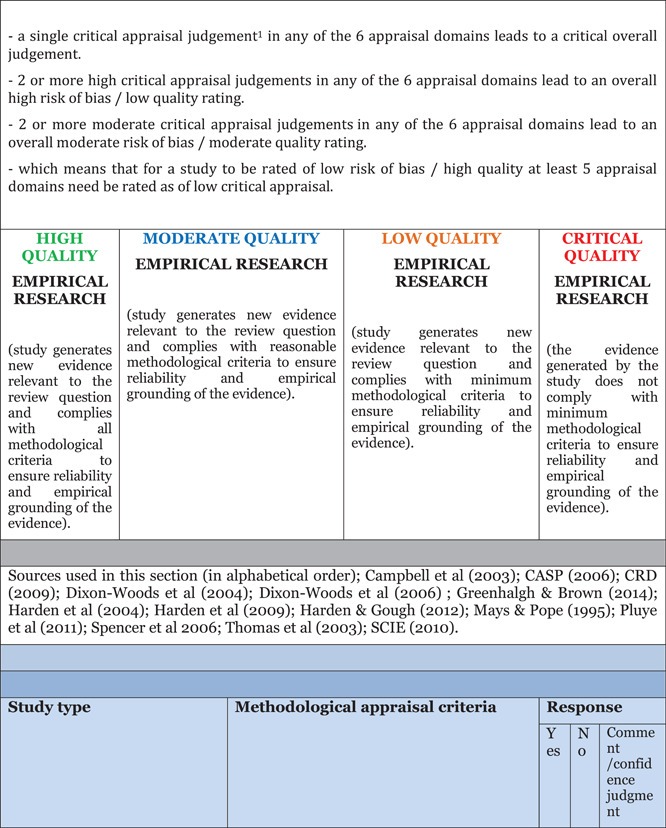

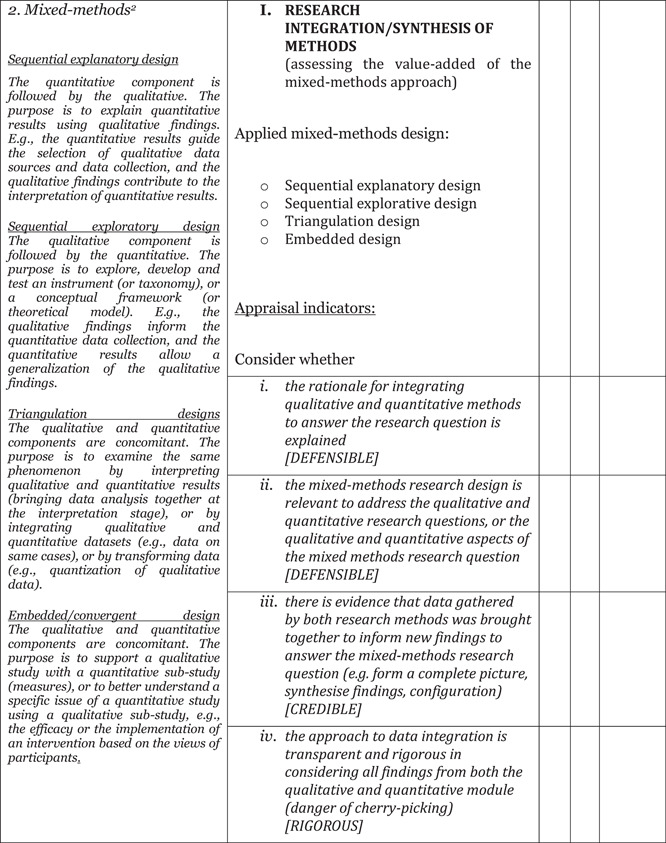

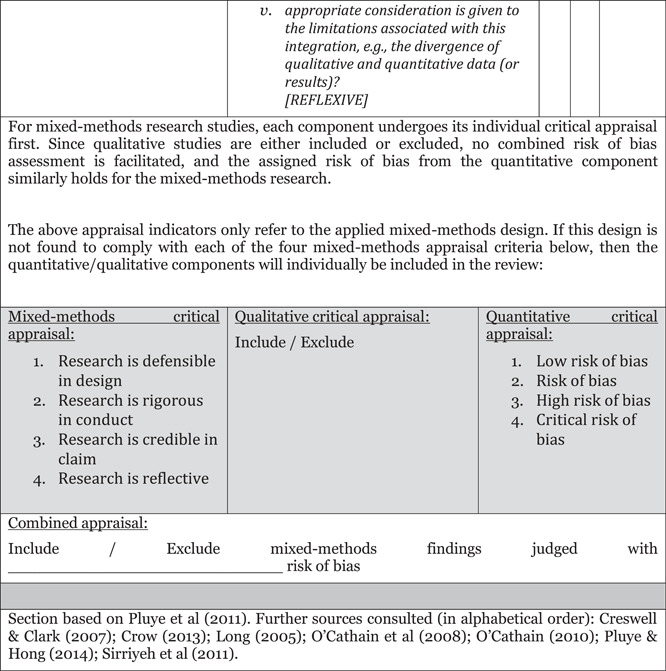

## APPENDIX B DATA EXTRACTION TOOLS

The following tables provide provisional data extraction tools for descriptive, qualitative, and effect size data. If necessary, we could amend these tools to better capture key characteristics of primary studies.

**Table B1:** Provisional tool for descriptive data coding

	Description	Question	Coding instruction
Report identification	Unique study identification #		EPPI ID
		Surname	Surname
		First author surname and type of paper	
	Publication date	Year (letter—if more than one study from that author and that year)	XXXX (a)
	Publication type	What is the impact evaluation publication type?	1 = Peer‐reviewed journal; 2 = Book chapter/book; 3 = Conference paper; 4 = Organisation report; 5 = Working paper; 6 = Implementation document; 7 = other grey; 8 = PhD thesis/dissertation
	Funding agency	Who is funding the evaluation/study?	1= Public institution (e.g., govt, university, research institute); 2= Private institution (e.g., private company); 3= Multilateral Organisation (World Bank, UN); 4 = Foundations; 5 = NGO; 8= Not clear; 9= Not applicable (nonfunded)
	Name of funding agency	Please add name of the agency funding the evaluation	Open answer
	Independence of evaluation	What level of independence is there between the implementing agency and study team?	1=Funding and author team independent of implementers/funders of programme; 2=Funding independent of implementers/funders of programme, but includes authors from funder/implementer; 3=Evaluation funded and undertaken by funders/implementers; 8=Unclear
	Independent data collection	Has the data been collected by an independent party?	1= Yes; 2=No; 8=Not clear
	Conflict of interest	Is there a potential conflict of interest associated with study which could influence results collected/reported? (e.g., Is there a declaration of conflict of interest? Is any of the authors related in any way to the funding or implementing institution?)	1=Yes; 2=No; 8=Not clear
	Comments on conflict of interest	Please add reason for your answer to whether there is a conflict of interest	Open answer
	Language of publication	Language of publication of the impact evaluation, for example, Spanish, English and so forth	Open answer
	Other methods	If the impact evaluation addresses other questions than effectiveness note questions and methods used here	Open answer (this will include for example mixed‐methods to assess implementation, adherence, participant views, etc.)
	Linked studies	If there is any study linked to this one add the reference in APA7	Open answer (in APA7th)
Context	Country	List countries the study was conducted in	Country 1, Country 2, and so forth
	Detailed location	If provided, give detailed information on where the study took place within a country, for example regions/districts covered	Open answer
	GII	GII score on the 1st year of implementation	Open answer
	FSI	FSI score on the 1st year of implementation	Open answer
	WB FCAS	Listed in this donor list of FCAS on the 1st year of implementation?	1=Yes; 2=No; 8=Not clear
	OECD FCAS	Listed in this donor list of FCAS on the 1st year of implementation?	1=Yes; 2=No; 8=Not clear
	Fragility Description	Brief description of the conflict/key players/causes and whether this creates a key bottleneck for women's empowerment, and ultimately peace	Open answer
	WPS	(If post 2016‐ WPS Score on 1st year implementation?)	Open answer
	World Bank Region	Select region(s) the study was conducted according to the World Bank. For more info on region classification see http://data.worldbank.org/country	1= East Asia & Pacific; 2= Europe & Central Asia; 3= Latin America & Caribbean; 4= Middle East & North Africa; 5=South Asia; 6=Sub‐Saharan Africa
	WB Income category	Select the World Bank income classification of the country at the time of the study	1 = Low income country; 2 = Lower‐middle income country; 3 = Upper‐middle income country
	Programme or project name	State the programme or project name. If no name, then list the location	Open answer
Intervention descriptives	Intervention type	Select the intervention type	1=Civil society, associations and networks
			2=Economic rights and entitlements
			3=Formal education
			4=Non‐formal education
			5=TVET
			6=MSMEs
			7=Cash‐based approaches to support women's access to and participation in education and/or the economy
			9=Financial inclusion
			10=Community and leisure activities
			11=Civic education and leadership
			12=Voice and participation in local and subnational governance and development bodies
			13=Legal rights education
			14=Behaviour change communication around support for women's rights and preventing SGBV
			15=Preventative protection measures
			16=Capacity building and technical support to subnational government officials to strengthen service provision for women and gender equality
			17=Gender‐sensitive policing
			18=Informal judicial system
			19=Conflict early warning systems
			20=Dialogue groups
			21=Community consultations
			22=Capacity building for conflict transformation
			23=Peace education
			24=(Re)integration of forcibly displaced populations
			25=Disaster risk reduction
			26=Disarmament, demobilisation and reintegration (DDR)
			27 = Economic support, asset transfers and livelihoods
			8= Other
	Intervention type	Write a short paragraph to describe the intervention type and characteristics. The description should be as detailed as possible. Add page numbers	Open answer
	Ecological level	Select the ecological level(s) applicable to this study	1= Individual; 2= household; 3= community; 4= other
	Ecological level	Write a short paragraph to describe the ecological level and characteristics. The description should be as detailed as possible. Add page numbers	Open answer
	Pillar type UNSCR 1325	Select the UNSCR 1325 pillar(s) applicable to this study	1= participation; 2= prevention; 3= protection; 4= recovery and relief
	Pillar type UNSCR 1325	Write a short paragraph to describe the intervention type and characteristics. The description should be as detailed as possible. Add page numbers	Open answer
	Objectives of intervention	State any objectives stated in study or other document	Open answer
	Intervention development	To what extent is the intervention locally developed/demanded or donor created? Please present any information presented in the paper(s), N/A if no information presented	Open answer
	Intervention implementing agency	Who is implementing the intervention? State the name (and department) of the implementing agency	Open answer
	Implementation funding agency	Type of funder for the implementation of the intervention	1=Government; 2=NGO; 3=Multilateral/bilateral organisation; 4= Foundation; 5= Private sector; 6= Other; 8= Unclear
	Intervention funding agency	Name of intervention funding agency	Open answer
	Intervention target group	What were the characteristics of beneficiaries used to target the intervention?	Open answer
	Intervention target group	Select the target intervention group	1= Girls; 2= Boys; 3= Women; 4= Men; 5 = non‐binary; 8= Unclear
	Targeting methods	How were beneficiaries targeted for the programme (e.g.: how was the targeting implemented)? Page number	Open answer
	Intervention start	Start date (if not stated, state study date) of intervention	XX/XXXX
	Intervention end	State end date (if ongoing state ongoing)	XX/XXXX
	Intervention length	Start intervention length (months)	State number of months
	Consideration of equity	Does the study consider equity?	1=Yes; 2=No
	Equity methods	How does the study consider equity?	1. Does not address gender or equity
			2. Vulnerable population targeted
			3. Subgroup analysis by sex
			4. Subgroup analysis (other than sex)
			5. Equity sensitive analytical framework
			6. Equity sensitive methodology
			7. Equity sensitive research process
			8. Measures effects on an inequality outcome
			9. Ethics approval referenced
			10. Ethics informed by equity
	Equity dimension	What dimension(s) of equity does the study consider? PROGRESS + indicators (multiple choice—may pick more than one)	1. Age (e.g., old or young age but only if it provides arguments)
			2. Conflict‐affected
			3. Culture (includes language)
			4. Disability (medical, physical, neurological, mental disorders)
			5. Education
			6. Ethnicity
			7. Head of household (female or male)
			8. HIV/AIDS (people with or at risk of HIV)
			9. Land size
			10. Land ownership
			11. Place of residence (rural, urban, peri‐urban, informal dwellings)
			12. Refugees
			13. Religion
			14. Socioeconomic status (income or poverty status)
			15. Social capital
			16. Sex (includes the use of the term gender meaning the biological sex of a person)
			17. Sexual orientation
			18. Sexual identity
			19. Other (vulnerable groups not typified by any of the above). Could include: orphans, sex workers, survivors of sexual violence, etc.)
			20. Not applicable
Process and implementation	Information about programme take‐up	Is there any information about programme take‐up?	1=Yes, commentary from author; 2= Yes, formally assessed; 3=No
		Commentary by authors should be used when information on programme take/up, and so forth, is not backed up by some sort of research/when the authors do not report that/how they collected data to assess these areas	
	Methods of assessing take‐up	Which methods are used to assess programme take‐up?	1= Observation by intervention staff; 2= Reporting by participants; 3= Other; 4= Commentary from author; 9= Not measured
	Results of the assessment of take‐up	What is the result/information provided of the assessment of programme take‐up?	Open answer
	Information about programme adherence (among beneficiaries)	Is there any information about programme adherence (among beneficiaries)?	1=Yes, commentary from author; 2= Yes, formally assessed; 3=No
		Commentary by authors should be used when information on programme adherence, and so forth, is not backed up by some sort of research/when the authors do not report that/how they collected data to assess these areas	
	Methods of assessing adherence	Which methods are used to assess programme adherence for beneficiaries? This includes attrition and dropout rates, adherence to appointments, and so forth	1= Observation by intervention staff; 2= Reporting by participants; 3= Other; 4= Commentary from author; 9= Not measured
	Results of the assessment of adherence	What is the result/information provided of the assessment of programme adherence?	Open answer
	Information about implementation fidelity/intervention delivery quality (among implementers)	Is there any information on implementation fidelity/intervention delivery quality?	1=Yes, commentary from author; 2= Yes, formally assessed; 3=No
		Commentary by authors should be used when information on programme adherence, and so forth, is not backed up by some sort of research/when the authors do not report that/how they collected data to assess these areas	
	Methods of assessing intervention fidelity	Which methods are used to assess implementation fidelity/intervention delivery quality by the implementing partner	1= Observation by intervention staff; 2= Reporting by participants; 3= Other; 4= Commentary from author; 9= Not measured
	Results of the assessment of intervention fidelity	What is the result/information provided of the assessment of implementation fidelity/intervention delivery quality	Open answer
	Frequency of contact	What is the frequency of contact between beneficiaries and provider or treatment activity?	1= Daily; 2=Weekly; 3=Monthly/Multi‐months; 4=Annually; 8= unclear
	Incentives	Were incentives provided to intervention participants?	1=Yes; 2=No; 8=Not clear
	Other description of process/implementation factors	Any other description of process/implementation factors not covered above	Open answer
	Causal mechanisms/barriers and enablers	Does the study identify any causal mechanisms/barriers and enablers related to context (not included above)?	1=Yes, commentary from author; 2= Yes, formally assessed; 3=No
	Methods	How are these identified?	1= Observation by intervention staff; 2= Reporting by participants; 3= Other; 4= Commentary from author; 9= Not measured
	Results	Report here any material relevant to causal mechanisms and barriers and enablers	Open answer
	Cost	Are any unit cost data/cost‐effectiveness estimates provided?	1=Yes; 2=No
	Cost details	If yes, report any details of unit cost and/or total cost. Please also report the year and currency.	Open answer
External validity	Length of study	Length of study in months (Where study length not reported, code as length of intervention, noting that in brackets)	# months, if not reported N/A
	Efficacy or effectiveness trial	Was the intervention implemented under "real world" conditions? By real world we mean a programme implemented independently of the evaluation, either by government, NGO or international agency	1=Yes; 2=No; 9= N/A
	Personnel implementing the programme	Who was in charge of implementing the programme?	1=PI/researchers (study authors); 2= implementing agency staff; 3= external agency (e.g.: survey firm); 4=Others; 8= Not clear
	Sampling frame for the study	State the sampling frame (list of all those within a population who can be sampled, i.e. households, communities) for selection of study participants (i.e. Census, etc.)	Open answer
	Author discussion of external validity	Do the authors discuss or explicitly address generalisability/applicability?	Open answer
	Programme theory	Do the authors make explicit reference to programme theory, theory of change or similar?	1=Yes; 2=No; 8=Not clear
		Report any description/statement of programme theory as stated by author(s)	Open answer
		Is the study using theory to inform the evaluation design and analysis?	Open answer—describe if and how the authors use theory in the evaluation. Do they for example use it to inform data collection? Do they do any causal chain analysis? Do they refer to international index on Gender (GDI, GII, etc.)? Do they refer to international index on peace (WPS, GPI, FSI, World Bank/OECD FCAS list etc.)
	COMMENTS	Any comments, decision of exclusion (and justification)	X

**Table B2:** Provisional tool for effect size data coding

	Description	Question	Coding
ID	Unique study identification #		For example, 12345678
	Unique effect size identification #		For example, 12345678_1, 12345678_2, 12345678_3, and so forth
	First author—impact evaluation	Surname	For 1 author: leading author last name (e.g., Gomez)
			For 2 authors: both author last names with ampersand in between (e.g., Smith & Bahn)
			For 3 or more authors: leading author last name followed by et al. (e.g., Gupta et al.)
Outcome for effect size (answer for all studies)	Definition of outcome	Please provide the authors definition of the outcome (including description of the subgroup if relevant)	Open answer
	Exposure to intervention (in months)	How long is the intervention exposure itself?	#
	Evaluation period (in months)	The total number of months elapsed between the end of the intervention and the point at which an outcome measure is taken post intervention, or as a follow‐up measurement. If <1 month, use decimals (e.g., 1 week would be 0.25)	#
	Comparison	What type of comparison group is used?	1=No intervention (service delivery as usual)
			2=Other intervention
			3=Pipeline (wait‐list) control (still service intervention the delivery as usual)
	Describe comparison group	If answer above is (1) no intervention, type N/A, if (2) Other Intervention, list what control group is receiving, if (3) Pipeline control, report when the control group will receive the intervention in relation to the treatment group (e.g., 1 year later)	Free text
	Counterfactual	How is the counterfactual chosen?	Free text (e.g., random control trial, propensity score matching, etc.)—Multiple codes are ok
	Subgroup analysis	Is this effect size data for a subgroup?	0 = No
			1 = Yes
	Subgroup analysis description	If yes to question 2, which type of subgroup?	Open answer—this can include separate samples for gender, income, place of residence
	Source	Which page(s) contain the effect size data?	Open answer
	Data to be extracted	Which type of data to be extracted?	1 = Continuous—means and SDs
			2 = Continuous—mean difference and SD
			3 = Dichotomous outcome—proportions
			4 = Regression data—dichotomous outcome (e.g., logistic regression)
			5 = Regression data—continuous outcome (e.g., linear regression)
	Analysis type for this effect size	What type of analysis was used (Regression, 2SLS, ANCOVA, etc.)? Multiple codes are ok	Free text
Effect size data (answer for all studies)	Sample size metric	Sample size unit of analysis	1= Individual
			2= Household
			3= Group (e.g., community org)
			4= Village
			5 = Other
			6 = Not clear
	Treatment effect estimated	What treatment effect is estimated?	1=ITT
			2=ATET
			3=ATE
			4=LATE
	Sample size (treatment)	Initial sample size treatment group	#
	Sample size (control)	Initial sample size control group	#
	Sample size (total)	Initial sample size total	#
	Observations (treatment)	Number of treatment observations after attrition/follow up	#
	Observations (control)	Number of control observations after attrition/follow up	#
	Observations (total)	Total number of control observations after attrition/follow up	#
Outcome data—if continuous (Means and SDs)	Baseline outcome treatment	State result of baseline outcome for treatment group	#
	SD baseline outcome treatment	State SD of baseline outcome measure for treatment group	#
	Baseline outcome control	State result of baseline outcome for control group	#
	SD baseline outcome control	State SD of baseline outcome measure for control group	#
	Outcome in treatment post intervention	State result of post intervention outcome for treatment group	#
	SD outcome in treatment post intervention	State SD of post intervention outcome measure for treatment group	#
	Outcome in control post intervention	State result of post intervention outcome for control group	#
	SD outcome in control post intervention	State SD of post intervention outcome measure for control group	#
	Outcome in treatment 1st follow up	State result of 1st follow up outcome measure for treatment group	#
	SD outcome in treatment 1st follow up	State SD 1st follow up outcome measure for treatment group	#
	Outcome in control 1st follow up	State result of 1st follow up outcome measure for treatment group	#
	SD outcome in control 1st follow up	State SD 1st follow up outcome measure for treatment group	#
Outcome data—If continuous (Mean difference and SD/SE at follow up)	Mean difference at follow up	State mean difference	#
	SD at follow up	State SD at follow up	#
	SE	State SE	#
Outcomes data—if dichotomous (Proportions r)	Odds ratio	State reported odds ratio (or calculated odds ratio if a frequency table is reported)	#
Regression data	Coefficient	What is the coefficient estimate?	#
	Pooled *SD* of outcome	What is the pooled standard deviation of the outcome?	#
	*SE*	What is the standard error of the coefficient estimate?	#
	*t* test	What is the *t* statistic associated with the focal predictor?	#
	*p* value	What is the *p* value associated with the coded effect?	#

## APPENDIX C RISK OF BIAS ASSESSMENT TOOL

The following table provides a provisional tool to guide the risk of bias assessment for quantitative impact evaluations. If necessary, we could amend the tool to better inform the appraisal of primary studies.

**Provisional risk of bias assessment tool (RCT)**

**Table:**

General	ID	EPPI ID		
General	Study first author	Open answer		
General	Time taken to complete assessment	Minutes		
General	Design type: What type of study design is used?	1= RCT (random assignment to households/individuals) or quasi‐RCT	‐	
		2= Cluster‐RCT (quasi‐RCT)	
General	Methods used for analysis: Which methods are used to control for selection bias and confounding?	1 = Statistical matching (PSM, CEM, covariate matching)	‐	
		2 = Difference in differences (DID) estimation methods		
		3 = IV‐regression (2‐stage least squares or bivariate probit)		
		4 = Heckman selection model		
		5 = Fixed effects regression		
		6 = Covariate adjusted estimation		
		7 = Propensity weighted regression		
		8 = Comparison of means		
		9 = Other (please state)		
General	Design and analysis method description	Open answer	Briefly describe the study design and analysis method undertaken by the authors	
General	Study population	Open answer	Provide any details in the paper that describe how the study population was selected, covering:	
			(a) How is the population selected? what is the sampling strategy to recruit participants from that population into the study?	
			(b) What are the characteristics of that study participants?	
			(c) Was this a pilot program aimed at being scaled up?	
			(d) Were there specific factors of success or failure in the implementation?	
General	Type of comparison group	1=No intervention (service delivery as usual)	Indicate type of comparison group	
		2=Other intervention	
		3=Pipeline (wait‐list) control (still service delivery as usual)	
General	Type of comparison group (if other)	Open answer		
General	Ethical clearance	Open answer	Provide any details of ethical research clearances granted. Report unclear if this information is not available	
General	Study registration	Open answer	Provide any detials of study registration, inlcuding registry IDs, and so forth	
1: Assignment mechanism—Assessment	Assignment mechanism: Was the allocation or identification mechanism random or as good as random?	1= Yes, 2 = Probably Yes, 3 = Probably No, 4 = No, 8 = Unclear	(a) The authors describe a random component in sequence generation/randomization method (e.g., lottery, coin toss, random number generator) and assignment is performed for all units at the start of the study centrally or using a method concealed from participants and intervention delivery	Score “Yes” if all criterion (a), (b), (c) and (d) are satisfied
				Score "Probably Yes" if only criterion (a) and (b) are not satisfied OR if only criteria (c) is not satisfied
				Score “Unclear” if (d) is not satisfied because no balance table is reported
			(b) If public lottery is used for the sequence generation, authors provide detail on the exact settings and participants attending the lottery	Score "Probably No" if (d) is not satisfied because there is no balance table reported and there is evidence suggesting a problem in the randomization, such as baseline coefficients in a diff‐in‐diff regression table are very different or sample size is too small for the procedure used (using stratification when there are less than two units for each intervention and control group in each strata can lead to imbalance)
			(c) If a special randomization procedure is used to ensure balance, it is well described and justified given the study setting (stratification, pairwise matching, unique random draw, multiple random draws, etc.)	
			(d) A balance table is reported suggesting that allocation was random between all groups including subgroup receiving different treatment within control or treatment groups (if the comparison is relevant for this assessment)	Score “No” if (d) is not satisfied because there are large imbalances concerning a large number of variables, providing evidence that the assignment was not random. If this is scored as no, use the NRS tool
1: Assignment mechanism—Justification	Assignment justification	Open answer	Justification for coding decision (include a brief summary of justification for rating, mentioning your response to all sub questions, cite relevant pages)	
2: Unit of analysis—Assessment	Unit of analysis: Is unit of analysis in cluster allocation addressed in *SE* calculation?	1=Yes, 2=No, 3=Not reported/unclear, 4=Not applicable	Score “Yes" if UoA = UoR OR if UoA != UoR and *SE*s are clustered at the UoR level OR data is collapsed to the UoR level	
			Score "Not reported/unclear" if not enough information is provided on the way the *SE*s were calculated or what the unit of analysis is
			Score “Not applicable” if it is not a cluster RCT
			Score “No” otherwise
2: Unit of analysis—Justification	Method used to address differences between UoA and unit of data collection	Open answer		
3: Selection bias—Assessment	Selection bias Was any differential selection into or out of the study (attrition bias) adequately resolved?	1= Yes, 2 = Probably Yes, 3 = Probably No, 4 = No, 8 = Unclear	Score “Yes" if there is no attrition or attrition falls into the green zone and the study establishes that attrition is randomly distributed (e.g., by presenting balance by key characteristics across groups) AND if survey respondents were randomly sampled	
			Score "Probably yes" if attrition falls into the green zone AND if survey respondents were randomly sampled
			Score "Unclear" if there is an attrition problem but no information provided on the relationship between attrition and treatment status, OR if there is not enough information on how the population surveyed was sampled
			Score "Probably no" if there is attrition which is likely to be related to the intervention OR there is some indication that the survey respondents were purposely sampled in a way that might have led the sampling to be different between treatment and control groups, or attrition falls into the yellow zone
			Score "No" if attrition falls into the red zone
3: Selection bias—Justification	Selection bias justification	Open answer	Justification for coding decision (Include a brief summary of justification for rating, mentioning your response to all sub questions, cite relevant pages)	
3: Confounding—Assessment	Confounding and group equivalence: Was the method of analysis executed adequately to ensure comparability of groups throughout the study and prevent confounding	1= Yes, 2 = Probably Yes, 3 = Probably No, 4 = No, 8 = Unclear	(a) Baseline characteristics are similar in magnitude;	Score “Yes” if criterion (a) and (b) are satisfied;
			(b) Unbalanced covariates at the individual and cluster level are controlled in adjusted analysis;	Score "Probably yes" if (a) is not satisfied but (b) is satisfied and imbalances are small in magnitude OR if only (a) is satisfied.
			(c) Adjustments to the randomization were taken into account in the analysis (stratum fixed effects, pairwise matching variables)? (Bruhn & McKenzie, 2009)	Score “Unclear” if no balance table is provided or if imbalances are controlled for but they are very large in magnitude and assignment mechanism is not coded as "Yes" or "Probably yes"
				Score "Probably no" if (a) and (b) are not satisfied and the magnitude of imbalances are small
				Score “No” if (a) and (b) are not satisfied and the magnitude of imbalances are large and covariates are clear determinant of the outcomes
3: Confounding—Justification	Confounding justification	Open answer	Justification for coding decision (include a brief summary of justification for rating, mentioning your response to all sub questions, cite relevant pages)	
4: Deviations from intended interventions—Assessment	Deviations from intended interventions: Spill‐overs, cross‐overs and contamination: was the study adequately protected against spill‐overs, cross‐overs and contamination?	1= Yes, 2 = Probably Yes, 3 = Probably No, 4 = No, 8 = Unclear	(a) There was no implementation issues that might have led the control participants to receive the treatment (implementer's mistake)	Score “Yes” if criterion (a), (b), (c) and (d) are satisfied;
			(b) The intervention is unlikely to spill‐over to comparisons (e.g., participants and non‐participants are geographically and/or socially separated from one another and general equilibrium effects are not likely) or the potential effects of spill overs were measured (e.g., variation in the % of unit within a cluster receiving the treatment)	Score "Probably yes" if there is no obvious problem but there is no information reported on potential risks related to spill overs, contamination, or survey effects in the control group OR if there were issues with spill‐overs but they were controlled for or measured
				Score “Unclear” if spill‐overs, cross‐overs, survey effects and/or contamination are not addressed clearly
			(c) There is no risk of contamination by external programs: the treatment and comparisons are isolated from other interventions which might explain changes in outcomes.	Score "Probably no" if any of the criterion (a), (b), (c) or (d) are not satisfied but the scale of the issue is not clear
			(d) There is nothing in the surveys that might have given the control participants an idea of what the other group might receive OR they did but there is no risk that this has changed their behaviours; AND the survey process did not reveal information to the control group that they did not have before (e.g., the study aims to measure increase in take up of a service or product that participants might not know about) Authors might put something in place in the design of the study that allows to control for that survey effect (e.g., a pure control with no monitoring except baseline end line)	Score “No” if any of the criterion (a), (b), (c) or (d) are not satisfied and happened at a large scale in the study
4: Deviations from intended interventions—Justification	Deviations justification	Open answer	Justification for coding decision (Include a brief summary of justification for rating, mentioning your response to all sub questions, cite relevant pages)	
			For example, intervention groups are geographically separated, authors use intention to treat estimation or instrumental variables to account for non‐adherence, and survey questions are not likely to expose individuals in the control group to information about desirable behaviours (“survey effects”)	
5. Performance bias—Assessment	Performance bias: was the process of monitoring individuals unlikely to introduce motivation bias among participants?	1= Yes, 2 = Probably Yes, 3 = Probably No, 4 = No, 8 = Unclear	(a) The authors state explicitly that the process of monitoring the intervention and outcome measurement is blinded and conducted in the same frequency for treatment and control groups, or argue convincingly why it is not likely that being monitored could affect the performance of participants in treatment and comparison groups in different ways (such as resulting in Hawthorne or John Henry effects).	Score “Yes” if either criterion (a) or (b) are satisfied;
				Score "Probably yes" if the study is based on data collected during a trial and there is no obvious issue with the monitoring processes but authors do not mention potential risks
				Score “Unclear” if it is not clear whether the authors use an appropriate method to prevent Hawthorne and John Henry Effects (e.g., blinding of outcomes and, or enumerators, other methods to ensure consistent monitoring across groups). Hawthorne effects may result where participants know that they are being observed and John Henry Effects may result from participant knowledge of being compared
			(b) The outcome is based on data collected in the context of a survey, and not associated with a particular intervention trial, or data are collected from administrative records or in the context of a retrospective (ex post) evaluation	Score "Probably no" if there was imbalance in the frequency of monitoring in intervention groups, which might have influenced participants' behaviours
				Score "No" if neither criterion (a) or (b) are satisfied
5. Performance bias—Justification	Performance bias justification	Open answer	Justification for coding decision (Include a brief summary of justification for rating, mentioning your response to all sub questions, cite relevant pages)	
6. Outcome measurement bias—Assessment	Outcome measurement bias: was the study free from biases in outcome measurement?	1= Yes, 2 = Probably Yes, 3 = Probably No, 4 = No, 8 = Unclear	(a) Outcome assessors are blinded or the outcome measures are not likely to be biased by their judgement	Score “Yes” if criterion (a), (b), (c) and (d) are satisfied:
			(b) For self‐reported outcomes: respondents in the intervention group are not more likely to have accurate answers due to recall bias;	Score "Probably yes" if there is a small risk related to any of (a), (b), (c) or (d) and there is no more information provided to to justify the absence of bias OR if there was a high risk of bias but authors have either controlled it in their design or measured it with a placebo outcomes
			(c) For self‐reported outcomes: respondents do not have incentives to over/under report something related to their performance or actions, OR researchers put in place mechanisms to reduce the risk of reporting bias (researchers not strongly involved in the implementation of the program and it is clear that their answers to the survey will not affect what they receive in the future) OR authors have measured the risks of bias through falsification tests or measuring the effect on placebo outcomes in cases where there was a risk of reporting bias	Score “Unclear” if it there is a high risk related to any of (a), (b), (c) or (d) and there is no more information provided to to justify the absence of bias
				Score "Probably no" if there are high risk related to (a), (b), (c) or (d) and it is clear that authors were not able to control for this bias
				Score “No” if there is evidence of bias
			(d) Timing issue: the data collection period did not differ between intervention and comparison group, the baseline data is not likely to be affected by the beginning of the intervention or affects a small percentage of the study participants	
6. Outcome measurement bias—Justification	Outcome measurement justification	Open answer	Justification for coding decision (Include a brief summary of justification for rating, mentioning your response to all sub questions, cite relevant pages).	
7. Reporting bias—Assessment	Analysis reporting: was the study free from selective analysis reporting?	1= Yes, 2 = Probably Yes, 3 = Probably No, 4 = No, 8 = Unclear	(a) A preanalysis plan or trial protocol is published and referred to or the trial was pre‐registered or the outcomes were pre‐registered;	Score "Yes" if all the criterion (a), (b), (c), (d), and (e) are satisfied;
			(b) Authors report results corresponding to the outcomes announced in the method section (there is no outcome reporting bias);	Score "Probably yes" if all the conditions are met except (a), or if all the conditions are met but there is some element missing that could have helped understand the results better (e);
			(c) Authors report results of unadjusted analysis and intention to treat (ITT) estimation, alongside any adjusted and treatment‐on‐the‐treated/complier‐average‐causal‐effects analysis)	Score "Unclear" if there is not enough information to determine that there is an analysis missing;
			(d) Authors use the appropriate analysis method (use baseline data when available) and different treatment arms are differentiated in the analysis	Score "Probably no" if any of the criterion (b), (c) or (d) are not satisfied;
			(e) Authors have reported all the analysis which could help understand the results and no other bias is assessed as unclear due to the lack of an important analysis (e.g., a balance table or a subgroup analysis)	Score "No" if any of the criterion (b), (c) or (d) are not satisfied and there is evidence that the analysis results would be different because large imbalances were not controlled for, compliance was very low and ITT estimation was not reported or different treatment arms were pooled
8. Reporting bias—Justification	Analysis reporting justification	Open answer	Justification for coding decision (Include a brief summary of justification for rating, mentioning your response to all sub questions, cite relevant pages)	
9. Other bias—Assessment	Other risks of bias Is the study free from other sources of bias?	1= Yes, 4 = No		
9. Other bias—Justification	Other bias justification	Open answer	Justification for coding decision (Include a brief summary of justification for rating, mentioning your response to all sub questions, cite relevant pages). For example, information is collected using a different survey instrument in different intervention groups; measurement of the intervention received in unclear	
10. Blinding—observers—Assessment	Blinding of participants?	1=Yes, 2=No, 8=unclear, 9= N/A	If there is no information, code NO. If there is information but it is ambiguous, code UNCLEAR	
10. Blinding—observers—Assessment	Blinding of outcome assessors?	1=Yes, 2=No, 8=unclear, 9= N/A	If there is no information, code NO. If there is information but it is ambiguous, code UNCLEAR	
10. Blinding—analysts—Assessment	Blinding of data analysts?	1=Yes, 2=No, 8=unclear, 9= N/A	If there is no information, code NO. If there is information but it is ambiguous, code UNCLEAR	
10. Blinding—method(s)	Method(s) used to blind	Open answer (including describe method of placebo control), No 9= N/A	Describe method(s) used to blind	
11. External validity—Assessment	External validity	Open answer	a) What do authors say about external validity?	Include all information that can help assess the external validity of the results

**Provisional risk of bias assessment tool (QED)**

**Table:**

Code	Question	Coding	Criteria	Decision‐rules
General	ID	EPPI ID		
General	Time taken to complete assessment	Minutes		
General	Study first author	Open answer	
General	Outcome	Open answer		
General	Study design: What type of study design is used?	1= Natural experiment: randomised or as‐if randomised 2= Natural experiment: regression discontinuity (RD) 3= CBA (non‐randomised assignment with treatment and contemporaneous comparison group, baseline and end line data collection)—individual repeated measurement 4= CBA pseudo panel (repeated measurement for groups but different individuals) 5= Interrupted time series (with or without contemporaneous control group) 6= Panel data, but no baseline (pre‐test) 7 = Comparison group with end line data only		
General	Methods used for analysis: Which methods are used to control for selection bias and confounding?	1 = Statistical matching (PSM, CEM, covariate matching) 2 = Difference in differences (DID) estimation methods 3 = IV‐regression (2‐stage least squares or bivariate probit) 4 = Heckman selection model 5 = Fixed effects regression 6 = Covariate adjusted estimation 7 = Propensity weighted regression 8 = Comparison of means 9 = Other (please state)	‐	
General	Study population	Open answer	Provide any details in the paper that describe how the study population was selected, covering: a) How is the population selected? what is the sampling strategy to recruit participants from that population into the study? b) What are the characteristics of that study participants? c) Was this a pilot program aimed at being scaled up? d) Were there specific factors of success or failure in the implementation?	
General	Ethical clearance	Open answer	Provide any details of ethical research clearances granted. Report unclear if this information is not available.	
General	Study registration	Open answer	Provide any details of study registration, including registry IDs, and so forth	
1: Selection bias—Assessment	1. Mechanism of assignment: was the allocation or identification mechanism able to control for selection bias?	1= Yes, 2 = Probably Yes, 3 = Probably No, 4 = No, 8 = Unclear		
1: Selection bias—Justification	For regression discontinuity designs	Open answer	a) Allocation is made based on a pre‐determined discontinuity on a continuous variable (regression discontinuity design) and blinded to participants or; b) if not blinded, individuals reasonably cannot affect the assignment variable in response to knowledge of the participation decision rule; c) and the sample size immediately at both sides of the cut‐off point is sufficiently large to equate groups on average.	Score “Yes” if criteria a), b), c) are all satisfied Score "Probably Yes" if there are minor differences in between both sides of the cut‐off point but authors convincingly argue that the differences are unlikely to affect the outcome, OR individuals are not blinded and there are low risk of them affecting the assignment but the authors do not mention it. Score “Unclear” if it is unclear whether participants can affect it in response to knowledge of the allocation mechanism. Score "Probably No" if there are differences between individuals on both sides of the cut‐off point, and there are doubts that the differences are due to individuals altering the assignment OR the participants are blinded but there is evidence that the decisions that determined the discontinuity is based on differences between the two groups or differences in time. Score “No” if the sample size is not sufficient OR there is evidence that participants altered the assignment variable prior to assignment. If the research has serious concerns with the validity of the assignment process or the group equivalence completely fails, we recommend assessing risk of bias of the study using the relevant questions for the appropriate methods of analysis (cross‐sectional regressions, difference‐in‐difference, etc.) rather than the RDDs questions.
1: Selection bias—Justification	For assignment based non‐randomised programme placement and self‐selection (studies using a matching strategy or regression analysis, excluding IV)	Open answer	a) Participants and non‐participants are either matched based on all relevant characteristics explaining participation and outcomes, or; b) all relevant characteristics are accounted for.** c) and the data set used contains relevant variable that are measured in a relevant way (i.e. they were not collected for a different purpose initially and therefore are good proxy for some characteristics). **Accounting for and matching on all relevant characteristics is usually only feasible when the programme allocation rule is known and there are no errors of targeting. It is unlikely that studies not based on randomisation or regression discontinuity can score “YES” on this criterion. There are different ways in which covariates can be taken into account. Differences across groups in observable characteristics can be taken into account as covariates in the framework of a regression analysis or can be assessed by testing equality of means between groups. Differences in unobservable characteristics can be taken into account through the use of instrumental variables (see also question 1.d) or proxy variables in the framework of a regression analysis, or using a fixed effects or difference‐in‐differences model if the only characteristics which are unobserved are time‐invariant	Score “Yes” if a) or b) and c) are satisfied Score "Probably yes" if a) or b) are addressed for but there is some doubt related to c), OR authors combined statistical matching and difference‐in‐difference to cope with unobservable differences, OR they only did statistical matching and there was clear rules for selection into the program (no self‐selection). Score “Unclear” if · it is not clear whether all relevant characteristics (only relevant time varying characteristics in the case of panel data regressions) are controlled. Score "Probably no" if only a statistical matching was done and there was self‐selection into the program. Score “No” if relevant characteristics are omitted from the analysis.
1: Selection bias—Justification	For identification based on an instrumental variable (IV estimation)	Open answer	Score “Yes” if an appropriate instrumental variable is used which is exogenously generated: for example, due to a “natural” experiment or random allocation. Score "Probably yes" if there is less evidence (no balance table showing differences between the intervention and comparison group). Score “Unclear” if the exogeneity of the instrument is unclear (both externally as well as why the variable should not enter by itself in the outcome equation). Score "Probably no" if there is evidence that enrolment in the program is correlated with a variable that might also have an effect on outcome and on the instrumental variable. Score “No” if it is clear that the instrument is not exogenous and affect the outcome through other channels than the program.	
2: Confounding—Assessment	2. Group equivalence: was the method of analysis executed adequately to ensure comparability of groups throughout the study and prevent confounding?	1= Yes, 2 = Probably Yes, 3 = Probably No, 4 = No, 8 = Unclear		
2: Confounding—Justification	For regression discontinuity design	Open answer	a) The interval for selection of treatment and control group is reasonably small OR authors have weighted the matches on their distance to the cut‐off point; b) and the mean of the covariates of the individuals immediately at both sides of the cut‐off point (selected sample of participants and non‐participants) are overall not statistically different based on t‐test or ANOVA for equality of means; c) Significant differences in covariates of the individuals have been controlled in multivariate analysis; and for cluster‐assignment, authors control for external cluster‐level factors that might confound the impact of the programme.	Score "Yes, if criterion a), b), c) and d) are addressed. Score "Probably yes" if b) is not addressed but c) is addressed and differences in means are not large. Score “Unclear” if insufficient details are provided on controls; or if insufficient details are provided on cluster controls. Score "Probably no" if b) is not addressed (absence of a difference test or balance table) and there are doubt regarding the continuity on both sides of the cut‐off point (a). Score “No” otherwise.
2: Confounding—Justification	For non‐randomised trials using difference‐in‐differences methods of analysis	Open answer	a) The authors use a difference‐in‐differences (or fixed effects) multivariate estimation method; b) the authors control for a comprehensive set of individual time‐varying characteristics, and for cluster‐assignment, authors control for external cluster‐level factors that might confound the impact of the programme**; c) and the attrition rate is sufficiently low and similar in treatment and control, or the study assesses that drop‐outs are random draws from the sample (for example, by examining correlation with determinants of outcomes, in both treatment and comparison groups); **Knowing allocation rules for the programme—or even whether the non‐participants were individuals that refused to participate in the programme, as opposed to individuals that were not given the opportunity to participate in the programme—can help in the assessment of whether the covariates accounted for in the regression capture all the relevant characteristics that explain differences between treatment and comparison	Score "Yes, if a, b, c, d (if relevant) are addressed and baseline imbalances between groups were relatively low OR the method was combined by a statistical matching. Score "Probably yes" if all possible variables are controlled for and the selection into the program was done according to clear rules, but baseline imbalances between groups were very large. Score “Unclear” if insufficient details are provided; or if insufficient details are provided on cluster controls. Score "Probably no" if some time‐varying characteristics are not controlled for and the program was self‐selected by the intervention groups. Score “No” if any of the criterion is not addressed.
2: Confounding—Justification	For statistical matching studies including propensity scores (PSM) and covariate matching** **Matching strategies are sometimes complemented with difference‐in‐difference regression estimation methods. This combination approach is superior since it only uses in the estimation the common support region of the sample size, reducing the likelihood of existence of time‐variant unobservable differences across groups affecting outcome of interest and removing biases arising from time‐invariant unobservable characteristics.	Open answer	a) Matching is either on baseline characteristics or time‐invariant characteristics which cannot be affected by participation in the programme; and the variables used to match are relevant (for example, demographic and socio‐economic factors) to explain both participation and the outcome (so that there can be no evident differences across groups in variables that might explain outcomes); and, for cluster‐assignment, authors control for external cluster‐level factors that might confound the impact of the programme b) in addition, for PSM Rosenbaum's test suggests the results are not sensitive to the existence of hidden bias; c) and, with the exception of Kernel matching, the means of the individual covariates are equated for treatment and comparison groups after matching; d) different matching methods including varying sample sizes yields the same results and authors take into account the use of control observations multiple times against the same treatment in their *SE* calculation.	Score "Yes, if a, b, c, and d (if relevant) are addressed. Score "Probably yes" if the selection into the program was done according to clear rules, which are used for the matching but there are slight imbalances remaining after matching. Score “Unclear” if relevant variables are not included in the matching equation, or if matching is based on characteristics collected at end line; or if insufficient details are provided on cluster controls. Score "Probably no" if the program was self‐selected by the intervention groups or participants OR if the selection into the program was done according to clear rules but there is no baseline data available to match the participants or groups on. Score “No” if matching was done based on variables that are likely to be affected by the program or any other scenario that affect a), b) c) or d).
2: Confounding—Justification	For regression‐based studies using cross sectional data (excluding IV)	Open answer	a) The study controls for relevant confounders that may be correlated with both participation and explain outcomes (for example, demographic and socio‐economic factors at individual and community level) using multivariate methods with appropriate proxies for unobservable covariates, and, for cluster‐assignment, authors control particularly for external cluster‐level factors that might confound the impact of the programme; b) and a Hausman test with an appropriate instrument suggests there is no evidence of endogeneity**; c) and none of the covariate controls can be affected by participation; d) and either, only those observations in the region of common support for participants and non‐participants in terms of covariates are used, or the distributions of covariates are balanced for the entire sample population across groups; **The Hausman test explores endogeneity in the framework of regression by comparing whether the OLS and the IV approaches yield significantly different estimations. However, it plays a different role in the different methods of analysis. While in the OLS regression framework the Hausman test mainly explores endogeneity and therefore is related with the validity of the method, in IV approaches it explores whether the author has chosen the best available strategy for addressing causal attribution (since in the absence of endogeneity OLS yields more precise estimators) and therefore is more related with analysis reporting bias.	Score "Yes, if a, b, c and d are addressed. Score "Probably yes" if all criterion are addressed but authors did not report the Hausman test (b). Score “Unclear” if relevant confounders are controlled but appropriate proxy variables or statistical tests are not reported; or if insufficient details are provided on cluster controls. Score "Probably no" if any of the criterion other than b) is not addressed. Score “No" if none of the criterion are addressed.
2: Confounding—Justification	For identification based on an instrumental variable (IV estimation)	Open answer	a) The instrumenting equation is significant at the level of F ≥ 10 (or if an F test is not reported, the authors report and assess whether the R‐squared (goodness of fit) of the participation equation is sufficient for appropriate identification); b) the identifying instruments are individually significant (p ≤ 0.01); for Heckman models, the identifiers are reported and significant (p ≤ 0.05); c) where at least two instruments are used, the authors report on an over‐identifying test (p ≤ 0.05 is required to reject the null hypothesis); and none of the covariate controls can be affected by participation and the study convincingly assesses qualitatively why the instrument only affects the outcome via participation. If the instrument is the random assignment of the treatment, the reviewer should also assess the quality and success of the randomisation procedure in part a). d) and, for cluster‐assignment, authors particularly control for external cluster‐level factors that might confound the impact of the programme (for example, weather, infrastructure, community fixed effects, and so forth) through multivariate analysis.	Score "Yes, if a, b, c, d (if relevant) are addressed. Score "Probably yes" if one of the test required for criterion a) or b) is not reported but the other is, and the rest of the criterion are addressed and the instrument is convincing. Score “UNCLEAR” if relevant confounders are controlled for but appropriate statistical tests are not reported; or if insufficient details are provided on cluster controls Score "Probably no" if exogeneity of the instrument is not convincing and appropriate tests are not reported. Score “No” otherwise if any of the tests required for criterion a), b) or c) are reported and not satisfied.
3: Performance bias—Assessment	3. Performance bias: was the process of being observed free from motivation bias?	1= Yes, 2 = Probably Yes, 3 = Probably No, 4 = No, 8 = Unclear	a) For data collected in the context of a particular intervention trial (randomised or non‐randomised assignment), the authors state explicitly that the process of monitoring the intervention and outcome measurement is blinded, or argue convincingly why it is not likely that being monitored could affect the performance of participants in treatment and comparison groups in different ways (such as resulting in Hawthorne or John Henry effects). b) The study is based on data collected in the context of a survey, and not associated with a particular intervention trial, or data are collected from administrative records or in the context of a retrospective (ex post) evaluation.	Score “Yes” if either criterion a) or b) are satisfied; Score "Probably yes" if the study is based on survey data collected during a trial and there is no obvious issue with the monitoring processes but authors do not mention potential risks. Score “Unclear” if it is not clear whether the authors use an appropriate method to prevent Hawthorne and John Henry Effects (e.g., blinding of outcomes and, or enumerators, other methods to ensure consistent monitoring across groups). Hawthorne effects may result where participants know that they are being observed and John Henry Effects may result from participant knowledge of being compared. Score "Probably no" if there was imbalance in the frequency of monitoring in intervention groups, which might have influenced participants' behaviours. Score "No"
3: Performance bias—Justification	Performance bias—Justification	Open answer	Justification for coding decision (Include a brief summary of justification for rating, mentioning your response to all sub questions, cite relevant pages).	
4: Spill‐overs, cross‐overs and contamination—Assessment	4. Spill‐overs, cross‐overs and contamination: was the study adequately protected against spill‐overs, cross‐overs and contamination?	1= Yes, 2 = Probably Yes, 3 = Probably No, 4 = No, 8 = Unclear	a) There was no implementation issues that might have led the control participants to receive the treatment (implementer's mistake). b) The intervention is unlikely to spill‐over to comparisons (e.g., participants and non‐participants are geographically and/or socially separated from one another and general equilibrium effects are not likely) or the potential effects of spill overs were measured (e.g., variation in the % of unit within a cluster receiving the treatment). c) There is no risk of contamination by external programs: the treatment and comparisons are isolated from other interventions which might explain changes in outcomes. d) There is nothing in the surveys that might have given the control participants an idea of what the other group might receive OR they did but there is no risk that this has changed their behaviours; AND the survey process did not reveal information to the control group that they did not have before (e.g., the study aims to measure increase in take up of a service or product that participants might not know about) Authors might put something in place in the design of the study that allows to control for that survey effect (e.g., a pure control with no monitoring except baseline end line)	Score “Yes” if criterion a), b), c) and d) are satisfied; Score "Probably yes" if there is no obvious problem but there is no information reported on potential risks related to spill overs, contamination, or survey effects in the control group OR if there were issues with spill‐overs but they were controlled for or measured. Score “Unclear” if spill‐overs, cross‐overs, survey effects and/or contamination are not addressed clearly. Score "Probably no" if any of the criterion a), b), c) or d) are not satisfied but the scale of the issue is not clear. Score “No” if any of the criterion a), b), c) or d) are not satisfied and happened at a large scale in the study.
4: Spill‐overs, cross‐overs and contamination—Justification	Spill‐overs, cross‐overs and contamination—Justification	Open answer	Justification for coding decision (Include a brief summary of justification for rating, mentioning your response to all sub questions, cite relevant pages).	
5: Outcome measurement bias—Assessment	5. Outcome measurement bias	1= Yes, 2 = Probably Yes, 3 = Probably No, 4 = No, 8 = Unclear	a) Outcome assessors are blinded or the outcome measures are not likely to be biased by their judgement. b) For self‐reported outcomes: respondents in the intervention group are not more likely to have accurate answers due to recall bias; c) For self‐reported outcomes: respondents do not have incentives to over/under report something related to their performance or actions, OR researchers put in place mechanisms to reduce the risk of reporting bias (researchers not strongly involved in the implementation of the program and it is clear that their answers to the survey will not affect what they receive in the future) OR authors have measured the risks of bias through falsification tests or measuring the effect on placebo outcomes in cases where there was a risk of reporting bias. d) Timing issue: the data collection period did not differ between intervention and comparison group, the baseline data is not likely to be affected by the beginning of the intervention or affects a small percentage of the study participants.	Score “Yes” if criterion a), b), c) and d) are satisfied: Score "Probably yes" if there is a small risk related to any of a), b), c) or d) and there is no more information provided to justify the absence of bias OR if there was a high risk of bias but authors have either controlled it in their design or measured it with a placebo outcomes. Score “Unclear” if it there is a high risk related to any of a), b), c) or d) and there is no more information provided to justify the absence of bias. Score "Probably no" if there are high risk related to a), b), c) or d) and it is clear that authors were not able to control for this bias. Score “No” if there is evidence of bias.
5: Outcome measurement bias—Justification	Outcome measurement bias—Justification	Open answer	Justification for coding decision (Include a brief summary of justification for rating, mentioning your response to all sub questions, cite relevant pages).	
6: Reporting bias—Assessment	6. Selective analysis reporting: was the study free from selective analysis reporting?	1= Yes, 2 = Probably Yes, 3 = Probably No, 4 = No, 8 = Unclear	a) a pre‐analysis plan is published, especially for prospective NRS but it should also be for retrospective studies b) authors use “common” methods of estimation (i.e. credible analysis method to deal with attribution given the data available); c) There is no evidence that outcomes were selectively reported (e.g., results for all relevant outcomes in the methods section are reported in the results section); d) Requirements for specific methods of analysis: ‐ For PSM and covariate matching: (a) Where over 10% of participants fail to be matched, sensitivity analysis is used to re‐estimate results using different matching methods (Kernel Matching techniques); (b) For matching with replacement, no single observation in the control group is matched with a large number of observations in the treatment group. ‐ For IV (including Heckman) models, (a) The authors test and report the results of a Hausman test for exogeneity (p ≤ 0.05 is required to reject the null hypothesis of exogeneity); (b) the coefficient of the selectivity correction term (Rho) is significantly different from zero (P < 0.05) (Heckman approach). ‐ For studies using multivariate regression analysis, authors conduct appropriate specification tests (e.g., testing robustness of results to the inclusion of additional variables, or (very rare) reporting results of multicollinearity test, etc.)	Score “Yes” if a), b), c) and d) are satisfied OR if a) is not met and it is a retrospective NRS. Score "Probably Yes" if authors combined methods and reported relevant tests (d) only for one method OR if all the criteria are met except for a) and it is a prospective NRS Score "Unclear" if intended outcomes not specified in the paper OR if any of the requirements for d) are not reported. Score "Probably No" if b) is addressed, but authors did not present results for all outcomes announced in the method section OR did not meet requirement d) although reported. Score “No” if authors use uncommon or less rigorous estimation methods such as failure to conduct multivariate analysis for outcomes equations OR if some important outcomes are subsequently omitted from the results or the significance and magnitude of important outcomes was not assessed.
6: Reporting bias—Justification	Analysis reporting bias—Justification	Open answer	Justification for coding decision (Include a brief summary of justification for rating, mentioning your response to all sub questions, cite relevant pages).	
7: Other bias—Assessment	7. Other risks of bias: Is the study free from other sources of bias?	1= Yes, 4 = No	Score “Yes” if the reported results do not suggest any other sources of bias. Score “No” if other potential threats to validity are present, and note these here (e.g., coherence of results, survey instruments used are not reported)	
7: Other bias—Justification	Other risks of bias—Justification	Open answer	Justification for coding decision (Include a brief summary of justification for rating, mentioning your response to all sub questions, cite relevant pages).	
8: External validity	8. External validity	Open answer	Open answer‐ what do authors say about external validity, if anything?	

## APPENDIX D TITLE AND ABSTRACT SCREENING PROCEDURE

**Table:**

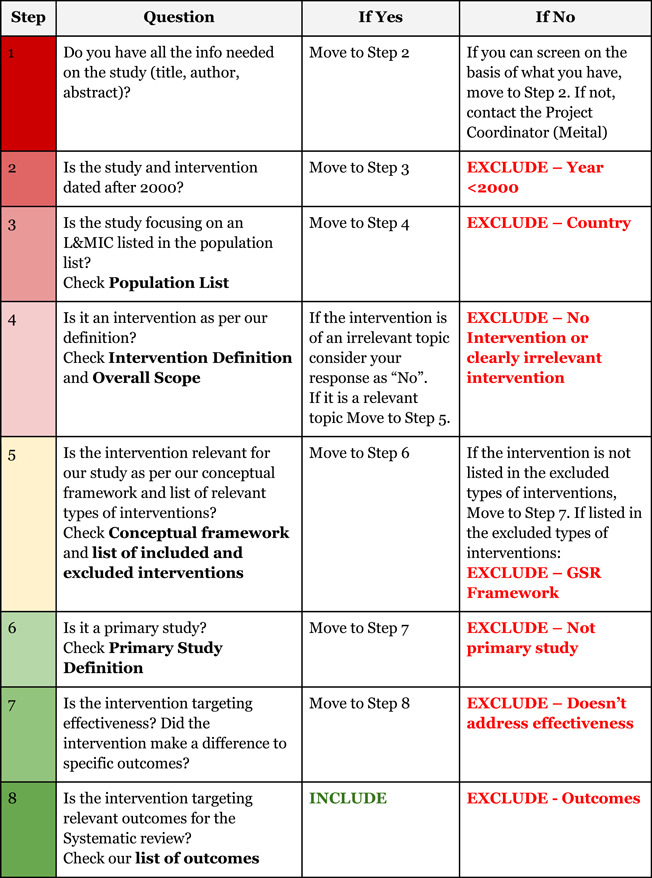

## APPENDIX E LIST OF TERMS FOR THE SEARCH STRATEGY

*Prior to developing the search strategy and as a starting point to its development, the SR team has gathered a list of key words for each of our intervention types, scope and study types. These keywords informed our search strategy, scope of work and definition of outcomes. The search strategy is available in Appendix F*.

***Intervention terms***

**Table:**

Pillar	Intervention group	Intervention terms
Participation	General definition of the Pillar	Gender opportunities
		Acceptance
		Capacities
		Capabilities
		Involvement of women and girls
		Decision‐making processes
		Political institutions
		Economic Institutions
		Social Institutions
	Civil society, associations and networks	CSOs
		Civil Society Organisation
		Women's economic associates
		Women's cooperatives
	Land rights	Women's access to land right
	Formal education	School
		Pedagogy
		Literacy
		Numeracy
		Curriculum
		Education system
		Teacher
		Gender sensitivity in formal education
		Girls and women human capital
		School vouchers
	Nonformal education	Education resources for women and girls
		Traditional schools
		Non traditional schools
		Community‐based education
		Camp‐based education
	TVET	Technical and Vocational Education and Training)
		Knowledge for employment
		Skills for employment
	MSMEs	Micro, small and medium enterprises
		Incubation
		loans
		Technical support
		Entrepreneurship training
		Mentorship
		Start‐up
	Cash‐based approaches to support women's access to and participation in education and/or the economy	Human capital
		Conditional Cash Transfers
		CCT
		Cash‐based intervention
		Participation in economy
	Financial inclusion	Access to credit
		Microcredit
		Savings
		Insurance
		Financial products
		Self‐help groups
		Village savings
		Loans
		Loans associations
		Savings groups
	Community and leisure activities	Leisure activities
		Sport
		Art
		Theatre
	Civic education and leadership	Capacity building
		Politics and political process
		Mentorship
		Community development
	Voice and participation in local and subnational governance and development bodies	Local government
		Local governance
		Quotas
		Outreach campaign
		Engagement campaign
		Subnational governance
		Enabling environment
		Accountability
		Women's rights
		Community rights
Protection pillar	General definition of the pillar	Awareness of women's and girls' rights
		Legal protection of women's and girls' rights
		Social protections for women's and girls' rights
		Risk of sexual and gender‐based violence
		SGBV
	Legal rights education	Capacity building for rights protection
		Dissemination for women's rights
		Understanding of women's rights
		Advocacy
	Behaviour change communication around support for women's rights and preventing SGBV	Behaviour change
		Attitudes change
		Beliefs change
		Role of women in society
		Social norms
		Gender norms
		Structure of social institutions
		Family training
		Media interventions
	Preventative protection measures	Incidence of violence
		SGBV risk mitigation
		Crime prevention
		Protection of vulnerable groups
	Capacity building and technical support to subnational government officials to strengthen service provision for women and gender equality	Capacity building for subnational government
		Capacity building for local government
		Gender equality official
	Gender‐sensitive policing	Capacity building of policy forces
		Police support to women and girls
		Police treatment of women and girls
		Victim protection
		Best practices
		Women‐specific police officers
		Crime reporting
	Informal judicial system	Informal justice process
		Alternative dispute resolution
Prevention	General definition of the pillar	Capacity building for prevention of SGBV
		System building for prevention of SGBV
		Accountability for violence perpetrators
		Violence perpetrator
	Conflict early warning systems	Gender responsiveness
		Conflict early warning systems
	Dialogue groups	Women‐specific dialogue groups
		Community dialogue groups
		Forums
		Moderated sessions
	Community consultations	Community consultations
		Subnational consultations
		Formal peace process
	Capacity building for conflict transformation	Capacity building for conflict transformation process
		Mediation
		Negotiation
		Conflict resolution
		Conflict prevention
	Peace education	Peace teaching
		Education to peace
		Education to role of women in peace
Relief and recovery	General definition of the pillar	Responsiveness
		Inclusivity
		Relief and recovery process
	(Re)integration of forcibly displaced populations	Forcibly displaced population
		Reintegration into host or home communities
		Economic support
		Cash‐based approach
		Cash transfer
		Voucher
		Social reintegration
		Community development inclusion
		Displaced women
	Disaster risk reduction	Gender responsiveness
		DRR
		Hazard vulnerability
		Risk assessment
		Disaster management plan
	Disarmament, demobilization and reintegration (DDR)	DDR policies
		DDR process
		Female former combattant

**Gender equality and empowerment terms**

**Table:**

Gender focus group	Search terms
Awareness, promotion, communication	Advocacy for gender equality
	Gender awareness
	gender equality promotion
	Religious militancy
Gender approach and definition	gender equality and women's empowerment (GEWE)
	Gender identity
	Gender specific
	Gender transformative
	Gendered approach
	Girl's empowerment
	women's empowerment
Gender‐based violence	Female genital mutilation and cutting (FGM/C)
	Femicide
	Gender‐based violence
	Intimate partner violence (IPV)
	Rape and sexual offences bills
	Reparations for survivors of SGBV
	Sexual and gender‐based violence
	violence against women and girls
Gender law and rights	Family Law
	Knowledge of international human and women's rights frameworks
	LGBTQIA+ rights
	Public Order Laws
	Sexual and reproductive health and rights
Gender norms, behaviours and social definitions	Gender behaviours
	gender equality
	Gender equity
	Gender norms
	Gender roles
	Inclusion/Inclusive
	Intersectionality
	Power relations
	Social structure
	Women social capital
Gender policies and international standards	CEDAW
	gender equality national policy
	Maputo Protocol
	National action plan
	National gender budgets
	UNSCR 1325
	Women, peace and security (agenda)
Gender social groups	Female combatants
	Girls and Boys
	Women diasporas
	IDPs/refugees
	Sexual and gender minorities
	Sex
	Women and Men
Issues, challenges and inequalities	Child marriage
	Discrimination on sex
	Domestic care burdens
	Early marriage
	Forced Marriage
	Gender inequalities
	Marriage and divorce bills
	Out‐of‐household income generation (Women)
	Sex work
Women and girls achievements	Economic empowerment
	Equal pay
	Fulfilment of women's potential
	Gendered employment
	Girls Achievement
	Improved women status
	Nondiscrimination
	Social Empowerment
	Women Achievements
	Women autonomy
	Women's ability
	Women's access to formal financial institutions
	Women's and human rights
	Women's capabilities
	Women's decisions
	women's leadership
	Women's role in conflict
	Women's self determination
Women and girls agency	Coalition building
	Community sensitisation
	Feminism
	Girls Agency
	Local religious and cultural leaders
	Neighbourhood committees
	Personal agency
	Women Agency
	Women peace activists
	Women representation
	Women's civil society organisations
	Women's movement building
	Women's representation in local/subnational legislatures
	Women's rights organisations
	Women's savings groups and cooperative organisations (SACO)
	Young women activists
Women and girls resources	Emergency grants for women human rights defenders (WHRDs)
	Girls Resources
	Inheritance and land access for women
	Maternity leave
	Mental health and psychosocial support
	Women access
	Women Resources

**Impact evaluation terms**

**Table:**

Impact evaluation focus group	Search terms
Experimental design	Random control trial
	Random trial
	RCT
	Evaluation
	Impact
	Assessment
Quasi experimental design	Difference in difference
	Regression
	Multilevel
	Fixed‐Effects
	Discontinuity
	Propensity score matching
	Double difference
	RDD
	Covariate matching
	Interrupted time series
	Cohort analysis
	Instrumental variable
	Evaluation Studies
	Cost analysis
	Cost effective analysis
	Systematic review
	Literature review
	Meta analysis
	Project evaluation
	Quantitative methods

## APPENDIX F SEARCH STRATEGIES

LGBT+ Searches

Latest Search in Ebsco—16th Feb 2021

Tuesday, February 16, 2021 6:40:04 AM

S30 S25 AND S29

Database—APA PsycInfo;Communication & Mass Media Complete;ERIC;Gender Studies Database;International Political Science Abstracts 0

S29 S28 NOT S26

774

S28 S1 AND S2 AND S3 AND S4 AND S27

1,542

S27 TI ((transgender* OR "gender nonconforming" OR "gender identity disorder" OR "gender dysphoria" OR "gender minorit*" OR lesbian* OR gay* OR bisexual* OR "sexual minorit*" OR "same‐sex" OR homosexual* OR "gender identity" OR non‐heterosexual* OR "non heterosexual*" OR queer* OR "non‐binary" OR "non binary" OR “LGBT*” OR "sexual dissident*" OR "gender variant" OR gender‐variant OR genderqueer* OR intersex OR "minority groups" OR TGNC OR transgender* OR "gender nonconforming" OR "men who have sex with men" OR msm OR transsexual* OR "women loving women" OR "women who have sex with women" OR WSW)) OR AB ((transgender* OR "gender nonconforming" OR "gender identity disorder" OR "gender dysphoria" OR "gender minorit*" OR lesbian* OR gay* OR bisexual* OR "sexual minorit*" OR "same‐sex" OR homosexual* OR "gender identity" OR non‐heterosexual* OR "non heterosexual*" OR queer* OR "non‐binary" OR "non binary" OR “LGBT*” OR "sexual dissident*" OR "gender variant" OR gender‐variant OR genderqueer* OR intersex OR "minority groups" OR TGNC OR transgender* OR "gender nonconforming" OR "men who have sex with men" OR msm OR transsexual* OR "women loving women" OR "women who have sex with women" OR WSW)) OR SU ((transgender* OR "gender nonconforming" OR "gender identity disorder" OR "gender dysphoria" OR "gender minorit*" OR lesbian* OR gay* OR bisexual* OR "sexual minorit*" OR "same‐sex" OR homosexual* OR "gender identity" OR non‐heterosexual* OR "non heterosexual*" OR queer* OR "non‐binary" OR "non binary" OR “LGBT*” OR "sexual dissident*" OR "gender variant" OR gender‐variant OR genderqueer* OR intersex OR "minority groups" OR TGNC OR transgender* OR "gender nonconforming" OR "men who have sex with men" OR msm OR transsexual* OR "women loving women" OR "women who have sex with women" OR WSW)) Limiters—Date Published: 20000101‐20211231; Publication Year: 2000‐2021

148,919

S26 S1 AND S2 AND S3 AND S4 AND S25

19,851

S25 S5 OR S6 OR S7 OR S8 OR S9 OR S10 OR S11 OR S12 OR S13 OR S14 OR S15 OR S16 OR S17 OR S18 OR S19 OR S20 OR S21 OR S22 OR S23 OR S24

2,055,055

S24 TI (((disarm* or demobili* or DDR) N3 (policy or policies or process* or ((female* or women) N2 (combatant* or soldier*))))) OR AB (((disarm* or demobili* or DDR) N3 (policy or policies or process* or ((female* or women) N2 (combatant* or soldier*))))) OR SU (((disarm* or demobili* or DDR) N3 (policy or policies or process* or ((female* or women) N2 (combatant* or soldier*))))) Limiters—Date Published: 20000101‐20211231

104

S23 TI (((disaster* or hazard*) N3 (vulnerab* or risk* or manag* or mitigat* or response* or responsiveness or respond*))) OR AB (((disaster* or hazard*) N3 (vulnerab* or risk* or manag* or mitigat* or response* or responsiveness or respond*))) OR SU (((disaster* or hazard*) N3 (vulnerab* or risk* or manag* or mitigat* or response* or responsiveness or respond*))) Limiters—Date Published: 20000101‐20211231

5,485

S22 TI (((displaced N2 (population* or people or communit* or women)) N3 (reintegrat* or re‐integrat* or "economic support" or cash‐based or "cash transfer*" or voucher* or inclus*))) OR AB (((displaced N2 (population* or people or communit* or women)) N3 (reintegrat* or re‐integrat* or "economic support" or cash‐based or "cash transfer*" or voucher* or inclus*))) OR SU (((displaced N2 (population* or people or communit* or women)) N3 (reintegrat* or re‐integrat* or "economic support" or cash‐based or "cash transfer*" or voucher* or inclus*))) Limiters—Date Published: 20000101‐20211231

4

S21 TI (peace N4 educat*) OR AB (peace N4 educat*) OR SU (peace N4 educat*) Limiters—Date Published: 20000101‐20211231

1,065

S20 TI (((mediat* or negotiat* or resolv* or resolution or prevent* or transform* or (capacity N2 build*)) N3 conflict*)) OR AB (((mediat* or negotiat* or resolv* or resolution or prevent* or transform* or (capacity N2 build*)) N3 conflict*)) OR SU (((mediat* or negotiat* or resolv* or resolution or prevent* or transform* or (capacity N2 build*)) N3 conflict*)) Limiters—Date Published: 20000101‐20211231

18,811

S19 TI ((formal N3 ("peace process" or peacebuilding))) OR AB ((formal N3 ("peace process" or peacebuilding))) OR SU ((formal N3 ("peace process" or peacebuilding))) Limiters—Date Published: 20000101‐20211231

5

S18 TI ((((dialog* or discuss* or consultative or consultation*) N2 (group* or forum* or communit* or subnational or local or regional)))) OR AB ((((dialog* or discuss* or consultative or consultation*) N2 (group* or forum* or communit* or subnational or local or regional)))) OR SU ((((dialog* or discuss* or consultative or consultation*) N2 (group* or forum* or communit* or subnational or local or regional)))) Limiters—Date Published: 20000101‐20211231

34,219

S17 TI (((conflict or conflicts) N3 ("early warning"))) OR AB (((conflict or conflicts) N3 ("early warning"))) OR SU (((conflict or conflicts) N3 ("early warning"))) Limiters—Date Published: 20000101‐20211231

24

S16 TI (((("gender‐based violence" or "sexual violence" or SGBV or crime) N3 ((accountab* N3 (perpetrat* or offender*)) or ((system* or capacity) N2 build*))))) OR AB (((("gender‐based violence" or "sexual violence" or SGBV or crime) N3 ((accountab* N3 (perpetrat* or offender*)) or ((system* or capacity) N2 build*))))) OR SU (((("gender‐based violence" or "sexual violence" or SGBV or crime) N3 ((accountab* N3 (perpetrat* or offender*)) or ((system* or capacity) N2 build*))))) Limiters—Date Published: 20000101‐20211231

18

S15 TI (((((justice or judicial or legal) N3 (process* or procedure*)) N2 informal) or (dispute* N2 (resolv* or resolution)))) OR AB (((((justice or judicial or legal) N3 (process* or procedure*)) N2 informal) or (dispute* N2 (resolv* or resolution)))) OR SU (((((justice or judicial or legal) N3 (process* or procedure*)) N2 informal) or (dispute* N2 (resolv* or resolution)))) Limiters—Date Published: 20000101‐20211231

1,869

S14 TI (((((capacity N2 build*) or support* or "gender sensitive" or treatment or "best practice*") N3 (police or policing)) or (crime* N3 report*) or (victim* N2 protect*))) OR AB (((((capacity N2 build*) or support* or "gender sensitive" or treatment or "best practice*") N3 (police or policing)) or (crime* N3 report*) or (victim* N2 protect*))) OR SU (((((capacity N2 build*) or support* or "gender sensitive" or treatment or "best practice*") N3 (police or policing)) or (crime* N3 report*) or (victim* N2 protect*))) Limiters—Date Published: 20000101‐20211231

3,454

S13 TI ((((capacity N2 build*) N3 ((subnational or sub‐national or local or regional) N2 (government or authorit*)) or "gender equality offic"))) OR AB ((((capacity N2 build*) N3 ((subnational or sub‐national or local or regional) N2 (government or authorit*)) or "gender equality offic"))) OR SU ((((capacity N2 build*) N3 ((subnational or sub‐national or local or regional) N2 (government or authorit*)) or "gender equality offic"))) Limiters—Date Published: 20000101‐20211231

13

S12 TI ((((incidence or prevalen*) N2 violence) or (("gender‐based violence" or "sexual violence" or SGBV or crime) N2 (mitigat* or prevent*)) or (vulnerable N3 group*))) OR AB ((((incidence or prevalen*) N2 violence) or (("gender‐based violence" or "sexual violence" or SGBV or crime) N2 (mitigat* or prevent*)) or (vulnerable N3 group*))) OR SU ((((incidence or prevalen*) N2 violence) or (("gender‐based violence" or "sexual violence" or SGBV or crime) N2 (mitigat* or prevent*)) or (vulnerable N3 group*))) Limiters—Date Published: 20000101‐20211231

10,809

S11 TI ((((behavio* or attitud* or belief*) N2 chang*) or (role N3 (societ* or communit*)) or "social norm*" or "gender norm*" or ("social institution*" N3 structur*) or ((family or families) N3 train*) or (media N3 intervention*))) OR AB ((((behavio* or attitud* or belief*) N2 chang*) or (role N3 (societ* or communit*)) or "social norm*" or "gender norm*" or ("social institution*" N3 structur*) or ((family or families) N3 train*) or (media N3 intervention*))) OR SU ((((behavio* or attitud* or belief*) N2 chang*) or (role N3 (societ* or communit*)) or "social norm*" or "gender norm*" or ("social institution*" N3 structur*) or ((family or families) N3 train*) or (media N3 intervention*))) Limiters—Date Published: 20000101‐20211231

94,738

S10 TI (((("social protection*" or (capacity N2 build*) or disseminat* or understanding or aware* or advocacy or "gender‐based violence" or "sexual violence" or SGBV)) N3 rights)) OR AB (((("social protection*" or (capacity N2 build*) or disseminat* or understanding or aware* or advocacy or "gender‐based violence" or "sexual violence" or SGBV)) N3 rights)) OR SU (((("social protection*" or (capacity N2 build*) or disseminat* or understanding or aware* or advocacy or "gender‐based violence" or "sexual violence" or SGBV)) N3 rights)) Limiters—Date Published: 20000101‐20211231

2,261

S9 TI (((capacity N3 build*) or politics or political or civic or (communit* N3 develop*) or ((local or regional) N3 (government* or governance or quota or quotas or accountab*)) or ((outreach or engag*) N3 campaign*) or (enabl* N3 (environment* or condition*)) or ((women* or girl* or communit*) N2 right*))) OR AB (((capacity N3 build*) or politics or political or civic or (communit* N3 develop*) or ((local or regional) N3 (government* or governance or quota or quotas or accountab*)) or ((outreach or engag*) N3 campaign*) or (enabl* N3 (environment* or condition*)) or ((women* or girl* or communit*) N2 right*))) OR SU (((capacity N3 build*) or politics or political or civic or (communit* N3 develop*) or ((local or regional) N3 (government* or governance or quota or quotas or accountab*)) or ((outreach or engag*) N3 campaign*) or (enabl* N3 (environment* or condition*)) or ((women* or girl* or communit*) N2 right*))) Limiters—Date Published: 20000101‐20211231

334,157

S8 TI ((leisure or sport* or art or theatr* or drama)) OR AB ((leisure or sport* or art or theatr* or drama)) OR SU ((leisure or sport* or art or theatr* or drama)) Limiters—Date Published: 20000101‐20211231

180,114

S7 TI ((MSME or MSMEs or (micro N2 small N3 enterprise*) or loan or loans or "technical support" or ((entrepreneur* or enterprise*) N3 train*) or mentor* or start‐up* or CCT or CCTs or "conditional cash transfer*" or ((cash or financ*) N2 based N3 intervention*) or ((participat* or involv*) N3 economy) or (access* N3 credit) or microcredit or micro‐credit or "micro credit" or savings or insurance or "financial product*" or (self‐help N2 (group* or club* or organisation* or organization* or association*)))) OR AB ((MSME or MSMEs or (micro N2 small N3 enterprise*) or loan or loans or "technical support" or ((entrepreneur* or enterprise*) N3 train*) or mentor* or start‐up* or CCT or CCTs or "conditional cash transfer*" or ((cash or financ*) N2 based N3 intervention*) or ((participat* or involv*) N3 economy) or (access* N3 credit) or microcredit or micro‐credit or "micro credit" or savings or insurance or "financial product*" or (self‐help N2 (group* or club* or organisation* or organization* or association*)))) OR SU ((MSME or MSMEs or (micro N2 small N3 enterprise*) or loan or loans or "technical support" or ((entrepreneur* or enterprise*) N3 train*) or mentor* or start‐up* or CCT or CCTs or "conditional cash transfer*" or ((cash or financ*) N2 based N3 intervention*) or ((participat* or involv*) N3 economy) or (access* N3 credit) or microcredit or micro‐credit or "micro credit" or savings or insurance or "financial product*" or (self‐help N2 (group* or club* or organisation* or organization* or association*)))) Limiters—Date Published: 20000101‐20211231

87,276

S6 TI ((school* or pedagog* or literacy or literate or illiterate or numeracy or numerate or curricul* or education* or teach* or voucher* or vocational or training or ((skill* or knowledge) N3 (work or employ* or job or jobs)))) OR AB ((school* or pedagog* or literacy or literate or illiterate or numeracy or numerate or curricul* or education* or teach* or voucher* or vocational or training or ((skill* or knowledge) N3 (work or employ* or job or jobs)))) OR SU ((school* or pedagog* or literacy or literate or illiterate or numeracy or numerate or curricul* or education* or teach* or voucher* or vocational or training or ((skill* or knowledge) N3 (work or employ* or job or jobs)))) Limiters—Date Published: 20000101‐20211231

1,577,244

S5 TI (("civil societ*" or CSO or CSOs or ((economic* or financ*) N2 (partner* or associate*)) or cooperative* or co‐operative* or "land right*")) OR AB (("civil societ*" or CSO or CSOs or ((economic* or financ*) N2 (partner* or associate*)) or cooperative* or co‐operative* or "land right*")) OR SU (("civil societ*" or CSO or CSOs or ((economic* or financ*) N2 (partner* or associate*)) or cooperative* or co‐operative* or "land right*")) Limiters—Date Published: 20000101‐20211231

57,644

S4 TI ((empower* or disempower* or autonomy or (decision* N2 (make or made or making)) or self‐determin* or bargain* or negotiat* or equal* or agency or transformati* or particip* or engag* or inclus* or represen* or access* or equit*)) OR AB ((empower* or disempower* or autonomy or (decision* N2 (make or made or making)) or self‐determin* or bargain* or negotiat* or equal* or agency or transformati* or particip* or engag* or inclus* or represen* or access* or equit*)) OR SU ((empower* or disempower* or autonomy or (decision* N2 (make or made or making)) or self‐determin* or bargain* or negotiat* or equal* or agency or transformati* or particip* or engag* or inclus* or represen* or access* or equit*)) Limiters—Date Published: 20000101‐20211231

1,973,969

S3 TI ((gender* or woman* or women* or mother* or maternal or female* or wife* or wives or girl* or schoolgirl* or daughter* or sex*)) OR AB ((gender* or woman* or women* or mother* or maternal or female* or wife* or wives or girl* or schoolgirl* or daughter* or sex*)) OR SU ((gender* or woman* or women* or mother* or maternal or female* or wife* or wives or girl* or schoolgirl* or daughter* or sex*)) Limiters—Date Published: 20000101‐20211231

1,510,315

S2 TI (("quasi experiment*" or quasi‐experiment* or "random* control* trial*" or "random* trial*" or rct* or (random* N3 allocat*) or evaluat* or impact* or assess* or dif‐dif or psm or "double difference" or difference‐in‐difference or rdd or "difference in difference" or "statistical matching*" or "propensity score matching" or "covariate matching" or "coarsened‐exact matching" or "propensity‐weighted" or "multiple regression" or "statistical regression" or "regression discontinuity*" or "cohort analysis" or "quantitative method*" or "program* evaluation" or "interrupted time series" or (before N5 after) or (pre N5 post) or ((pretest or "pre test") and (posttest or "post test")) or (("fixed effect*" or "random effect*") N3 (model or estimation)) or "instrumental variable" or "synthetic control" or ((quantitative or "comparison group*" or counterfactual or "counter factual" or counter‐factual or experiment*) N3 (design or study or analysis)) or ((cost* or economic) N2 (benefit or effective* or analy*)))) OR AB (("quasi experiment*" or quasi‐experiment* or "random* control* trial*" or "random* trial*" or rct* or (random* N3 allocat*) or evaluat* or impact* or assess* or dif‐dif or psm or "double difference" or difference‐in‐difference or rdd or "difference in difference" or "statistical matching*" or "propensity score matching" or "covariate matching" or "coarsened‐exact matching" or "propensity‐weighted" or "multiple regression" or "statistical regression" or "regression discontinuity*" or "cohort analysis" or "quantitative method*" or "program* evaluation" or "interrupted time series" or (before N5 after) or (pre N5 post) or ((pretest or "pre test") and (posttest or "post test")) or (("fixed effect*" or "random effect*") N3 (model or estimation)) or "instrumental variable" or "synthetic control" or ((quantitative or "comparison group*" or counterfactual or "counter factual" or counter‐factual or experiment*) N3 (design or study or analysis)) or ((cost* or economic) N2 (benefit or effective* or analy*)))) OR SU (("quasi experiment*" or quasi‐experiment* or "random* control* trial*" or "random* trial*" or rct* or (random* N3 allocat*) or evaluat* or impact* or assess* or dif‐dif or psm or "double difference" or difference‐in‐difference or rdd or "difference in difference" or "statistical matching*" or "propensity score matching" or "covariate matching" or "coarsened‐exact matching" or "propensity‐weighted" or "multiple regression" or "statistical regression" or "regression discontinuity*" or "cohort analysis" or "quantitative method*" or "program* evaluation" or "interrupted time series" or (before N5 after) or (pre N5 post) or ((pretest or "pre test") and (posttest or "post test")) or (("fixed effect*" or "random effect*") N3 (model or estimation)) or "instrumental variable" or "synthetic control" or ((quantitative or "comparison group*" or counterfactual or "counter factual" or counter‐factual or experiment*) N3 (design or study or analysis)) or ((cost* or economic) N2 (benefit or effective* or analy*)))) Limiters—Date Published: 20000101‐20211231

1,811,340

S1 TI (((afghanistan or albania or algeria or "american samoa" or angola or "antigua and barbuda" or antigua or barbuda or argentina or armenia or armenian or aruba or azerbaijan or bahrain or bangladesh or barbados or republic of belarus or belarus or byelarus or belorussia or byelorussian or belize or "british honduras" or benin or dahomey or bhutan or bolivia or "bosnia and herzegovina" or bosnia or herzegovina or botswana or bechuanaland or brazil or brasil or bulgaria or "burkina faso" or "burkina fasso" or "upper volta" or burundi or urundi or "cabo verde" or "cape verde" or cambodia or kampuchea or "khmer republic" or cameroon or cameron or cameroun or "central african republic" or "ubangi shari" or chad or chile or china or colombia or comoros or comoro islands or "iles comores" or mayotte or "democratic republic of the congo" or "democratic republic congo" or congo or zaire or "costa rica" or "cote divoire" or "cote d ivoire" or "cote divoire" or "cote d ivoire" or "ivory coast" or croatia or cuba or cyprus or "czech republic" or czechoslovakia or djibouti or "french somaliland" or dominica or "dominican republic" or ecuador or egypt or "united arab republic" or "el salvador" or "equatorial guinea" or "spanish guinea" or eritrea or estonia or eswatini or swaziland or ethiopia or fiji or gabon or "gabonese republic" or gambia or "georgia (republic) " or georgian or ghana or "gold coast" or gibraltar or greece or grenada or guam or guatemala or guinea or "guinea bissau" or guyana or "british guiana" or haiti or hispaniola or honduras or hungary or india or indonesia or timor or iran or iraq or "isle of man" or jamaica or jordan or kazakhstan or kazakh or kenya or "democratic peoples republic of korea" or "republic of korea" or "north korea" or "south korea" or korea or kosovo or kyrgyzstan or kirghizia or kirgizstan or "kyrgyz republic" or kirghiz or laos or "lao pdr" or "lao people's democratic republic" or latvia or lebanon or "lebanese republic" or lesotho or basutoland or liberia or libya or "libyan arab jamahiriya" or lithuania or macau or macao or "republic of north macedonia" or macedonia or madagascar or "malagasy republic" or malawi or nyasaland or malaysia or "malay federation" or "malaya federation" or maldives or "indian ocean islands" or "indian ocean" or mali or malta or micronesia or "federated states of micronesia" or kiribati or "marshall islands" or nauru or "northern mariana islands" or palau or tuvalu or mauritania or mauritius or mexico or moldova or moldovian or mongolia or montenegro or morocco or ifni or mozambique or "portuguese east africa" or myanmar or burma or namibia or nepal or "netherlands antilles" or nicaragua or niger or nigeria or oman or muscat or pakistan or panama or "papua new guinea" or "new guinea" or paraguay or peru or philippines or philipines or phillipines or phillippines or poland or "polish people's republic" or portugal or "portuguese republic" or "puerto rico" or romania or russia or "russian federation" or ussr or "soviet union or union of soviet socialist republics" or rwanda or ruanda or samoa or "pacific islands" or polynesia or "samoan islands" or "navigator island" or "navigator islands" or "sao tome and principe" or "saudi arabia" or senegal or serbia or seychelles or "sierra leone" or slovakia or "slovak republic" or slovenia or melanesia or "solomon island" or "solomon islands" or "norfolk island" or "norfolk islands" or somalia or "south africa" or "south sudan" or "sri lanka" or ceylon or "saint kitts and nevis" or "st. kitts and nevis" or "saint lucia" or "st. lucia" or "saint vincent and the grenadines" or "saint vincent" or "st. vincent" or grenadines or sudan or suriname or surinam or "dutch guiana" or "netherlands guiana" or syria or "syrian arab republic" or tajikistan or tadjikistan or tadzhikistan or tadzhik or tanzania or tanganyika or thailand or siam or "timor leste" or "east timor" or togo or "togolese republic" or tonga or "trinidad and tobago" or trinidad or tobago or tunisia or turkey or turkmenistan or turkmen or uganda or ukraine or uruguay or uzbekistan or uzbek or vanuatu or "new hebrides" or venezuela or vietnam or "viet nam" or "middle east" or "west bank" or gaza or palestine or yemen or yugoslavia or zambia or zimbabwe or "northern rhodesia" or "global south" or "africa south of the sahara" or "sub‐saharan africa" or "subsaharan africa" or "africa, central" or "central africa" or "africa, northern" or "north africa" or "northern africa" or magreb or maghrib or sahara or "africa, southern" or "southern africa" or "africa, eastern" or "east africa" or "eastern africa" or "africa, western" or "west africa" or "western africa" or "west indies" or "indian ocean islands" or caribbean or "central america" or "latin america" or "south and central america" or "south america" or "asia, central" or "central asia" or "asia, northern" or "north asia" or "northern asia" or "asia, southeastern" or "southeastern asia" or "south eastern asia" or "southeast asia" or "south east asia" or "asia, western" or "western asia" or "europe, eastern" or "east europe" or "eastern europe" or "developing country" or "developing countries" or "developing nation?" or "developing population?" or "developing world" or "less developed countr*" or "less developed nation?" or "less developed population?" or "less developed world" or "lesser developed countr*" or "lesser developed nation?" or "lesser developed population?" or "lesser developed world" or "under developed countr*" or "under developed nation?" or "under developed population?" or "under developed world" or "underdeveloped countr*" or "underdeveloped nation?" or "underdeveloped population?" or "underdeveloped world" or "middle income countr*" or "middle income nation?" or "middle income population?" or "low income countr*" or "low income nation?" or "low income population?" or "lower income countr*" or "lower income nation?" or "lower income population?" or "underserved countr*" or "underserved nation?" or "underserved population?" or "underserved world" or "under served countr*" or "under served nation?" or "under served population?" or "under served world" or "deprived countr*" or "deprived nation?" or "deprived population?" or "deprived world" or "poor countr*" or "poor nation?" or "poor population?" or "poor world" or "poorer countr*" or "poorer nation?" or "poorer population?" or "poorer world" or "developing econom*" or "less developed econom*" or "lesser developed econom*" or "under developed econom*" or "underdeveloped econom*" or "middle income econom*" or "low income econom*" or "lower income econom*" or "low gdp" or "low gnp" or "low gross domestic" or "low gross national" or "lower gdp" or "lower gnp" or "lower gross domestic" or "lower gross national" or lmic or lmics or "third world" or "lami countr*" or "transitional countr*" or "emerging economies" or "emerging nation?"))) OR AB (((afghanistan or albania or algeria or "american samoa" or angola or "antigua and barbuda" or antigua or barbuda or argentina or armenia or armenian or aruba or azerbaijan or bahrain or bangladesh or barbados or republic of belarus or belarus or byelarus or belorussia or byelorussian or belize or "british honduras" or benin or dahomey or bhutan or bolivia or "bosnia and herzegovina" or bosnia or herzegovina or botswana or bechuanaland or brazil or brasil or bulgaria or "burkina faso" or "burkina fasso" or "upper volta" or burundi or urundi or "cabo verde" or "cape verde" or cambodia or kampuchea or "khmer republic" or cameroon or cameron or cameroun or "central african republic" or "ubangi shari" or chad or chile or china or colombia or comoros or comoro islands or "iles comores" or mayotte or "democratic republic of the congo" or "democratic republic congo" or congo or zaire or "costa rica" or "cote divoire" or "cote d ivoire" or "cote divoire" or "cote d ivoire" or "ivory coast" or croatia or cuba or cyprus or "czech republic" or czechoslovakia or djibouti or "french somaliland" or dominica or "dominican republic" or ecuador or egypt or "united arab republic" or "el salvador" or "equatorial guinea" or "spanish guinea" or eritrea or estonia or eswatini or swaziland or ethiopia or fiji or gabon or "gabonese republic" or gambia or "georgia (republic) " or georgian or ghana or "gold coast" or gibraltar or greece or grenada or guam or guatemala or guinea or "guinea bissau" or guyana or "british guiana" or haiti or hispaniola or honduras or hungary or india or indonesia or timor or iran or iraq or "isle of man" or jamaica or jordan or kazakhstan or kazakh or kenya or "democratic peoples republic of korea" or "republic of korea" or "north korea" or "south korea" or korea or kosovo or kyrgyzstan or kirghizia or kirgizstan or "kyrgyz republic" or kirghiz or laos or "lao pdr" or "lao people's democratic republic" or latvia or lebanon or "lebanese republic" or lesotho or basutoland or liberia or libya or "libyan arab jamahiriya" or lithuania or macau or macao or "republic of north macedonia" or macedonia or madagascar or "malagasy republic" or malawi or nyasaland or malaysia or "malay federation" or "malaya federation" or maldives or "indian ocean islands" or "indian ocean" or mali or malta or micronesia or "federated states of micronesia" or kiribati or "marshall islands" or nauru or "northern mariana islands" or palau or tuvalu or mauritania or mauritius or mexico or moldova or moldovian or mongolia or montenegro or morocco or ifni or mozambique or "portuguese east africa" or myanmar or burma or namibia or nepal or "netherlands antilles" or nicaragua or niger or nigeria or oman or muscat or pakistan or panama or "papua new guinea" or "new guinea" or paraguay or peru or philippines or philipines or phillipines or phillippines or poland or "polish people's republic" or portugal or "portuguese republic" or "puerto rico" or romania or russia or "russian federation" or ussr or "soviet union or union of soviet socialist republics" or rwanda or ruanda or samoa or "pacific islands" or polynesia or "samoan islands" or "navigator island" or "navigator islands" or "sao tome and principe" or "saudi arabia" or senegal or serbia or seychelles or "sierra leone" or slovakia or "slovak republic" or slovenia or melanesia or "solomon island" or "solomon islands" or "norfolk island" or "norfolk islands" or somalia or "south africa" or "south sudan" or "sri lanka" or ceylon or "saint kitts and nevis" or "st. kitts and nevis" or "saint lucia" or "st. lucia" or "saint vincent and the grenadines" or "saint vincent" or "st. vincent" or grenadines or sudan or suriname or surinam or "dutch guiana" or "netherlands guiana" or syria or "syrian arab republic" or tajikistan or tadjikistan or tadzhikistan or tadzhik or tanzania or tanganyika or thailand or siam or "timor leste" or "east timor" or togo or "togolese republic" or tonga or "trinidad and tobago" or trinidad or tobago or tunisia or turkey or turkmenistan or turkmen or uganda or ukraine or uruguay or uzbekistan or uzbek or vanuatu or "new hebrides" or venezuela or vietnam or "viet nam" or "middle east" or "west bank" or gaza or palestine or yemen or yugoslavia or zambia or zimbabwe or "northern rhodesia" or "global south" or "africa south of the sahara" or "sub‐saharan africa" or "subsaharan africa" or "africa, central" or "central africa" or "africa, northern" or "north africa" or "northern africa" or magreb or maghrib or sahara or "africa, southern" or "southern africa" or "africa, eastern" or "east africa" or "eastern africa" or "africa, western" or "west africa" or "western africa" or "west indies" or "indian ocean islands" or caribbean or "central america" or "latin america" or "south and central america" or "south america" or "asia, central" or "central asia" or "asia, northern" or "north asia" or "northern asia" or "asia, southeastern" or "southeastern asia" or "south eastern asia" or "southeast asia" or "south east asia" or "asia, western" or "western asia" or "europe, eastern" or "east europe" or "eastern europe" or "developing country" or "developing countries" or "developing nation?" or "developing population?" or "developing world" or "less developed countr*" or "less developed nation?" or "less developed population?" or "less developed world" or "lesser developed countr*" or "lesser developed nation?" or "lesser developed population?" or "lesser developed world" or "under developed countr*" or "under developed nation?" or "under developed population?" or "under developed world" or "underdeveloped countr*" or "underdeveloped nation?" or "underdeveloped population?" or "underdeveloped world" or "middle income countr*" or "middle income nation?" or "middle income population?" or "low income countr*" or "low income nation?" or "low income population?" or "lower income countr*" or "lower income nation?" or "lower income population?" or "underserved countr*" or "underserved nation?" or "underserved population?" or "underserved world" or "under served countr*" or "under served nation?" or "under served population?" or "under served world" or "deprived countr*" or "deprived nation?" or "deprived population?" or "deprived world" or "poor countr*" or "poor nation?" or "poor population?" or "poor world" or "poorer countr*" or "poorer nation?" or "poorer population?" or "poorer world" or "developing econom*" or "less developed econom*" or "lesser developed econom*" or "under developed econom*" or "underdeveloped econom*" or "middle income econom*" or "low income econom*" or "lower income econom*" or "low gdp" or "low gnp" or "low gross domestic" or "low gross national" or "lower gdp" or "lower gnp" or "lower gross domestic" or "lower gross national" or lmic or lmics or "third world" or "lami countr*" or "transitional countr*" or "emerging economies" or "emerging nation?"))) OR SU (((afghanistan or albania or algeria or "american samoa" or angola or "antigua and barbuda" or antigua or barbuda or argentina or armenia or armenian or aruba or azerbaijan or bahrain or bangladesh or barbados or republic of belarus or belarus or byelarus or belorussia or byelorussian or belize or "british honduras" or benin or dahomey or bhutan or bolivia or "bosnia and herzegovina" or bosnia or herzegovina or botswana or bechuanaland or brazil or brasil or bulgaria or "burkina faso" or "burkina fasso" or "upper volta" or burundi or urundi or "cabo verde" or "cape verde" or cambodia or kampuchea or "khmer republic" or cameroon or cameron or cameroun or "central african republic" or "ubangi shari" or chad or chile or china or colombia or comoros or comoro islands or "iles comores" or mayotte or "democratic republic of the congo" or "democratic republic congo" or congo or zaire or "costa rica" or "cote divoire" or "cote d ivoire" or "cote divoire" or "cote d ivoire" or "ivory coast" or croatia or cuba or cyprus or "czech republic" or czechoslovakia or djibouti or "french somaliland" or dominica or "dominican republic" or ecuador or egypt or "united arab republic" or "el salvador" or "equatorial guinea" or "spanish guinea" or eritrea or estonia or eswatini or swaziland or ethiopia or fiji or gabon or "gabonese republic" or gambia or "georgia (republic) " or georgian or ghana or "gold coast" or gibraltar or greece or grenada or guam or guatemala or guinea or "guinea bissau" or guyana or "british guiana" or haiti or hispaniola or honduras or hungary or india or indonesia or timor or iran or iraq or "isle of man" or jamaica or jordan or kazakhstan or kazakh or kenya or "democratic peoples republic of korea" or "republic of korea" or "north korea" or "south korea" or korea or kosovo or kyrgyzstan or kirghizia or kirgizstan or "kyrgyz republic" or kirghiz or laos or "lao pdr" or "lao people's democratic republic" or latvia or lebanon or "lebanese republic" or lesotho or basutoland or liberia or libya or "libyan arab jamahiriya" or lithuania or macau or macao or "republic of north macedonia" or macedonia or madagascar or "malagasy republic" or malawi or nyasaland or malaysia or "malay federation" or "malaya federation" or maldives or "indian ocean islands" or "indian ocean" or mali or malta or micronesia or "federated states of micronesia" or kiribati or "marshall islands" or nauru or "northern mariana islands" or palau or tuvalu or mauritania or mauritius or mexico or moldova or moldovian or mongolia or montenegro or morocco or ifni or mozambique or "portuguese east africa" or myanmar or burma or namibia or nepal or "netherlands antilles" or nicaragua or niger or nigeria or oman or muscat or pakistan or panama or "papua new guinea" or "new guinea" or paraguay or peru or philippines or philipines or phillipines or phillippines or poland or "polish people's republic" or portugal or "portuguese republic" or "puerto rico" or romania or russia or "russian federation" or ussr or "soviet union or union of soviet socialist republics" or rwanda or ruanda or samoa or "pacific islands" or polynesia or "samoan islands" or "navigator island" or "navigator islands" or "sao tome and principe" or "saudi arabia" or senegal or serbia or seychelles or "sierra leone" or slovakia or "slovak republic" or slovenia or melanesia or "solomon island" or "solomon islands" or "norfolk island" or "norfolk islands" or somalia or "south africa" or "south sudan" or "sri lanka" or ceylon or "saint kitts and nevis" or "st. kitts and nevis" or "saint lucia" or "st. lucia" or "saint vincent and the grenadines" or "saint vincent" or "st. vincent" or grenadines or sudan or suriname or surinam or "dutch guiana" or "netherlands guiana" or syria or "syrian arab republic" or tajikistan or tadjikistan or tadzhikistan or tadzhik or tanzania or tanganyika or thailand or siam or "timor leste" or "east timor" or togo or "togolese republic" or tonga or "trinidad and tobago" or trinidad or tobago or tunisia or turkey or turkmenistan or turkmen or uganda or ukraine or uruguay or uzbekistan or uzbek or vanuatu or "new hebrides" or venezuela or vietnam or "viet nam" or "middle east" or "west bank" or gaza or palestine or yemen or yugoslavia or zambia or zimbabwe or "northern rhodesia" or "global south" or "africa south of the sahara" or "sub‐saharan africa" or "subsaharan africa" or "africa, central" or "central africa" or "africa, northern" or "north africa" or "northern africa" or magreb or maghrib or sahara or "africa, southern" or "southern africa" or "africa, eastern" or "east africa" or "eastern africa" or "africa, western" or "west africa" or "western africa" or "west indies" or "indian ocean islands" or caribbean or "central america" or "latin america" or "south and central america" or "south america" or "asia, central" or "central asia" or "asia, northern" or "north asia" or "northern asia" or "asia, southeastern" or "southeastern asia" or "south eastern asia" or "southeast asia" or "south east asia" or "asia, western" or "western asia" or "europe, eastern" or "east europe" or "eastern europe" or "developing country" or "developing countries" or "developing nation?" or "developing population?" or "developing world" or "less developed countr*" or "less developed nation?" or "less developed population?" or "less developed world" or "lesser developed countr*" or "lesser developed nation?" or "lesser developed population?" or "lesser developed world" or "under developed countr*" or "under developed nation?" or "under developed population?" or "under developed world" or "underdeveloped countr*" or "underdeveloped nation?" or "underdeveloped population?" or "underdeveloped world" or "middle income countr*" or "middle income nation?" or "middle income population?" or "low income countr*" or "low income nation?" or "low income population?" or "lower income countr*" or "lower income nation?" or "lower income population?" or "underserved countr*" or "underserved nation?" or "underserved population?" or "underserved world" or "under served countr*" or "under served nation?" or "under served population?" or "under served world" or "deprived countr*" or "deprived nation?" or "deprived population?" or "deprived world" or "poor countr*" or "poor nation?" or "poor population?" or "poor world" or "poorer countr*" or "poorer nation?" or "poorer population?" or "poorer world" or "developing econom*" or "less developed econom*" or "lesser developed econom*" or "under developed econom*" or "underdeveloped econom*" or "middle income econom*" or "low income econom*" or "lower income econom*" or "low gdp" or "low gnp" or "low gross domestic" or "low gross national" or "lower gdp" or "lower gnp" or "lower gross domestic" or "lower gross national" or lmic or lmics or "third world" or "lami countr*" or "transitional countr*" or "emerging economies" or "emerging nation?"))) Limiters—Date Published: 20000101‐20211231

Search modes—Boolean/Phrase Interface—EBSCOhost Research Databases

Search Screen—Advanced Search

Database—APA PsycInfo;Communication & Mass Media Complete;ERIC;Gender Studies Database;International Political Science Abstracts 492,226

**Social Sciences Citation Index & Arts & Humanities Index (Web of Science)**—**Revised search based on Ada's changes**—**19th December 2020**

# 28 **25,868**

**#27 AND #6 AND #5 AND #4 AND #3**

# 27 1,550,538

#26 OR #25 OR #24 OR #23 OR #22 OR #21 OR #20 OR #19 OR #18 OR #17 OR #16 OR #15 OR #14 OR #13 OR #12 OR #11 OR #10 OR #9 OR #8 OR #7

# 26 96

TS=((disarm* or demobili* or DDR) NEAR/3 (policy or policies or process* or ((female* or women) NEAR/2 (combatant* or soldier*))))

# 25 12,636

TS=((disaster* or hazard*) NEAR/3 (vulnerab* or risk* or manag* or mitigat* or response* or responsiveness or respond*))

# 24 3

TS=((displaced NEAR/2 (population* or people or communit* or women)) NEAR/3 (reintegrat* or re‐integrat* or "economic support" or cash‐based or "cash transfer*" or voucher* or inclus*))

# 23 326

TS=(peace NEAR/4 educat*)

# 22 10,797

TS=((mediat* or negotiat* or resolv* or resolution or prevent* or transform* or (capacity NEAR/2 build*)) NEAR/3 conflict*)

# 21 5

TS=(formal NEAR/3 ("peace process" or peacebuilding))

# 20 22,965

TS=(((dialog* or discuss* or consultative or consultation*) NEAR/2 (group* or forum* or communit* or subnational or local or regional)))

# 19 16

TS=((conflict or conflicts) NEAR/3 ("early warning"))

# 18 15

TS=((("gender‐based violence" or "sexual violence" or SGBV or crime) NEAR/3 ((accountab* NEAR/3 (perpetrat* or offender*)) or ((system* or capacity) NEAR/2 build*))))

# 17 2,380

TS=((((justice or judicial or legal) NEAR/3 (process* or procedure*)) NEAR/2 informal) or (dispute* NEAR/2 (resolv* or resolution)))

# 16 2,525

TS=((((capacity NEAR/2 build*) or support* or "gender sensitive" or treatment or "best practice*") NEAR/3 (police or policing)) or (crime* NEAR/3 report*) or (victim* NEAR/2 protect*))

# 15 28

TS=(((capacity NEAR/2 build*) NEAR/3 ((subnational or sub‐national or local or regional) NEAR/2 (government or authorit*)) or "gender equality offic"))

# 14 8,756

TS=(((incidence or prevalen*) NEAR/2 violence) or (("gender‐based violence" or "sexual violence" or SGBV or crime) NEAR/2 (mitigat* or prevent*)) or (vulnerable NEAR/3 group*))

# 13 66,399

TS=(((behavio* or attitud* or belief*) NEAR/2 chang*) or (role NEAR/3 (societ* or communit*)) or "social norm*" or "gender norm*" or ("social institution*" NEAR/3 structur*) or ((family or families) NEAR/3 train*) or (media NEAR/3 intervention*))

# 12 2,026

TS=((("social protection*" or (capacity NEAR/2 build*) or disseminat* or understanding or aware* or advocacy or "gender‐based violence" or "sexual violence" or SGBV)) NEAR/3 rights)

# 11 411,130

TS=((capacity NEAR/3 build*) or politics or political or civic or (communit* NEAR/3 develop*) or ((local or regional) NEAR/3 (government* or governance or quota or quotas or accountab*)) or ((outreach or engag*) NEAR/3 campaign*) or (enabl* NEAR/3 (environment* or condition*)) or ((women* or girl* or communit*) NEAR/2 right*))

# 10 207,686

TS=(leisure or sport* or art or theatr* or drama)

# 9 113,585

TS=(MSME or MSMEs or (micro NEAR/2 small NEAR/3 enterprise*) or loan or loans or "technical support" or ((entrepreneur* or enterprise*) NEAR/3 train*) or mentor* or start‐up* or CCT or CCTs or "conditional cash transfer*" or ((cash or financ*) NEAR/2 based NEAR/3 intervention*) or ((participat* or involv*) NEAR/3 economy) or (access* NEAR/3 credit) or microcredit or micro‐credit or "micro credit" or savings or insurance or "financial product*" or (self‐help NEAR/2 (group* or club* or organisation* or organization* or association*)))

# 8 844,800

TS=(school* or pedagog* or literacy or literate or illiterate or numeracy or numerate or curricul* or education* or teach* or voucher* or vocational or training or ((skill* or knowledge) NEAR/3 (work or employ* or job or jobs)))

# 7 43,370

TS=("civil societ*" or CSO or CSOs or ((economic* or financ*) NEAR/2 (partner* or associate*)) or cooperative* or co‐operative* or "land right*")

# 6 1,686,361

TS=(empower* or disempower* or autonomy or (decision* NEAR/2 (make or made or making)) or self‐determin* or bargain* or negotiat* or **equal*** or agency or transformati* **or particip* or engag* or inclus* or represen* or access* or equit***)

# 5 864,225

TS=(gender* or woman* or women* or mother* or maternal or female* or wife* or wives or girl* or schoolgirl* or daughter* or **sex***)

# 4 1,627,317

TS=("quasi experiment*" or quasi‐experiment* or "random* control* trial*" or "random* trial*" or rct* or (random* NEAR/3 allocat*) or evaluat* or impact* or assess* or dif‐dif or psm or "double difference" or difference‐in‐difference or rdd or "difference in difference" or "statistical matching*" or "propensity score matching" or "covariate matching" or "coarsened‐exact matching" or "propensity‐weighted" or "multiple regression" or "statistical regression" or "regression discontinuity*" or "cohort analysis" or "quantitative method*" or "program* evaluation" or "interrupted time series" or (before NEAR/5 after) or (pre NEAR/5 post) or ((pretest or "pre test") and (posttest or "post test")) or (("fixed effect*" or "random effect*") NEAR/3 (model or estimation)) or "instrumental variable" or "synthetic control" or ((quantitative or "comparison group*" or counterfactual or "counter factual" or counter‐factual or experiment*) NEAR/3 (design or study or analysis)) or ((cost* or economic) NEAR/2 (benefit or effective* or analy*)))

# 3 1,383,181

#2 OR #1

# 2 997,727

CU=((afghanistan or albania or algeria or "american samoa" or angola or "antigua and barbuda" or antigua or barbuda or argentina or armenia or armenian or aruba or azerbaijan or bahrain or bangladesh or barbados or republic of belarus or belarus or byelarus or belorussia or byelorussian or belize or "british honduras" or benin or dahomey or bhutan or bolivia or "bosnia and herzegovina" or bosnia or herzegovina or botswana or bechuanaland or brazil or brasil or bulgaria or "burkina faso" or "burkina fasso" or "upper volta" or burundi or urundi or "cabo verde" or "cape verde" or cambodia or kampuchea or "khmer republic" or cameroon or cameron or cameroun or "central african republic" or "ubangi shari" or chad or chile or china or colombia or comoros or comoro islands or "iles comores" or mayotte or "democratic republic of the congo" or "democratic republic congo" or congo or zaire or "costa rica" or "cote divoire" or "cote d ivoire" or "cote divoire" or "cote d ivoire" or "ivory coast" or croatia or cuba or cyprus or "czech republic" or czechoslovakia or djibouti or "french somaliland" or dominica or "dominican republic" or ecuador or egypt or "united arab republic" or "el salvador" or "equatorial guinea" or "spanish guinea" or eritrea or estonia or eswatini or swaziland or ethiopia or fiji or gabon or "gabonese republic" or gambia or "georgia (republic) " or georgian or ghana or "gold coast" or gibraltar or greece or grenada or guam or guatemala or guinea or "guinea bissau" or guyana or "british guiana" or haiti or hispaniola or honduras or hungary or india or indonesia or timor or iran or iraq or "isle of man" or jamaica or jordan or kazakhstan or kazakh or kenya or "democratic peoples republic of korea" or "republic of korea" or "north korea" or "south korea" or korea or kosovo or kyrgyzstan or kirghizia or kirgizstan or "kyrgyz republic" or kirghiz or laos or "lao pdr" or "lao people's democratic republic" or latvia or lebanon or "lebanese republic" or lesotho or basutoland or liberia or libya or "libyan arab jamahiriya" or lithuania or macau or macao or "republic of north macedonia" or macedonia or madagascar or "malagasy republic" or malawi or nyasaland or malaysia or "malay federation" or "malaya federation" or maldives or "indian ocean islands" or "indian ocean" or mali or malta or micronesia or "federated states of micronesia" or kiribati or "marshall islands" or nauru or "northern mariana islands" or palau or tuvalu or mauritania or mauritius or mexico or moldova or moldovian or mongolia or montenegro or morocco or ifni or mozambique or "portuguese east africa" or myanmar or burma or namibia or nepal or "netherlands antilles" or nicaragua or niger or nigeria or oman or muscat or pakistan or panama or "papua new guinea" or "new guinea" or paraguay or peru or philippines or philipines or phillipines or phillippines or poland or "polish people's republic" or portugal or "portuguese republic" or "puerto rico" or romania or russia or "russian federation" or ussr or "soviet union or union of soviet socialist republics" or rwanda or ruanda or samoa or "pacific islands" or polynesia or "samoan islands" or "navigator island" or "navigator islands" or "sao tome and principe" or "saudi arabia" or senegal or serbia or seychelles or "sierra leone" or slovakia or "slovak republic" or slovenia or melanesia or "solomon island" or "solomon islands" or "norfolk island" or "norfolk islands" or somalia or "south africa" or "south sudan" or "sri lanka" or ceylon or "saint kitts and nevis" or "st. kitts and nevis" or "saint lucia" or "st. lucia" or "saint vincent and the grenadines" or "saint vincent" or "st. vincent" or grenadines or sudan or suriname or surinam or "dutch guiana" or "netherlands guiana" or syria or "syrian arab republic" or tajikistan or tadjikistan or tadzhikistan or tadzhik or tanzania or tanganyika or thailand or siam or "timor leste" or "east timor" or togo or "togolese republic" or tonga or "trinidad and tobago" or trinidad or tobago or tunisia or turkey or turkmenistan or turkmen or uganda or ukraine or uruguay or uzbekistan or uzbek or vanuatu or "new hebrides" or venezuela or vietnam or "viet nam" or "middle east" or "west bank" or gaza or palestine or yemen or yugoslavia or zambia or zimbabwe or "northern rhodesia" or "global south" or "africa south of the sahara" or "sub‐saharan africa" or "subsaharan africa" or "africa, central" or "central africa" or "africa, northern" or "north africa" or "northern africa" or magreb or maghrib or sahara or "africa, southern" or "southern africa" or "africa, eastern" or "east africa" or "eastern africa" or "africa, western" or "west africa" or "western africa" or "west indies" or "indian ocean islands" or caribbean or "central america" or "latin america" or "south and central america" or "south america" or "asia, central" or "central asia" or "asia, northern" or "north asia" or "northern asia" or "asia, southeastern" or "southeastern asia" or "south eastern asia" or "southeast asia" or "south east asia" or "asia, western" or "western asia" or "europe, eastern" or "east europe" or "eastern europe" or "developing country" or "developing countries" or "developing nation?" or "developing population?" or "developing world" or "less developed countr*" or "less developed nation?" or "less developed population?" or "less developed world" or "lesser developed countr*" or "lesser developed nation?" or "lesser developed population?" or "lesser developed world" or "under developed countr*" or "under developed nation?" or "under developed population?" or "under developed world" or "underdeveloped countr*" or "underdeveloped nation?" or "underdeveloped population?" or "underdeveloped world" or "middle income countr*" or "middle income nation?" or "middle income population?" or "low income countr*" or "low income nation?" or "low income population?" or "lower income countr*" or "lower income nation?" or "lower income population?" or "underserved countr*" or "underserved nation?" or "underserved population?" or "underserved world" or "under served countr*" or "under served nation?" or "under served population?" or "under served world" or "deprived countr*" or "deprived nation?" or "deprived population?" or "deprived world" or "poor countr*" or "poor nation?" or "poor population?" or "poor world" or "poorer countr*" or "poorer nation?" or "poorer population?" or "poorer world" or "developing econom*" or "less developed econom*" or "lesser developed econom*" or "under developed econom*" or "underdeveloped econom*" or "middle income econom*" or "low income econom*" or "lower income econom*" or "low gdp" or "low gnp" or "low gross domestic" or "low gross national" or "lower gdp" or "lower gnp" or "lower gross domestic" or "lower gross national" or lmic or lmics or "third world" or "lami countr*" or "transitional countr*" or "emerging economies" or "emerging nation?"))

# 1 793,118

TS=((afghanistan or albania or algeria or "american samoa" or angola or "antigua and barbuda" or antigua or barbuda or argentina or armenia or armenian or aruba or azerbaijan or bahrain or bangladesh or barbados or republic of belarus or belarus or byelarus or belorussia or byelorussian or belize or "british honduras" or benin or dahomey or bhutan or bolivia or "bosnia and herzegovina" or bosnia or herzegovina or botswana or bechuanaland or brazil or brasil or bulgaria or "burkina faso" or "burkina fasso" or "upper volta" or burundi or urundi or "cabo verde" or "cape verde" or cambodia or kampuchea or "khmer republic" or cameroon or cameron or cameroun or "central african republic" or "ubangi shari" or chad or chile or china or colombia or comoros or comoro islands or "iles comores" or mayotte or "democratic republic of the congo" or "democratic republic congo" or congo or zaire or "costa rica" or "cote divoire" or "cote d ivoire" or "cote divoire" or "cote d ivoire" or "ivory coast" or croatia or cuba or cyprus or "czech republic" or czechoslovakia or djibouti or "french somaliland" or dominica or "dominican republic" or ecuador or egypt or "united arab republic" or "el salvador" or "equatorial guinea" or "spanish guinea" or eritrea or estonia or eswatini or swaziland or ethiopia or fiji or gabon or "gabonese republic" or gambia or "georgia (republic) " or georgian or ghana or "gold coast" or gibraltar or greece or grenada or guam or guatemala or guinea or "guinea bissau" or guyana or "british guiana" or haiti or hispaniola or honduras or hungary or india or indonesia or timor or iran or iraq or "isle of man" or jamaica or jordan or kazakhstan or kazakh or kenya or "democratic peoples republic of korea" or "republic of korea" or "north korea" or "south korea" or korea or kosovo or kyrgyzstan or kirghizia or kirgizstan or "kyrgyz republic" or kirghiz or laos or "lao pdr" or "lao people's democratic republic" or latvia or lebanon or "lebanese republic" or lesotho or basutoland or liberia or libya or "libyan arab jamahiriya" or lithuania or macau or macao or "republic of north macedonia" or macedonia or madagascar or "malagasy republic" or malawi or nyasaland or malaysia or "malay federation" or "malaya federation" or maldives or "indian ocean islands" or "indian ocean" or mali or malta or micronesia or "federated states of micronesia" or kiribati or "marshall islands" or nauru or "northern mariana islands" or palau or tuvalu or mauritania or mauritius or mexico or moldova or moldovian or mongolia or montenegro or morocco or ifni or mozambique or "portuguese east africa" or myanmar or burma or namibia or nepal or "netherlands antilles" or nicaragua or niger or nigeria or oman or muscat or pakistan or panama or "papua new guinea" or "new guinea" or paraguay or peru or philippines or philipines or phillipines or phillippines or poland or "polish people's republic" or portugal or "portuguese republic" or "puerto rico" or romania or russia or "russian federation" or ussr or "soviet union or union of soviet socialist republics" or rwanda or ruanda or samoa or "pacific islands" or polynesia or "samoan islands" or "navigator island" or "navigator islands" or "sao tome and principe" or "saudi arabia" or senegal or serbia or seychelles or "sierra leone" or slovakia or "slovak republic" or slovenia or melanesia or "solomon island" or "solomon islands" or "norfolk island" or "norfolk islands" or somalia or "south africa" or "south sudan" or "sri lanka" or ceylon or "saint kitts and nevis" or "st. kitts and nevis" or "saint lucia" or "st. lucia" or "saint vincent and the grenadines" or "saint vincent" or "st. vincent" or grenadines or sudan or suriname or surinam or "dutch guiana" or "netherlands guiana" or syria or "syrian arab republic" or tajikistan or tadjikistan or tadzhikistan or tadzhik or tanzania or tanganyika or thailand or siam or "timor leste" or "east timor" or togo or "togolese republic" or tonga or "trinidad and tobago" or trinidad or tobago or tunisia or turkey or turkmenistan or turkmen or uganda or ukraine or uruguay or uzbekistan or uzbek or vanuatu or "new hebrides" or venezuela or vietnam or "viet nam" or "middle east" or "west bank" or gaza or palestine or yemen or yugoslavia or zambia or zimbabwe or "northern rhodesia" or "global south" or "africa south of the sahara" or "sub‐saharan africa" or "subsaharan africa" or "africa, central" or "central africa" or "africa, northern" or "north africa" or "northern africa" or magreb or maghrib or sahara or "africa, southern" or "southern africa" or "africa, eastern" or "east africa" or "eastern africa" or "africa, western" or "west africa" or "western africa" or "west indies" or "indian ocean islands" or caribbean or "central america" or "latin america" or "south and central america" or "south america" or "asia, central" or "central asia" or "asia, northern" or "north asia" or "northern asia" or "asia, southeastern" or "southeastern asia" or "south eastern asia" or "southeast asia" or "south east asia" or "asia, western" or "western asia" or "europe, eastern" or "east europe" or "eastern europe" or "developing country" or "developing countries" or "developing nation?" or "developing population?" or "developing world" or "less developed countr*" or "less developed nation?" or "less developed population?" or "less developed world" or "lesser developed countr*" or "lesser developed nation?" or "lesser developed population?" or "lesser developed world" or "under developed countr*" or "under developed nation?" or "under developed population?" or "under developed world" or "underdeveloped countr*" or "underdeveloped nation?" or "underdeveloped population?" or "underdeveloped world" or "middle income countr*" or "middle income nation?" or "middle income population?" or "low income countr*" or "low income nation?" or "low income population?" or "lower income countr*" or "lower income nation?" or "lower income population?" or "underserved countr*" or "underserved nation?" or "underserved population?" or "underserved world" or "under served countr*" or "under served nation?" or "under served population?" or "under served world" or "deprived countr*" or "deprived nation?" or "deprived population?" or "deprived world" or "poor countr*" or "poor nation?" or "poor population?" or "poor world" or "poorer countr*" or "poorer nation?" or "poorer population?" or "poorer world" or "developing econom*" or "less developed econom*" or "lesser developed econom*" or "under developed econom*" or "underdeveloped econom*" or "middle income econom*" or "low income econom*" or "lower income econom*" or "low gdp" or "low gnp" or "low gross domestic" or "low gross national" or "lower gdp" or "lower gnp" or "lower gross domestic" or "lower gross national" or lmic or lmics or "third world" or "lami countr*" or "transitional countr*" or "emerging economies" or "emerging nation?"))

**Social Sciences Citation Index & Arts & Humanities Index (Web of Science)—Revised Draft search**—**15th December 2020**

# 33 **47,214**

#32 AND #31

Indexes=SSCI, A&HCI Timespan=2000‐2020

# 32 3,306,731

**TS=((project* or program* or stud* or initiative* or activit* or invest* or experiment* or policy or policies or workshop* or impact* or assess* or measur* or implement* or scheme*))**

# 31 **47,907**

**#30 OR #8**

# 30 44,480

#29 AND #5 AND #4 AND #3

# 29 1,548,605

#28 OR #27 OR #26 OR #25 OR #24 OR #23 OR #22 OR #21 OR #20 OR #19 OR #18 OR #17 OR #16 OR #15 OR #14 OR #13 OR #12 OR #11 OR #10 OR #9

# 28 96

TS=((disarm* or demobili* or DDR) NEAR/3 (policy or policies or process* or ((female* or women) NEAR/2 (combatant* or soldier*))))

# 27 12,603

TS=((disaster* or hazard*) NEAR/3 (vulnerab* or risk* or manag* or mitigat* or response* or responsiveness or respond*))

# 26 3

TS=((displaced NEAR/2 (population* or people or communit* or women)) NEAR/3 (reintegrat* or re‐integrat* or "economic support" or cash‐based or "cash transfer*" or voucher* or inclus*))

# 25 326

TS=(peace NEAR/4 educat*)

# 24 10,787

TS=((mediat* or negotiat* or resolv* or resolution or prevent* or transform* or (capacity NEAR/2 build*)) NEAR/3 conflict*)

# 23 5

TS=(formal NEAR/3 ("peace process" or peacebuilding))

# 22 22,928

TS=(((dialog* or discuss* or consultative or consultation*) NEAR/2 (group* or forum* or communit* or subnational or local or regional)))

# 21 16

TS=((conflict or conflicts) NEAR/3 ("early warning"))

# 20 15

TS=((("gender‐based violence" or "sexual violence" or SGBV or crime) NEAR/3 ((accountab* NEAR/3 (perpetrat* or offender*)) or ((system* or capacity) NEAR/2 build*))))

# 19 2,379

TS=((((justice or judicial or legal) NEAR/3 (process* or procedure*)) NEAR/2 informal) or (dispute* NEAR/2 (resolv* or resolution)))

# 18 2,523

TS=((((capacity NEAR/2 build*) or support* or "gender sensitive" or treatment or "best practice*") NEAR/3 (police or policing)) or (crime* NEAR/3 report*) or (victim* NEAR/2 protect*))

# 17 28

TS=(((capacity NEAR/2 build*) NEAR/3 ((subnational or sub‐national or local or regional) NEAR/2 (government or authorit*)) or "gender equality offic"))

# 16 8,744

TS=(((incidence or prevalen*) NEAR/2 violence) or (("gender‐based violence" or "sexual violence" or SGBV or crime) NEAR/2 (mitigat* or prevent*)) or (vulnerable NEAR/3 group*))

# 15 66,301

TS=(((behavio* or attitud* or belief*) NEAR/2 chang*) or (role NEAR/3 (societ* or communit*)) or "social norm*" or "gender norm*" or ("social institution*" NEAR/3 structur*) or ((family or families) NEAR/3 train*) or (media NEAR/3 intervention*))

# 14 2,021

TS=((("social protection*" or (capacity NEAR/2 build*) or disseminat* or understanding or aware* or advocacy or "gender‐based violence" or "sexual violence" or SGBV)) NEAR/3 rights)

# 13 410,711

TS=((capacity NEAR/3 build*) or politics or political or civic or (communit* NEAR/3 develop*) or ((local or regional) NEAR/3 (government* or governance or quota or quotas or accountab*)) or ((outreach or engag*) NEAR/3 campaign*) or (enabl* NEAR/3 (environment* or condition*)) or ((women* or girl* or communit*) NEAR/2 right*))

# 12 207,439

TS=(leisure or sport* or art or theatr* or drama)

# 11 113,394

TS=(MSME or MSMEs or (micro NEAR/2 small NEAR/3 enterprise*) or loan or loans or "technical support" or ((entrepreneur* or enterprise*) NEAR/3 train*) or mentor* or start‐up* or CCT or CCTs or "conditional cash transfer*" or ((cash or financ*) NEAR/2 based NEAR/3 intervention*) or ((participat* or involv*) NEAR/3 economy) or (access* NEAR/3 credit) or microcredit or micro‐credit or "micro credit" or savings or insurance or "financial product*" or (self‐help NEAR/2 (group* or club* or organisation* or organization* or association*)))

# 10 843,675

TS=(school* or pedagog* or literacy or literate or illiterate or numeracy or numerate or curricul* or education* or teach* or voucher* or vocational or training or ((skill* or knowledge) NEAR/3 (work or employ* or job or jobs)))

# 9 43,317

TS=("civil societ*" or CSO or CSOs or ((economic* or financ*) NEAR/2 (partner* or associate*)) or cooperative* or co‐operative* or "land right*")

**# 8 8,602**

**#7 AND #4 AND #3**

# 7 75,480

#6 AND #5

# 6 464,772

TS=(empower* or disempower* or autonomy or (decision* NEAR/2 (make or made or making)) or self‐determin* or bargain* or negotiat* or equality or agency or transformati*)

# 5 863,144

TS=(gender* or woman* or women* or mother* or maternal or female* or wife* or wives or girl* or schoolgirl* or daughter* **or sex***)

# 4 1,624,586

TS=("quasi experiment*" or quasi‐experiment* or "random* control* trial*" or "random* trial*" or rct* or (random* NEAR/3 allocat*) or evaluat* or impact* or assess* or dif‐dif or psm or "double difference" or difference‐in‐difference or rdd or "difference in difference" or "statistical matching*" or "propensity score matching" or "covariate matching" or "coarsened‐exact matching" or "propensity‐weighted" or "multiple regression" or "statistical regression" or "regression discontinuity*" or "cohort analysis" or "quantitative method*" or "program* evaluation" or "interrupted time series" or (before NEAR/5 after) or (pre NEAR/5 post) or ((pretest or "pre test") and (posttest or "post test")) or (("fixed effect*" or "random effect*") NEAR/3 (model or estimation)) or "instrumental variable" or "synthetic control" or ((quantitative or "comparison group*" or counterfactual or "counter factual" or counter‐factual or experiment*) NEAR/3 (design or study or analysis)) or ((cost* or economic) NEAR/2 (benefit or effective* or analy*)))

# 3 1,380,811

#2 OR #1

# 2 995,675

CU=((afghanistan or albania or algeria or "american samoa" or angola or "antigua and barbuda" or antigua or barbuda or argentina or armenia or armenian or aruba or azerbaijan or bahrain or bangladesh or barbados or republic of belarus or belarus or byelarus or belorussia or byelorussian or belize or "british honduras" or benin or dahomey or bhutan or bolivia or "bosnia and herzegovina" or bosnia or herzegovina or botswana or bechuanaland or brazil or brasil or bulgaria or "burkina faso" or "burkina fasso" or "upper volta" or burundi or urundi or "cabo verde" or "cape verde" or cambodia or kampuchea or "khmer republic" or cameroon or cameron or cameroun or "central african republic" or "ubangi shari" or chad or chile or china or colombia or comoros or comoro islands or "iles comores" or mayotte or "democratic republic of the congo" or "democratic republic congo" or congo or zaire or "costa rica" or "cote divoire" or "cote d ivoire" or "cote divoire" or "cote d ivoire" or "ivory coast" or croatia or cuba or cyprus or "czech republic" or czechoslovakia or djibouti or "french somaliland" or dominica or "dominican republic" or ecuador or egypt or "united arab republic" or "el salvador" or "equatorial guinea" or "spanish guinea" or eritrea or estonia or eswatini or swaziland or ethiopia or fiji or gabon or "gabonese republic" or gambia or "georgia (republic) " or georgian or ghana or "gold coast" or gibraltar or greece or grenada or guam or guatemala or guinea or "guinea bissau" or guyana or "british guiana" or haiti or hispaniola or honduras or hungary or india or indonesia or timor or iran or iraq or "isle of man" or jamaica or jordan or kazakhstan or kazakh or kenya or "democratic peoples republic of korea" or "republic of korea" or "north korea" or "south korea" or korea or kosovo or kyrgyzstan or kirghizia or kirgizstan or "kyrgyz republic" or kirghiz or laos or "lao pdr" or "lao people's democratic republic" or latvia or lebanon or "lebanese republic" or lesotho or basutoland or liberia or libya or "libyan arab jamahiriya" or lithuania or macau or macao or "republic of north macedonia" or macedonia or madagascar or "malagasy republic" or malawi or nyasaland or malaysia or "malay federation" or "malaya federation" or maldives or "indian ocean islands" or "indian ocean" or mali or malta or micronesia or "federated states of micronesia" or kiribati or "marshall islands" or nauru or "northern mariana islands" or palau or tuvalu or mauritania or mauritius or mexico or moldova or moldovian or mongolia or montenegro or morocco or ifni or mozambique or "portuguese east africa" or myanmar or burma or namibia or nepal or "netherlands antilles" or nicaragua or niger or nigeria or oman or muscat or pakistan or panama or "papua new guinea" or "new guinea" or paraguay or peru or philippines or philipines or phillipines or phillippines or poland or "polish people's republic" or portugal or "portuguese republic" or "puerto rico" or romania or russia or "russian federation" or ussr or "soviet union or union of soviet socialist republics" or rwanda or ruanda or samoa or "pacific islands" or polynesia or "samoan islands" or "navigator island" or "navigator islands" or "sao tome and principe" or "saudi arabia" or senegal or serbia or seychelles or "sierra leone" or slovakia or "slovak republic" or slovenia or melanesia or "solomon island" or "solomon islands" or "norfolk island" or "norfolk islands" or somalia or "south africa" or "south sudan" or "sri lanka" or ceylon or "saint kitts and nevis" or "st. kitts and nevis" or "saint lucia" or "st. lucia" or "saint vincent and the grenadines" or "saint vincent" or "st. vincent" or grenadines or sudan or suriname or surinam or "dutch guiana" or "netherlands guiana" or syria or "syrian arab republic" or tajikistan or tadjikistan or tadzhikistan or tadzhik or tanzania or tanganyika or thailand or siam or "timor leste" or "east timor" or togo or "togolese republic" or tonga or "trinidad and tobago" or trinidad or tobago or tunisia or turkey or turkmenistan or turkmen or uganda or ukraine or uruguay or uzbekistan or uzbek or vanuatu or "new hebrides" or venezuela or vietnam or "viet nam" or "middle east" or "west bank" or gaza or palestine or yemen or yugoslavia or zambia or zimbabwe or "northern rhodesia" or "global south" or "africa south of the sahara" or "sub‐saharan africa" or "subsaharan africa" or "africa, central" or "central africa" or "africa, northern" or "north africa" or "northern africa" or magreb or maghrib or sahara or "africa, southern" or "southern africa" or "africa, eastern" or "east africa" or "eastern africa" or "africa, western" or "west africa" or "western africa" or "west indies" or "indian ocean islands" or caribbean or "central america" or "latin america" or "south and central america" or "south america" or "asia, central" or "central asia" or "asia, northern" or "north asia" or "northern asia" or "asia, southeastern" or "southeastern asia" or "south eastern asia" or "southeast asia" or "south east asia" or "asia, western" or "western asia" or "europe, eastern" or "east europe" or "eastern europe" or "developing country" or "developing countries" or "developing nation?" or "developing population?" or "developing world" or "less developed countr*" or "less developed nation?" or "less developed population?" or "less developed world" or "lesser developed countr*" or "lesser developed nation?" or "lesser developed population?" or "lesser developed world" or "under developed countr*" or "under developed nation?" or "under developed population?" or "under developed world" or "underdeveloped countr*" or "underdeveloped nation?" or "underdeveloped population?" or "underdeveloped world" or "middle income countr*" or "middle income nation?" or "middle income population?" or "low income countr*" or "low income nation?" or "low income population?" or "lower income countr*" or "lower income nation?" or "lower income population?" or "underserved countr*" or "underserved nation?" or "underserved population?" or "underserved world" or "under served countr*" or "under served nation?" or "under served population?" or "under served world" or "deprived countr*" or "deprived nation?" or "deprived population?" or "deprived world" or "poor countr*" or "poor nation?" or "poor population?" or "poor world" or "poorer countr*" or "poorer nation?" or "poorer population?" or "poorer world" or "developing econom*" or "less developed econom*" or "lesser developed econom*" or "under developed econom*" or "underdeveloped econom*" or "middle income econom*" or "low income econom*" or "lower income econom*" or "low gdp" or "low gnp" or "low gross domestic" or "low gross national" or "lower gdp" or "lower gnp" or "lower gross domestic" or "lower gross national" or lmic or lmics or "third world" or "lami countr*" or "transitional countr*" or "emerging economies" or "emerging nation?"))

# 1 791,798

TS=((afghanistan or albania or algeria or "american samoa" or angola or "antigua and barbuda" or antigua or barbuda or argentina or armenia or armenian or aruba or azerbaijan or bahrain or bangladesh or barbados or republic of belarus or belarus or byelarus or belorussia or byelorussian or belize or "british honduras" or benin or dahomey or bhutan or bolivia or "bosnia and herzegovina" or bosnia or herzegovina or botswana or bechuanaland or brazil or brasil or bulgaria or "burkina faso" or "burkina fasso" or "upper volta" or burundi or urundi or "cabo verde" or "cape verde" or cambodia or kampuchea or "khmer republic" or cameroon or cameron or cameroun or "central african republic" or "ubangi shari" or chad or chile or china or colombia or comoros or comoro islands or "iles comores" or mayotte or "democratic republic of the congo" or "democratic republic congo" or congo or zaire or "costa rica" or "cote divoire" or "cote d ivoire" or "cote divoire" or "cote d ivoire" or "ivory coast" or croatia or cuba or cyprus or "czech republic" or czechoslovakia or djibouti or "french somaliland" or dominica or "dominican republic" or ecuador or egypt or "united arab republic" or "el salvador" or "equatorial guinea" or "spanish guinea" or eritrea or estonia or eswatini or swaziland or ethiopia or fiji or gabon or "gabonese republic" or gambia or "georgia (republic) " or georgian or ghana or "gold coast" or gibraltar or greece or grenada or guam or guatemala or guinea or "guinea bissau" or guyana or "british guiana" or haiti or hispaniola or honduras or hungary or india or indonesia or timor or iran or iraq or "isle of man" or jamaica or jordan or kazakhstan or kazakh or kenya or "democratic peoples republic of korea" or "republic of korea" or "north korea" or "south korea" or korea or kosovo or kyrgyzstan or kirghizia or kirgizstan or "kyrgyz republic" or kirghiz or laos or "lao pdr" or "lao people's democratic republic" or latvia or lebanon or "lebanese republic" or lesotho or basutoland or liberia or libya or "libyan arab jamahiriya" or lithuania or macau or macao or "republic of north macedonia" or macedonia or madagascar or "malagasy republic" or malawi or nyasaland or malaysia or "malay federation" or "malaya federation" or maldives or "indian ocean islands" or "indian ocean" or mali or malta or micronesia or "federated states of micronesia" or kiribati or "marshall islands" or nauru or "northern mariana islands" or palau or tuvalu or mauritania or mauritius or mexico or moldova or moldovian or mongolia or montenegro or morocco or ifni or mozambique or "portuguese east africa" or myanmar or burma or namibia or nepal or "netherlands antilles" or nicaragua or niger or nigeria or oman or muscat or pakistan or panama or "papua new guinea" or "new guinea" or paraguay or peru or philippines or philipines or phillipines or phillippines or poland or "polish people's republic" or portugal or "portuguese republic" or "puerto rico" or romania or russia or "russian federation" or ussr or "soviet union or union of soviet socialist republics" or rwanda or ruanda or samoa or "pacific islands" or polynesia or "samoan islands" or "navigator island" or "navigator islands" or "sao tome and principe" or "saudi arabia" or senegal or serbia or seychelles or "sierra leone" or slovakia or "slovak republic" or slovenia or melanesia or "solomon island" or "solomon islands" or "norfolk island" or "norfolk islands" or somalia or "south africa" or "south sudan" or "sri lanka" or ceylon or "saint kitts and nevis" or "st. kitts and nevis" or "saint lucia" or "st. lucia" or "saint vincent and the grenadines" or "saint vincent" or "st. vincent" or grenadines or sudan or suriname or surinam or "dutch guiana" or "netherlands guiana" or syria or "syrian arab republic" or tajikistan or tadjikistan or tadzhikistan or tadzhik or tanzania or tanganyika or thailand or siam or "timor leste" or "east timor" or togo or "togolese republic" or tonga or "trinidad and tobago" or trinidad or tobago or tunisia or turkey or turkmenistan or turkmen or uganda or ukraine or uruguay or uzbekistan or uzbek or vanuatu or "new hebrides" or venezuela or vietnam or "viet nam" or "middle east" or "west bank" or gaza or palestine or yemen or yugoslavia or zambia or zimbabwe or "northern rhodesia" or "global south" or "africa south of the sahara" or "sub‐saharan africa" or "subsaharan africa" or "africa, central" or "central africa" or "africa, northern" or "north africa" or "northern africa" or magreb or maghrib or sahara or "africa, southern" or "southern africa" or "africa, eastern" or "east africa" or "eastern africa" or "africa, western" or "west africa" or "western africa" or "west indies" or "indian ocean islands" or caribbean or "central america" or "latin america" or "south and central america" or "south america" or "asia, central" or "central asia" or "asia, northern" or "north asia" or "northern asia" or "asia, southeastern" or "southeastern asia" or "south eastern asia" or "southeast asia" or "south east asia" or "asia, western" or "western asia" or "europe, eastern" or "east europe" or "eastern europe" or "developing country" or "developing countries" or "developing nation?" or "developing population?" or "developing world" or "less developed countr*" or "less developed nation?" or "less developed population?" or "less developed world" or "lesser developed countr*" or "lesser developed nation?" or "lesser developed population?" or "lesser developed world" or "under developed countr*" or "under developed nation?" or "under developed population?" or "under developed world" or "underdeveloped countr*" or "underdeveloped nation?" or "underdeveloped population?" or "underdeveloped world" or "middle income countr*" or "middle income nation?" or "middle income population?" or "low income countr*" or "low income nation?" or "low income population?" or "lower income countr*" or "lower income nation?" or "lower income population?" or "underserved countr*" or "underserved nation?" or "underserved population?" or "underserved world" or "under served countr*" or "under served nation?" or "under served population?" or "under served world" or "deprived countr*" or "deprived nation?" or "deprived population?" or "deprived world" or "poor countr*" or "poor nation?" or "poor population?" or "poor world" or "poorer countr*" or "poorer nation?" or "poorer population?" or "poorer world" or "developing econom*" or "less developed econom*" or "lesser developed econom*" or "under developed econom*" or "underdeveloped econom*" or "middle income econom*" or "low income econom*" or "lower income econom*" or "low gdp" or "low gnp" or "low gross domestic" or "low gross national" or "lower gdp" or "lower gnp" or "lower gross domestic" or "lower gross national" or lmic or lmics or "third world" or "lami countr*" or "transitional countr*" or "emerging economies" or "emerging nation?"))

**Social Sciences Citation Index & Arts & Humanities Index (Web of Science)**—**Draft search**—**11th December 2020**

# 31 **4,878**

#29 AND #8

Indexes=SSCI, A&HCI Timespan=2000‐2020

# 30 40,327

#29 AND #5 AND #4 AND #3

**# 29 1,547,443**

#28 OR #27 OR #26 OR #25 OR #24 OR #23 OR #22 OR #21 OR #20 OR #19 OR #18 OR #17 OR #16 OR #15 OR #14 OR #13 OR #12 OR #11 OR #10 OR #9

# 28 96

TS=((disarm* or demobili* or DDR) NEAR/3 (policy or policies or process* or ((female* or women)

# 27 12,591

TS=((disaster* or hazard*) NEAR/3 (vulnerab* or risk* or manag* or mitigat* or response* or responsiveness or respond*))

# 26 3

TS=((displaced NEAR/2 (population* or people or communit* or women)) NEAR/3 (reintegrat* or re‐integrat* or "economic support" or cash‐based or "cash transfer*" or voucher* or inclus*))

# 25 326

TS=(peace NEAR/4 educat*)

# 24 10,776

TS=((mediat* or negotiat* or resolv* or resolution or prevent* or transform* or (capacity NEAR/2 build*)) NEAR/3 conflict*)

# 23 5

TS=(formal NEAR/3 ("peace process" or peacebuilding))

# 22 22,904

TS=(((dialog* or discuss* or consultative or consultation*) NEAR/2 (group* or forum* or communit* or subnational or local or regional)))

# 21 16

TS=((conflict or conflicts) NEAR/3 ("early warning"))

# 20 15

TS=((("gender‐based violence" or "sexual violence" or SGBV or crime) NEAR/3 ((accountab* NEAR/3 (perpetrat* or offender*)) or ((system* or capacity) NEAR/2 build*))))

# 19 2,378

TS=((((justice or judicial or legal) NEAR/3 (process* or procedure*)) NEAR/2 informal) or (dispute* NEAR/2 (resolv* or resolution)))

# 18 2,519

TS=((((capacity NEAR/2 build*) or support* or "gender sensitive" or treatment or "best practice*") NEAR/3 (police or policing)) or (crime* NEAR/3 report*) or (victim* NEAR/2 protect*))

# 17 28

TS=(((capacity NEAR/2 build*) NEAR/3 ((subnational or sub‐national or local or regional) NEAR/2 (government or authorit*)) or "gender equality offic"))

# 16 8,727

TS=(((incidence or prevalen*) NEAR/2 violence) or (("gender‐based violence" or "sexual violence" or SGBV or crime) NEAR/2 (mitigat* or prevent*)) or (vulnerable NEAR/3 group*))

# 15 66,229

TS=(((behavio* or attitud* or belief*) NEAR/2 chang*) or (role NEAR/3 (societ* or communit*)) or "social norm*" or "gender norm*" or ("social institution*" NEAR/3 structur*) or ((family or families) NEAR/3 train*) or (media NEAR/3 intervention*))

# 14 2,020

TS=((("social protection*" or (capacity NEAR/2 build*) or disseminat* or understanding or aware* or advocacy or "gender‐based violence" or "sexual violence" or SGBV)) NEAR/3 rights)

# 13 410,399

TS=((capacity NEAR/3 build*) or politics or political or civic or (communit* NEAR/3 develop*) or ((local or regional) NEAR/3 (government* or governance or quota or quotas or accountab*)) or ((outreach or engag*) NEAR/3 campaign*) or (enabl* NEAR/3 (environment* or condition*)) or ((women* or girl* or communit*) NEAR/2 right*))

# 12 207,308

TS=(leisure or sport* or art or theatr* or drama)

# 11 113,297

TS=(MSME or MSMEs or (micro NEAR/2 small NEAR/3 enterprise*) or loan or loans or "technical support" or ((entrepreneur* or enterprise*) NEAR/3 train*) or mentor* or start‐up* or CCT or CCTs or "conditional cash transfer*" or ((cash or financ*) NEAR/2 based NEAR/3 intervention*) or ((participat* or involv*) NEAR/3 economy) or (access* NEAR/3 credit) or microcredit or micro‐credit or "micro credit" or savings or insurance or "financial product*" or (self‐help NEAR/2 (group* or club* or organisation* or organization* or association*)))

# 10 843,042

TS=(school* or pedagog* or literacy or literate or illiterate or numeracy or numerate or curricul* or education* or teach* or voucher* or vocational or training or ((skill* or knowledge) NEAR/3 (work or employ* or job or jobs)))

# 9 43,290

TS=("civil societ*" or CSO or CSOs or ((economic* or financ*) NEAR/2 (partner* or associate*)) or cooperative* or co‐operative* or "land right*")

# 8 8,065

#7 AND #4 AND #3

# 7 68,615

#6 AND #5

**# 6 464,392**

**TS=(empower* or disempower* or autonomy or (decision* NEAR/2 (make or made or making)) or self‐determin* or bargain* or negotiat* or equality or agency or transformati*)**

**# 5 753,268**

**TS=(gender* or woman* or women* or mother* or maternal or female* or wife* or wives or girl* or schoolgirl* or daughter*)**

# 4 1,622,959

TS=("quasi experiment*" or quasi‐experiment* or "random* control* trial*" or "random* trial*" or rct* or (random* NEAR/3 allocat*) or evaluat* or impact* or assess* or dif‐dif or psm or "double difference" or difference‐in‐difference or rdd or "difference in difference" or "statistical matching*" or "propensity score matching" or "covariate matching" or "coarsened‐exact matching" or "propensity‐weighted" or "multiple regression" or "statistical regression" or "regression discontinuity*" or "cohort analysis" or "quantitative method*" or "program* evaluation" or "interrupted time series" or (before NEAR/5 after) or (pre NEAR/5 post) or ((pretest or "pre test") and (posttest or "post test")) or (("fixed effect*" or "random effect*") NEAR/3 (model or estimation)) or "instrumental variable" or "synthetic control" or ((quantitative or "comparison group*" or counterfactual or "counter factual" or counter‐factual or experiment*) NEAR/3 (design or study or analysis)) or ((cost* or economic) NEAR/2 (benefit or effective* or analy*)))

# 3 1,379,547

#2 OR #1

# 2 994,588

CU=((afghanistan or albania or algeria or "american samoa" or angola or "antigua and barbuda" or antigua or barbuda or argentina or armenia or armenian or aruba or azerbaijan or bahrain or bangladesh or barbados or republic of belarus or belarus or byelarus or belorussia or byelorussian or belize or "british honduras" or benin or dahomey or bhutan or bolivia or "bosnia and herzegovina" or bosnia or herzegovina or botswana or bechuanaland or brazil or brasil or bulgaria or "burkina faso" or "burkina fasso" or "upper volta" or burundi or urundi or "cabo verde" or "cape verde" or cambodia or kampuchea or "khmer republic" or cameroon or cameron or cameroun or "central african republic" or "ubangi shari" or chad or chile or china or colombia or comoros or comoro islands or "iles comores" or mayotte or "democratic republic of the congo" or "democratic republic congo" or congo or zaire or "costa rica" or "cote divoire" or "cote d ivoire" or "cote divoire" or "cote d ivoire" or "ivory coast" or croatia or cuba or cyprus or "czech republic" or czechoslovakia or djibouti or "french somaliland" or dominica or "dominican republic" or ecuador or egypt or "united arab republic" or "el salvador" or "equatorial guinea" or "spanish guinea" or eritrea or estonia or eswatini or swaziland or ethiopia or fiji or gabon or "gabonese republic" or gambia or "georgia (republic) " or georgian or ghana or "gold coast" or gibraltar or greece or grenada or guam or guatemala or guinea or "guinea bissau" or guyana or "british guiana" or haiti or hispaniola or honduras or hungary or india or indonesia or timor or iran or iraq or "isle of man" or jamaica or jordan or kazakhstan or kazakh or kenya or "democratic peoples republic of korea" or "republic of korea" or "north korea" or "south korea" or korea or kosovo or kyrgyzstan or kirghizia or kirgizstan or "kyrgyz republic" or kirghiz or laos or "lao pdr" or "lao people's democratic republic" or latvia or lebanon or "lebanese republic" or lesotho or basutoland or liberia or libya or "libyan arab jamahiriya" or lithuania or macau or macao or "republic of north macedonia" or macedonia or madagascar or "malagasy republic" or malawi or nyasaland or malaysia or "malay federation" or "malaya federation" or maldives or "indian ocean islands" or "indian ocean" or mali or malta or micronesia or "federated states of micronesia" or kiribati or "marshall islands" or nauru or "northern mariana islands" or palau or tuvalu or mauritania or mauritius or mexico or moldova or moldovian or mongolia or montenegro or morocco or ifni or mozambique or "portuguese east africa" or myanmar or burma or namibia or nepal or "netherlands antilles" or nicaragua or niger or nigeria or oman or muscat or pakistan or panama or "papua new guinea" or "new guinea" or paraguay or peru or philippines or philipines or phillipines or phillippines or poland or "polish people's republic" or portugal or "portuguese republic" or "puerto rico" or romania or russia or "russian federation" or ussr or "soviet union or union of soviet socialist republics" or rwanda or ruanda or samoa or "pacific islands" or polynesia or "samoan islands" or "navigator island" or "navigator islands" or "sao tome and principe" or "saudi arabia" or senegal or serbia or seychelles or "sierra leone" or slovakia or "slovak republic" or slovenia or melanesia or "solomon island" or "solomon islands" or "norfolk island" or "norfolk islands" or somalia or "south africa" or "south sudan" or "sri lanka" or ceylon or "saint kitts and nevis" or "st. kitts and nevis" or "saint lucia" or "st. lucia" or "saint vincent and the grenadines" or "saint vincent" or "st. vincent" or grenadines or sudan or suriname or surinam or "dutch guiana" or "netherlands guiana" or syria or "syrian arab republic" or tajikistan or tadjikistan or tadzhikistan or tadzhik or tanzania or tanganyika or thailand or siam or "timor leste" or "east timor" or togo or "togolese republic" or tonga or "trinidad and tobago" or trinidad or tobago or tunisia or turkey or turkmenistan or turkmen or uganda or ukraine or uruguay or uzbekistan or uzbek or vanuatu or "new hebrides" or venezuela or vietnam or "viet nam" or "middle east" or "west bank" or gaza or palestine or yemen or yugoslavia or zambia or zimbabwe or "northern rhodesia" or "global south" or "africa south of the sahara" or "sub‐saharan africa" or "subsaharan africa" or "africa, central" or "central africa" or "africa, northern" or "north africa" or "northern africa" or magreb or maghrib or sahara or "africa, southern" or "southern africa" or "africa, eastern" or "east africa" or "eastern africa" or "africa, western" or "west africa" or "western africa" or "west indies" or "indian ocean islands" or caribbean or "central america" or "latin america" or "south and central america" or "south america" or "asia, central" or "central asia" or "asia, northern" or "north asia" or "northern asia" or "asia, southeastern" or "southeastern asia" or "south eastern asia" or "southeast asia" or "south east asia" or "asia, western" or "western asia" or "europe, eastern" or "east europe" or "eastern europe" or "developing country" or "developing countries" or "developing nation?" or "developing population?" or "developing world" or "less developed countr*" or "less developed nation?" or "less developed population?" or "less developed world" or "lesser developed countr*" or "lesser developed nation?" or "lesser developed population?" or "lesser developed world" or "under developed countr*" or "under developed nation?" or "under developed population?" or "under developed world" or "underdeveloped countr*" or "underdeveloped nation?" or "underdeveloped population?" or "underdeveloped world" or "middle income countr*" or "middle income nation?" or "middle income population?" or "low income countr*" or "low income nation?" or "low income population?" or "lower income countr*" or "lower income nation?" or "lower income population?" or "underserved countr*" or "underserved nation?" or "underserved population?" or "underserved world" or "under served countr*" or "under served nation?" or "under served population?" or "under served world" or "deprived countr*" or "deprived nation?" or "deprived population?" or "deprived world" or "poor countr*" or "poor nation?" or "poor population?" or "poor world" or "poorer countr*" or "poorer nation?" or "poorer population?" or "poorer world" or "developing econom*" or "less developed econom*" or "lesser developed econom*" or "under developed econom*" or "underdeveloped econom*" or "middle income econom*" or "low income econom*" or "lower income econom*" or "low gdp" or "low gnp" or "low gross domestic" or "low gross national" or "lower gdp" or "lower gnp" or "lower gross domestic" or "lower gross national" or lmic or lmics or "third world" or "lami countr*" or "transitional countr*" or "emerging economies" or "emerging nation?"))

# 1 791,207

TS=((afghanistan or albania or algeria or "american samoa" or angola or "antigua and barbuda" or antigua or barbuda or argentina or armenia or armenian or aruba or azerbaijan or bahrain or bangladesh or barbados or republic of belarus or belarus or byelarus or belorussia or byelorussian or belize or "british honduras" or benin or dahomey or bhutan or bolivia or "bosnia and herzegovina" or bosnia or herzegovina or botswana or bechuanaland or brazil or brasil or bulgaria or "burkina faso" or "burkina fasso" or "upper volta" or burundi or urundi or "cabo verde" or "cape verde" or cambodia or kampuchea or "khmer republic" or cameroon or cameron or cameroun or "central african republic" or "ubangi shari" or chad or chile or china or colombia or comoros or comoro islands or "iles comores" or mayotte or "democratic republic of the congo" or "democratic republic congo" or congo or zaire or "costa rica" or "cote divoire" or "cote d ivoire" or "cote divoire" or "cote d ivoire" or "ivory coast" or croatia or cuba or cyprus or "czech republic" or czechoslovakia or djibouti or "french somaliland" or dominica or "dominican republic" or ecuador or egypt or "united arab republic" or "el salvador" or "equatorial guinea" or "spanish guinea" or eritrea or estonia or eswatini or swaziland or ethiopia or fiji or gabon or "gabonese republic" or gambia or "georgia (republic) " or georgian or ghana or "gold coast" or gibraltar or greece or grenada or guam or guatemala or guinea or "guinea bissau" or guyana or "british guiana" or haiti or hispaniola or honduras or hungary or india or indonesia or timor or iran or iraq or "isle of man" or jamaica or jordan or kazakhstan or kazakh or kenya or "democratic peoples republic of korea" or "republic of korea" or "north korea" or "south korea" or korea or kosovo or kyrgyzstan or kirghizia or kirgizstan or "kyrgyz republic" or kirghiz or laos or "lao pdr" or "lao people's democratic republic" or latvia or lebanon or "lebanese republic" or lesotho or basutoland or liberia or libya or "libyan arab jamahiriya" or lithuania or macau or macao or "republic of north macedonia" or macedonia or madagascar or "malagasy republic" or malawi or nyasaland or malaysia or "malay federation" or "malaya federation" or maldives or "indian ocean islands" or "indian ocean" or mali or malta or micronesia or "federated states of micronesia" or kiribati or "marshall islands" or nauru or "northern mariana islands" or palau or tuvalu or mauritania or mauritius or mexico or moldova or moldovian or mongolia or montenegro or morocco or ifni or mozambique or "portuguese east africa" or myanmar or burma or namibia or nepal or "netherlands antilles" or nicaragua or niger or nigeria or oman or muscat or pakistan or panama or "papua new guinea" or "new guinea" or paraguay or peru or philippines or philipines or phillipines or phillippines or poland or "polish people's republic" or portugal or "portuguese republic" or "puerto rico" or romania or russia or "russian federation" or ussr or "soviet union or union of soviet socialist republics" or rwanda or ruanda or samoa or "pacific islands" or polynesia or "samoan islands" or "navigator island" or "navigator islands" or "sao tome and principe" or "saudi arabia" or senegal or serbia or seychelles or "sierra leone" or slovakia or "slovak republic" or slovenia or melanesia or "solomon island" or "solomon islands" or "norfolk island" or "norfolk islands" or somalia or "south africa" or "south sudan" or "sri lanka" or ceylon or "saint kitts and nevis" or "st. kitts and nevis" or "saint lucia" or "st. lucia" or "saint vincent and the grenadines" or "saint vincent" or "st. vincent" or grenadines or sudan or suriname or surinam or "dutch guiana" or "netherlands guiana" or syria or "syrian arab republic" or tajikistan or tadjikistan or tadzhikistan or tadzhik or tanzania or tanganyika or thailand or siam or "timor leste" or "east timor" or togo or "togolese republic" or tonga or "trinidad and tobago" or trinidad or tobago or tunisia or turkey or turkmenistan or turkmen or uganda or ukraine or uruguay or uzbekistan or uzbek or vanuatu or "new hebrides" or venezuela or vietnam or "viet nam" or "middle east" or "west bank" or gaza or palestine or yemen or yugoslavia or zambia or zimbabwe or "northern rhodesia" or "global south" or "africa south of the sahara" or "sub‐saharan africa" or "subsaharan africa" or "africa, central" or "central africa" or "africa, northern" or "north africa" or "northern africa" or magreb or maghrib or sahara or "africa, southern" or "southern africa" or "africa, eastern" or "east africa" or "eastern africa" or "africa, western" or "west africa" or "western africa" or "west indies" or "indian ocean islands" or caribbean or "central america" or "latin america" or "south and central america" or "south america" or "asia, central" or "central asia" or "asia, northern" or "north asia" or "northern asia" or "asia, southeastern" or "southeastern asia" or "south eastern asia" or "southeast asia" or "south east asia" or "asia, western" or "western asia" or "europe, eastern" or "east europe" or "eastern europe" or "developing country" or "developing countries" or "developing nation?" or "developing population?" or "developing world" or "less developed countr*" or "less developed nation?" or "less developed population?" or "less developed world" or "lesser developed countr*" or "lesser developed nation?" or "lesser developed population?" or "lesser developed world" or "under developed countr*" or "under developed nation?" or "under developed population?" or "under developed world" or "underdeveloped countr*" or "underdeveloped nation?" or "underdeveloped population?" or "underdeveloped world" or "middle income countr*" or "middle income nation?" or "middle income population?" or "low income countr*" or "low income nation?" or "low income population?" or "lower income countr*" or "lower income nation?" or "lower income population?" or "underserved countr*" or "underserved nation?" or "underserved population?" or "underserved world" or "under served countr*" or "under served nation?" or "under served population?" or "under served world" or "deprived countr*" or "deprived nation?" or "deprived population?" or "deprived world" or "poor countr*" or "poor nation?" or "poor population?" or "poor world" or "poorer countr*" or "poorer nation?" or "poorer population?" or "poorer world" or "developing econom*" or "less developed econom*" or "lesser developed econom*" or "under developed econom*" or "underdeveloped econom*" or "middle income econom*" or "low income econom*" or "lower income econom*" or "low gdp" or "low gnp" or "low gross domestic" or "low gross national" or "lower gdp" or "lower gnp" or "lower gross domestic" or "lower gross national" or lmic or lmics or "third world" or "lami countr*" or "transitional countr*" or "emerging economies" or "emerging nation?"))

## APPENDIX G FULL TEXT SCREENING CHECKLIST

**Table:**

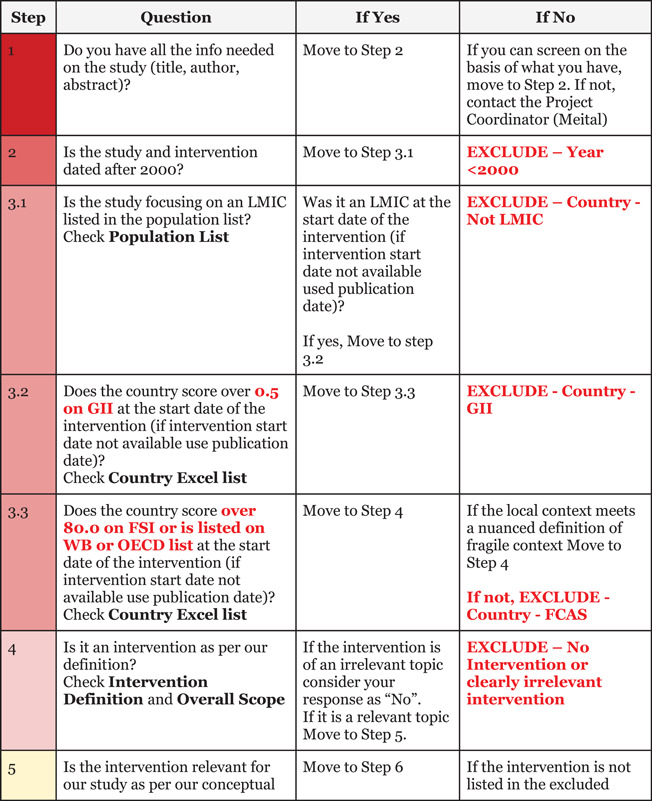

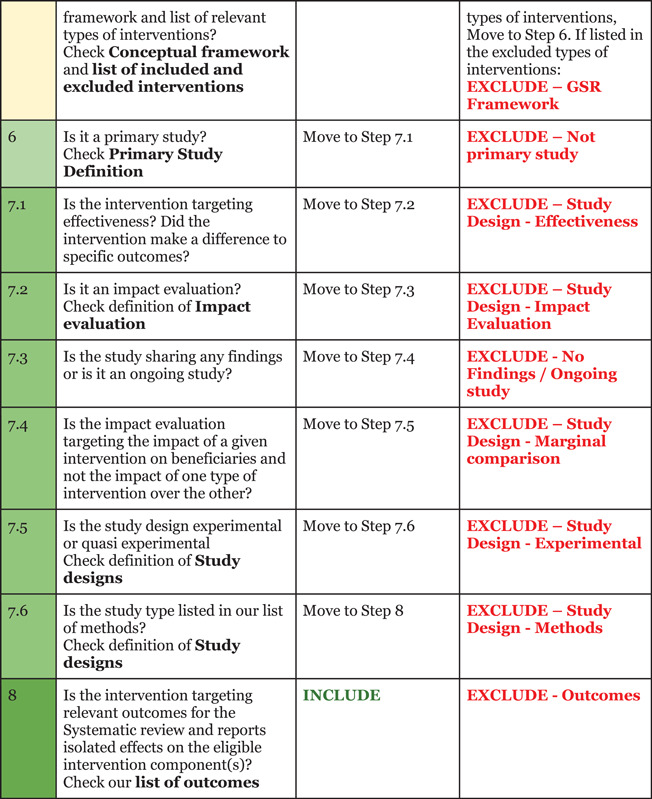

## APPENDIX H LIST OF OUTCOMES

**Table:**

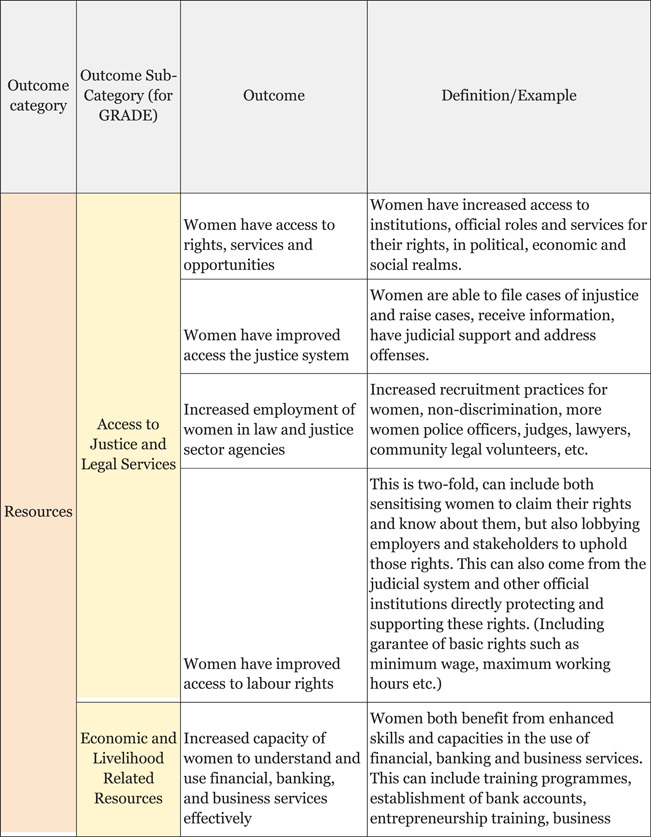

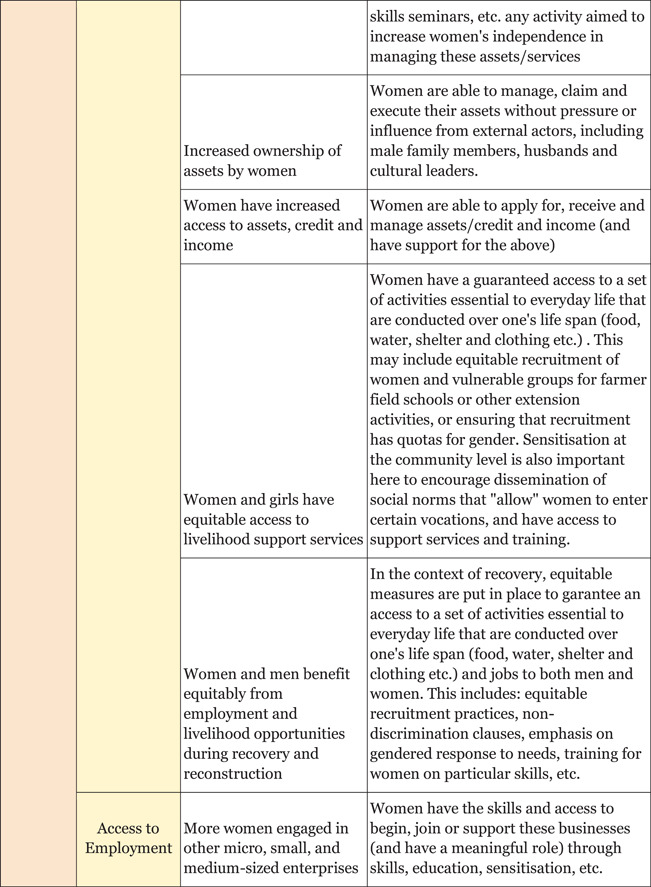

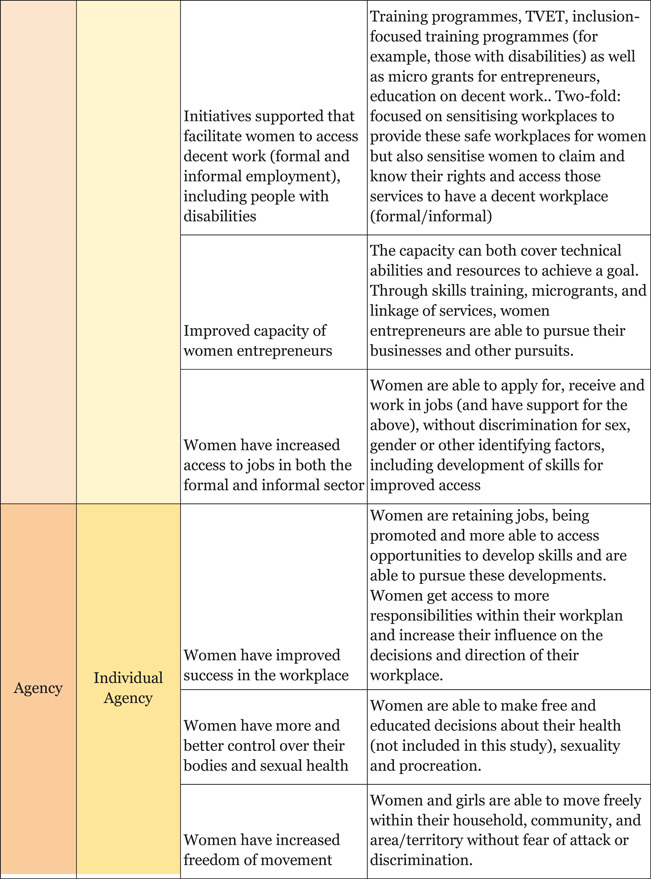

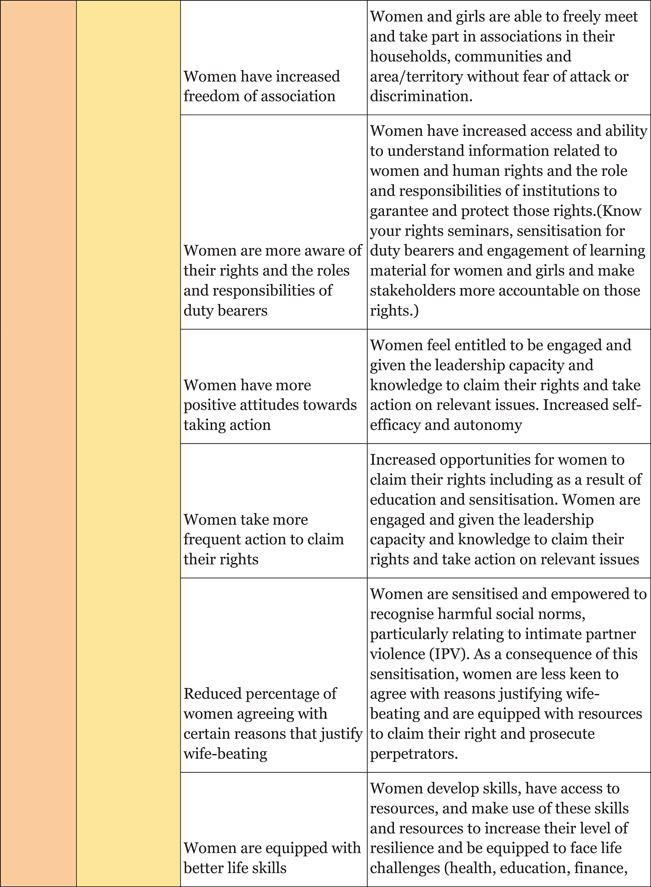

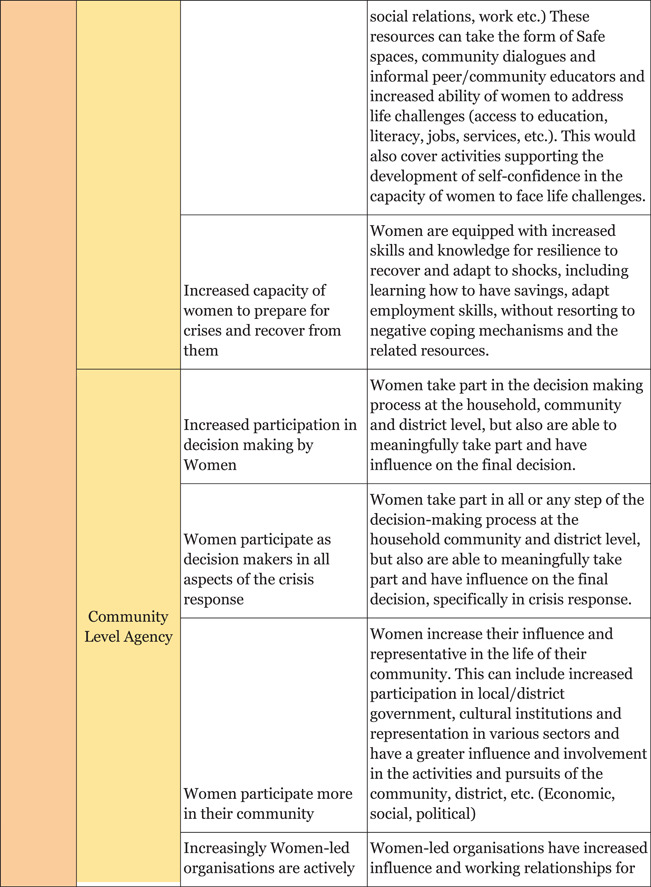

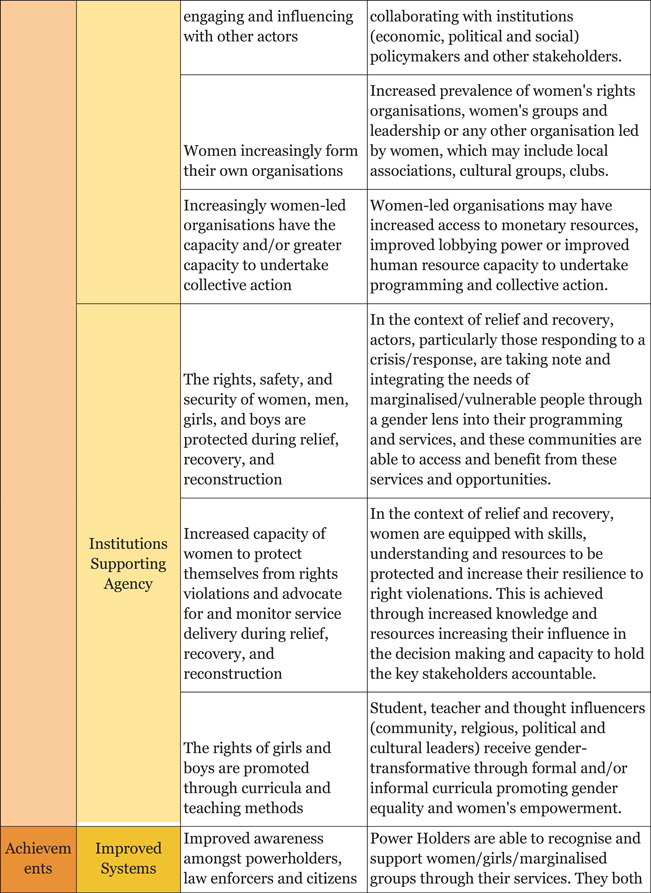

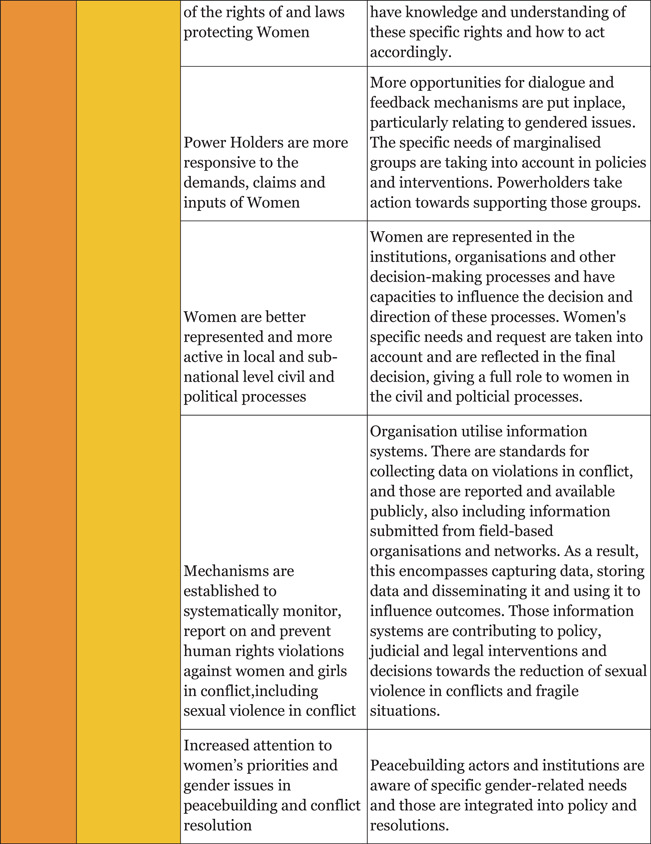

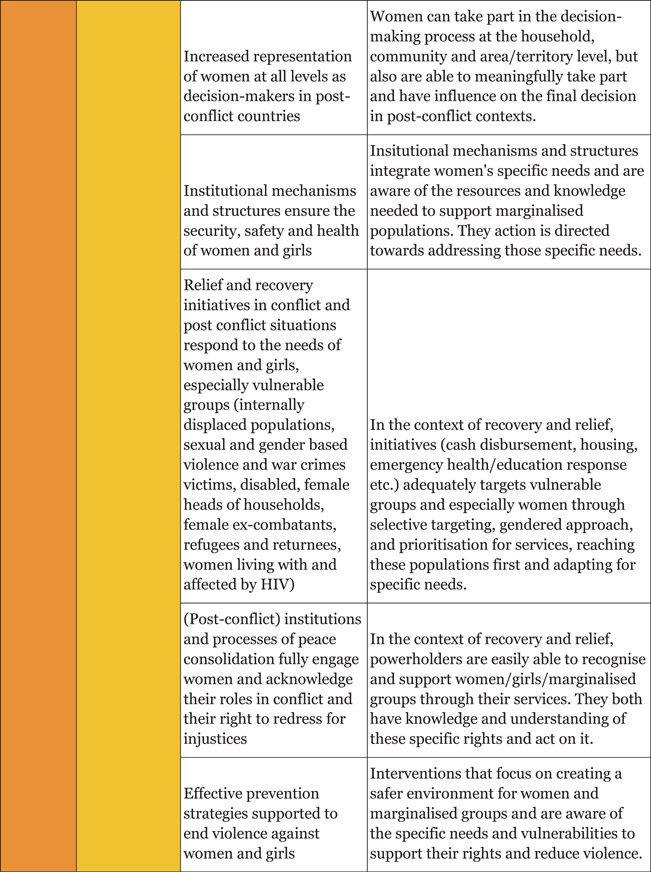

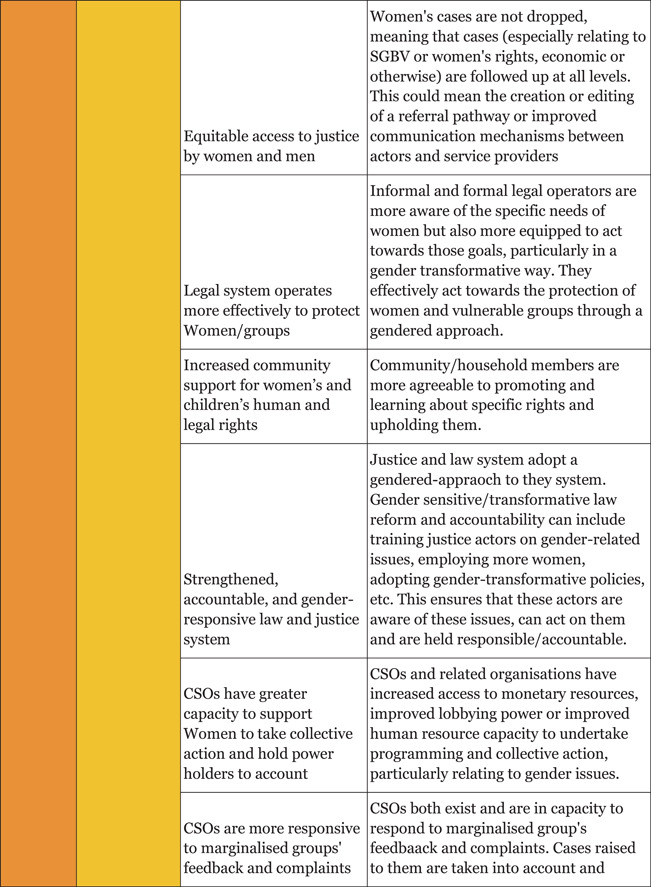

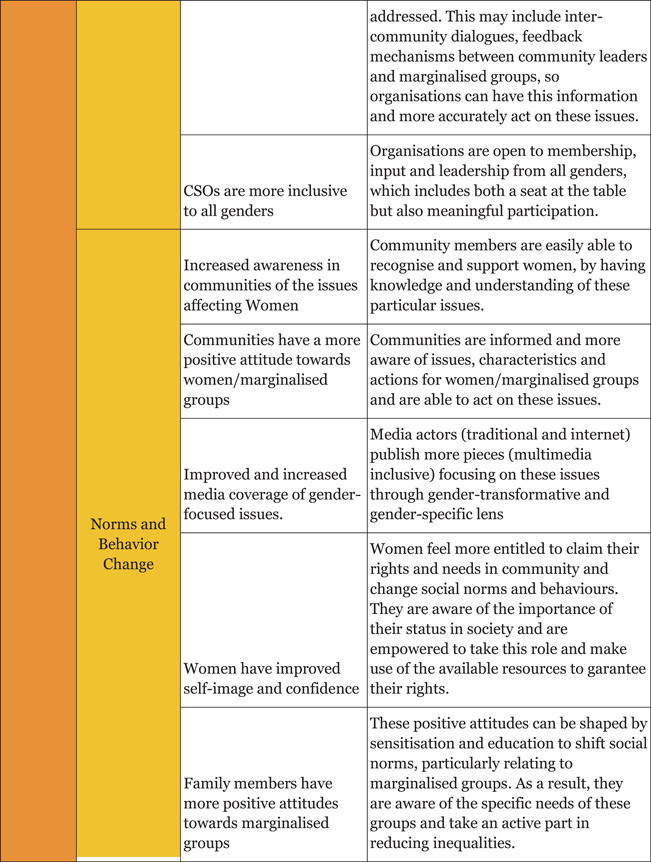

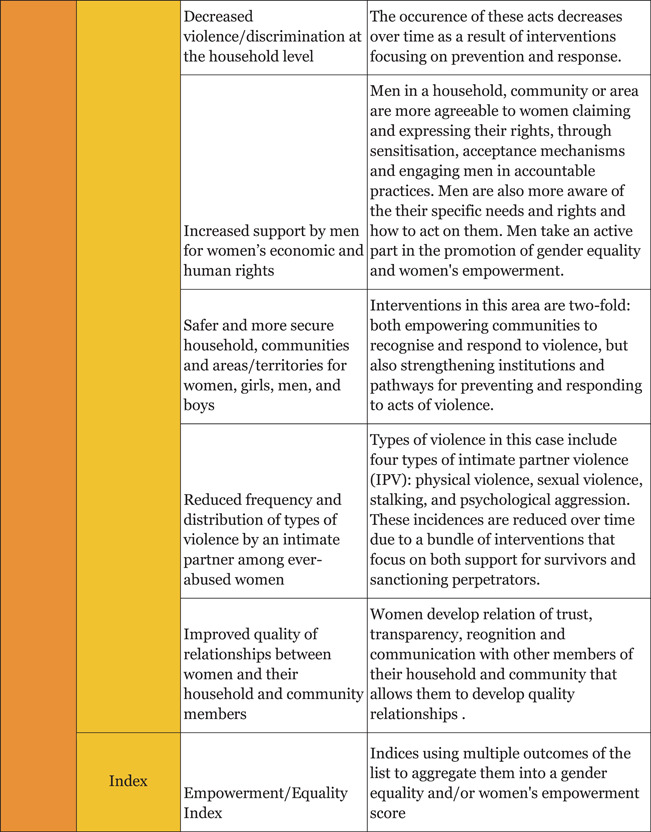

## APPENDIX I ADDITIONAL BACKGROUND INFORMATION

**Table I1:** Examples of National Plan for Action on UNSCR 1325

Country	National Plan for Action on UNSCR 1325
Mali	The Malian National Action Plan, published in 2012, emphasises participation and prevention but also integrates a specific focus on the question of refugees and internally displaced persons (IDPs) and sexual violence
Brazil	The Brazilian National Action Plan was published in 2017 with a dominant focus on the concepts of participation, prevention and protection of the 1325 and a specific focus on the raise of awareness and engagement for the WPS
Afghanistan	The Afghan NAP, introduced in 2015, has a specific focus on the notions of participation and protection but also gives a substantial importance to the role of the civil society to achieve the WPS goals
Philippines	The Philippines is now on its second NAP, which supports the ideas of protection, participation and prevention, but includes a specific focus on the role of civil society and humanitarian support for relief and recovery
Democratic Republic of the Congo	The DRC, now on its third NAP beginning in 2018, focuses primarily on the aspects of prevention and the questions of sexual violence and small arms
South Sudan	South Sudan currently has a NAP in place from 2015 to 2020, launched by the Ministry of Gender, Child and Social Welfare, particularly focuses on the lived realities of women and girls in conflict. However, despite South Sudan's NAP and signatory status to CEDAW and other instruments, implementation on the ground leaves much to be desired

**Table I2:** Chronology of the impact of UNSCR 1325

Year	Key step
2003	**The African Union's Protocol to the African Charter on Human and Peoples’ Rights on the Rights of Women in Africa (Maputo Protocol)** recognises that gender inequality undermines social, economic and political empowerment, which in turn impacts on human security and states that “Every woman shall have the right to dignity inherent in a human being and to the recognition and protection of her human and legal rights” and “Every woman shall be entitled to respect for her life and the integrity and security of her person. All forms of exploitation, cruel, inhuman or degrading punishment and treatment shall be prohibited” (Sigsworth & Kumalo, 2016)
2008	**UNSCR 1820** recognises sexual violance as a weapon and tactic of war and notes that rape and other forms of sexual violence can constitute a war crime, crime against humanity, or a consitutive act with respect to genocide The Council of the European Union (EU) adopted the **“Comprehensive approach to the EU implementation of the UNSCR 1325 and 1820”** promoting the implementation of the Women, Peace and Security (WPS) agenda
2009	**UNSCR 1888** reiterates that sexual violence exacerbates armed conflict and calls for leadership to address conflict‐related sexual violence **UNSCR 1889** focuses on post‐conflict peacebuilding and women's participation in all stages of peace processes and calls for indicators to measure the implementation of UNSCR 1325. It contains a seven‐point action plan on gender‐responsive peacebuilding, which included a target of 15% for UN peacebuilding allocations to be focused on gender equality and women's empowerment
2010	**UNSCR 1960** sets up a “naming and shaming” listing mechanism of countries not compliant with the UNSCR 1325
2013	**UNSCR 2122** underlines the need for an integrated approach to sustainable peace and calls for the provision of multisectoral services to women affected by conflicts and call for a global study on the implementation of UNSCR 1325 **The Convention on the Elimination of Discrimination Against Women (CEDAW) recommendation No.30** (Committee on the Elimination of Discrimination against Women, 2013) reinforces women's critical role in conflict prevention, peacebuilding and reconstruction processes and highlights the need for a concerted and integrated approach with the WPS based on a model of substantive equality
2015	**UNSCR 2250** identifies five key pillars for action and peace: participation, protection, prevention, partnerships and disengagement & reintegration **The 2030 Sustainable Development Goals (SDGs**) include a goal on gender equality and on Peace and Security Publication of the **Global Study on the implementation of UNSCR 1325**
2016	**UNSCR 2282** on the Review of the UN Peacebuilding Architecture (United Nations Security Council, 2016)
2018	**UNSCR 2419** recognises the positive role young people (including girls) can play in negotiating and implementing peace agreements and conflict prevention (United Nations Security Council, 2018) The EU adopted the **Strategic Approach to Women, Peace and Security**
2019	The African Union (AU) launches its Continental Results Framework on WPS in Africa
